# Definition of a new blood cell count score for early survival prediction for non-small cell lung cancer patients treated with atezolizumab: Integrated analysis of four multicenter clinical trials

**DOI:** 10.3389/fimmu.2022.961926

**Published:** 2022-09-02

**Authors:** Jian-Guo Zhou, Ada Hang-Heng Wong, Haitao Wang, Su-Han Jin, Fangya Tan, Yu-Zhong Chen, Si-Si He, Gang Shen, Benjamin Frey, Rainer Fietkau, Markus Hecht, Shamus R. Carr, Ruihong Wang, Bo Shen, David S. Schrump, Hu Ma, Udo S. Gaipl

**Affiliations:** ^1^ Department of Oncology, The Second Affiliated Hospital of Zunyi Medical University, Zunyi, China; ^2^ Department of Radiation Oncology, Translational Radiobiology, Universitätsklinikum Erlangen, Erlangen, Germany; ^3^ Department of Radiation Oncology, Universitätsklinikum Erlangen, Erlangen, Germany; ^4^ Comprehensive Cancer Center Erlangen-EMN, Erlangen, Germany; ^5^ AW Medical Co. Ltd., Macau, Macao SAR, China; ^6^ Thoracic Epigenetics Section, Thoracic Surgery Branch, Center for Cancer Research National Cancer Institute, National Institutes of Health, Bethesda, Maryland, United States; ^7^ Special Key Laboratory of Oral Diseases Research, Stomatological Hospital Affiliated to Zunyi Medical University, Zunyi, China; ^8^ Department of Analytics, Harrisburg University of Science and Technology, Harrisburg, PA, United States; ^9^ The Affiliated Cancer Hospital of Nanjing Medical University, Jiangsu Cancer Hospital and Jiangsu Institute of Cancer Research, Nanjing, China

**Keywords:** NSCLC, atezolizumab, biomarker, blood cell count, clinical trial

## Abstract

**Importance:**

Blood cell count test (BCT) is a robust method that provides direct quantification of various types of immune cells to reveal the immune landscape to predict atezolizumab treatment outcomes for clinicians to decide the next phase of treatment.

**Objective:**

This study aims to define a new BCTscore model to predict atezolizumab treatment benefits in non-small lung cell cancer (NSCLC) patients.

**Design, Setting, and Participants:**

This study analyzed four international, multicenter clinical trials (OAK, BIRCH, POPLAR, and FIR trials) to conduct *post-hoc* analyses of NSCLC patients undergoing atezolizumab (anti–PD-L1) single-agent treatment (*n* = 1,479) or docetaxel single-agent treatment (*n* = 707). BCT was conducted at three time points: pre-treatment (T1), the first day of treatment cycle 3 (T2), and first day of treatment cycle 5 (T3). Univariate and multivariate Cox regression analyses were conducted to identify early BCT biomarkers to predict atezolizumab treatment outcomes in NSCLC patients.

**Main Outcomes and Measures:**

Overall survival (OS) was used as the primary end point, whereas progression-free survival (PFS) according to Response Evaluation Criteria in Solid Tumors (RECIST), clinical benefit (CB), and objective response rate (ORR) were used as secondary end points.

**Results:**

The BCT biomarkers of neutrophil-to-lymphocyte ratio (NLR) and platelet-to-lymphocyte ratio (PLR) at time point T3 and neutrophil-to-monocyte ratio (NMR) at time point T2 with absolute cutoff values of NLR_T3 = 5, PLR_T3 = 180, and NMR_T2 = 6 were identified as strong predictive biomarkers for atezolizumab (Ate)–treated NSCLC patients in comparison with docetaxel (Dtx)–treated patients regarding OS (BCTscore low risk: HR _Ate_ vs_. Dtx_ = 1.54 (95% CI: 1.04–2.27), *P* = 0.031; high risk: HR _Ate_ vs_. Dtx_ = 0.84 (95% CI: 0.62–1.12), *P* = 0.235). The identified BCTscore model showed better OS AUC in the OAK (AUC_12month_ = 0.696), BIRCH (AUC_12month_ = 0.672) and POPLAR+FIR studies (AUC_12month_ = 0.727) than that of each of the three single BCT biomarkers.

**Conclusion and Relevance:**

The BCTscore model is a valid predictive and prognostic biomarker for early survival prediction in atezolizumab-treated NSCLC patients.

## Background

Non-small cell lung cancer (NSCLC) accounts for 84% of all lung cancer incidence, roughly accounting for 235,170 new cases in the United States in 2021 (1). Therapy for advanced NSCLC can include chemotherapy, radiotherapy, or tyrosine kinase inhibitors (TKIs) as first-line therapy for patients carrying genetic mutations in the genes of *EGFR*, *ALK*, *ROS1*, and *NTRK* (2). However, for patients without TKI-targeted mutations, safe and effective therapeutic options were limited. With the development of immune checkpoint inhibitors (ICIs), this has changed. ICIs were developed against programmed cell death ligand 1 (PD-L1) on cancer cells, such as atezolizumab, and the immune suppressive receptors programmed cell death 1 (PD-1), such as nivolumab and pembrolizumab, and cytotoxic T lymphocyte-associated antigen 4 (CTLA-4), such as ipilimumab, being present on cytotoxic T cells (2).

These therapies have improved NSCLC treatment outcomes in patients with advanced disease (3–5). However, without the implementation of patient selection by predictive or prognostic biomarkers, no significant PFS or OS improvement by ICI therapy, as compared with chemotherapy, is observed (6). PD-L1 expression and tumor mutational burden (TMB) are often recommended for patient selection before treatment, but contrasting results are seen in clinical trials involving atezolizumab (3, 4, 7) and nivolumab (6). Other biomarkers such as chromosome instability, tumor microsatellite instability, and T-cell surface markers such as PD-1^1^, CD38 and CD39^2^, or tumor-infiltrating PD-1^hi^ CD8^+^ T cells (8) might serve as prognostic and predictive biomarkers for ICI therapy (9, 10). However, genetic biomarkers require tumor biopsy samples, which are invasive and limit longitudinal analysis for continuous disease monitoring. Hence, liquid biopsy-based biomarkers are attracting more recent attention (11–15).

Blood cell count test (BCT) is a routine, regularly performed blood test conducted before and during treatment. The less invasiveness of BCT makes it potentially useful for longitudinal disease monitoring, especially at the end of each treatment cycle to decide if the treatment plan needs to be altered. BCT provides a direct overview of the immune landscape based on the counts of various types of immune cells. For instance, high pre-treatment neutrophil-to-lymphocyte ratio (NLR) and platelet-to-lymphocyte ratio (PLR) correlated with poor survival outcomes in NSCLC patients treated with ICIs, regardless of TMB (16–18). However, a limitation of most of the published studies is either small cohorts or analysis of multiple ICI therapies in diverse clinical settings, which may compromise the validity of findings. Consequently, this study focuses on survival data obtained from four international, multicenter clinical trials to conduct *post-hoc* analysis of NSCLC patients undergoing atezolizumab (Ate) single-agent treatment, whereas docetaxel (Dtx) single-agent treatment, the standard protocol for second-line NSCLC treatment at the time of clinical trial, served as control. BCT was conducted at three time points: baseline (T1), 6 weeks on-treatment (T2), and 12 weeks on-treatment (T3). The overarching goal was to identify a BCTscore as a biomarker that may predict overall survival in NSCLC patients treated with atezolizumab.

## Methods

### Study cohort

Pseudonymized individual participant data from the single-arm phase II studies FIR (NCT01846416) (19) and BIRCH (NCT02031458) (20) and the two-arm randomized controlled trials (RCTs) POPLAR phase II study (NCT01903993) (3) and OAK phase III study (NCT02008227) (4) were provided by Genentech Inc. and accessed through the secure Vivli online platform. Docetaxel was used in the POPLAR and OAK studies as chemotherapy control to the anti–PD-L1 immunotherapy atezolizumab. Raw data were extracted and compared with the available published data to ensure accuracy. Secondary analysis of the trial data was deemed to be of negligible risk and was approved by the Institutional Review Board of the Second Affiliated Hospital, Zunyi Medical University (No. YXLL(KY-R)-2021-010). Deidentified data were accessed according to Roche’s policy and process for Vivli. Data analyses were conducted from 2 March 2021 to 30 June 2021.

**Figure 1:**
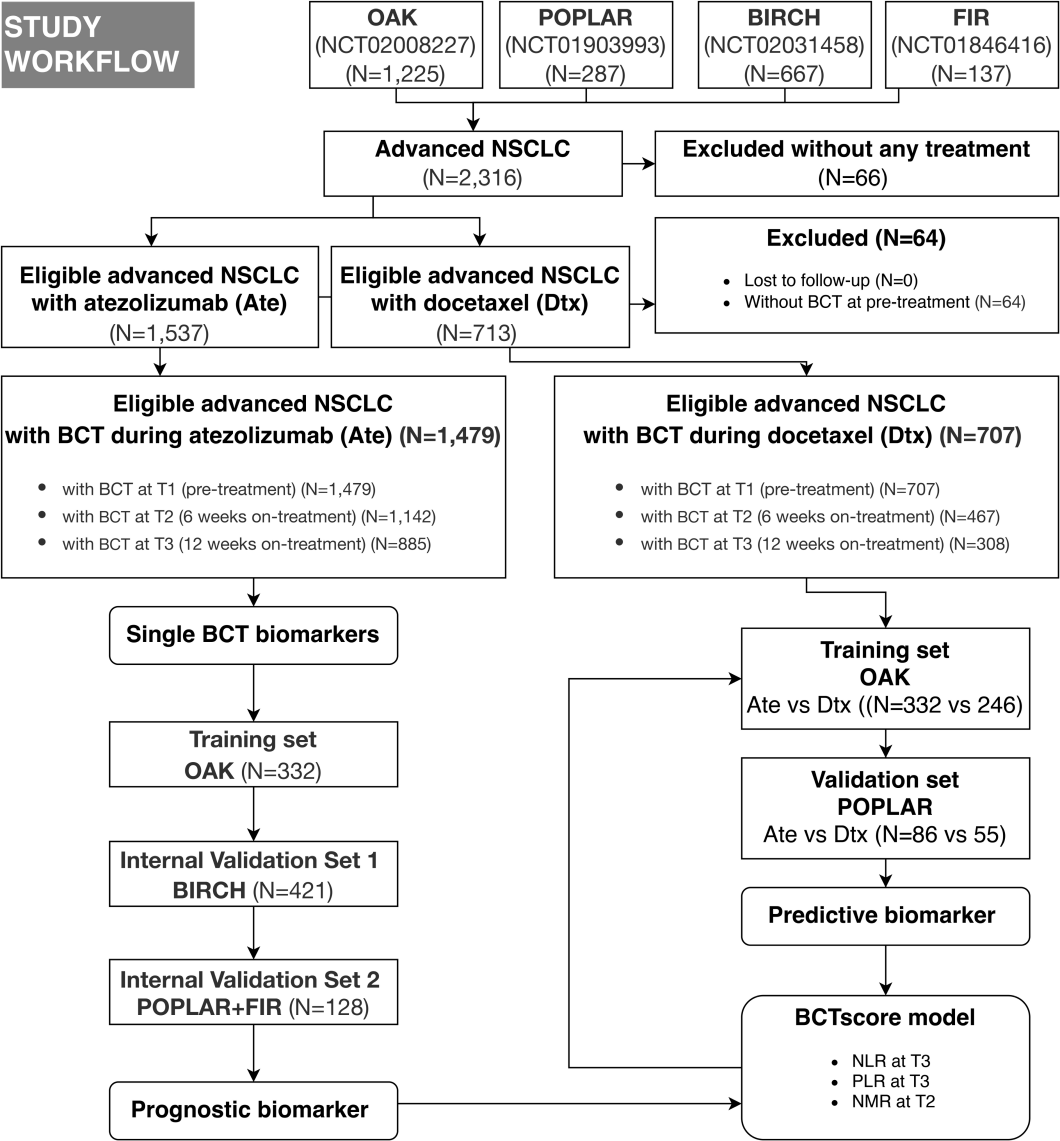
Flow chart demonstrating the patient cohorts of the indicated trials and the approach for the development of a blood cell count test (BCT)–based score (BCTscore). The internal cohorts are from four international, multicenter studies (OAK, POPLAR, BIRCH, and FIR). Ate, atezelizumab; Dtx, docetaxel.

A total of 2,316 patients were included from the four clinical trials, and after the exclusion of untreated patients and patients without pre-treatment BCT, 1,479 and 707 advanced NSCLC patients undergoing atezolizumab and docetaxel treatment, respectively, were included in this study (**Figure 1**). Of note, atezolizumab was administered either as first-line or second-line therapy after failure of prior chemotherapy in the four trials used in this study. Atezolizumab and docetaxel were both administered every 3 weeks in the two-arm RCTs POPLAR and OAK. BCT was obtained at three time points: pre-treatment baseline (T1), 6 weeks on-treatment (T2), and 12 weeks on-treatment (T3). Baseline was defined as within 28 days prior to the start of treatment. Time points T2 and T3 corresponded to the first day of treatment cycles 3 and 5, respectively.

### Predictor and treatment outcome definitions

The definitions of OS, PFS, clinical benefit (CB), and objective response rate (ORR) were detailed in each trial (3, 4, 19, 20). In this study, OS was used as the primary end point, whereas PFS, according to Response Evaluation Criteria in Solid Tumors (RECIST version 1.1), CB, and ORR was used as secondary end points.

All biomarkers acquired from BCT, such as absolute cell counts of red blood cells, white blood cells, platelets, as well as the ratios of immune cell subgroups were subjected to the biomarker screening. The BCT biomarkers of NLR, PLR, NMR, and lymphocyte-to-monocyte ratio (LMR) were calculated by dividing absolute cell counts of corresponding immune cells acquired from BCT. The identified biomarkers were named with the abbreviation of the immune cell ratios followed by the indication of the time points T1, T2, and T3, respectively.

### Statistical analysis

Associations between BCT biomarkers and OS or PFS were calculated by the Cox proportional hazards regression model and reported as the mean of hazard ratio (HR) with two-sided 95% confidence interval (CI) and *p*-value as calculated by the Wald test. The Kaplan–Meier method was used to estimate median OS and PFS between risk groups with a stratified log-rank test at the two-sided significance level. Survival analysis was performed by the *survival* (V.3.2-11) and *survminer* (V.0.4.9) packages. To analyze the degree of discrimination of biomarkers, we performed time-dependent receiver-operating characteristic (ROC) analysis and calculated the area under curve (AUC) for the indicated survival outcomes by the *timeROC* (V.0.3) and *pROC* (V.1.17.0.1) packages. Comparisons of CB, ORR, or clinical factors between the specified groups were calculated by the generalized linear model (GLM) to report relative risk (RR) with 95% CI and *p*-value as calculated by the Pearson’s χ2-test or Fisher’s exact test. Comparisons of BCT biomarkers between the treatment groups or different time points of the same treatment group were performed using Wilcoxon signed-rank test. All statistical analyses were carried out in R V.3.6.1 (R Foundation for Statistical Computing). *P* ≤ 0.050 was considered to be statistically significant. All analyses were univariate except for the multivariate Cox analyses. In multivariate analysis, the BCT biomarker(s) and the clinical factors of sex (male/female), age, race (white/Asian/other), Eastern Cooperative Oncology Group performance status (ECOG PS), metastasis, and pre-treatment PD-L1 (high ≥ 1%/low < 1%; except for the BIRCH study, high ≥ 5%/low < 5%) were included; however, the additional biomarkers of body mass index (BMI) and smoker (never/previous/current) were insignificant as assessed by univariate Cox analysis and were, hence, removed from the analyses.

## Results

### Identification of BCT biomarkers related to treatment outcomes of patients treated with atezolizumab but not of those treated with docetaxel

Initially, the datasets of the four international, multicenter studies containing 1,479 atezolizumab-treated patients’ survival data (the baseline characteristics of these patients are summarized in **Supplementary Table S1**) were combined to identify 80 common BCT biomarkers that demonstrated correlations to PFS and OS in advanced NSCLC based on HR calculation. Next, we removed the 62 BCT biomarkers that we identified from analyses of the survival data of 707 advanced NSCLC patients who underwent docetaxel treatment. Moreover, all biomarkers containing absolute cell counts were eliminated to avoid sampling-based systemic errors. Hence, 11 BCT biomarkers unique to the atezolizumab-treated patient group remained. Consequently, based on existing knowledge of immune biomarkers, we selected the cell ratios of NLR, PLR, and LMR at 12 weeks on-treatment (NLR_T3, PLR_T3, and LMR_T3), and NMR at 6 and 12 weeks on-treatment (NMR_T2 and NMR_T3), for further analysis. Frequency distribution analysis of these four BCT biomarkers at pre-treatment (T1), followed by Wilcoxon signed-rank test of the absolute difference between the mean values of the two patient groups showed no significant difference between the atezolizumab and docetaxel treatment groups in OAK and POPLAR studies (**Supplementary Figure S1**). This suggested that baseline levels of these biomarkers were comparable between the two treatment groups. Hence, we deduced that changes in subsequent time points at T2 and T3 likely occurred after treatment started (**Supplementary Figure S2**).

**Figure 2:**
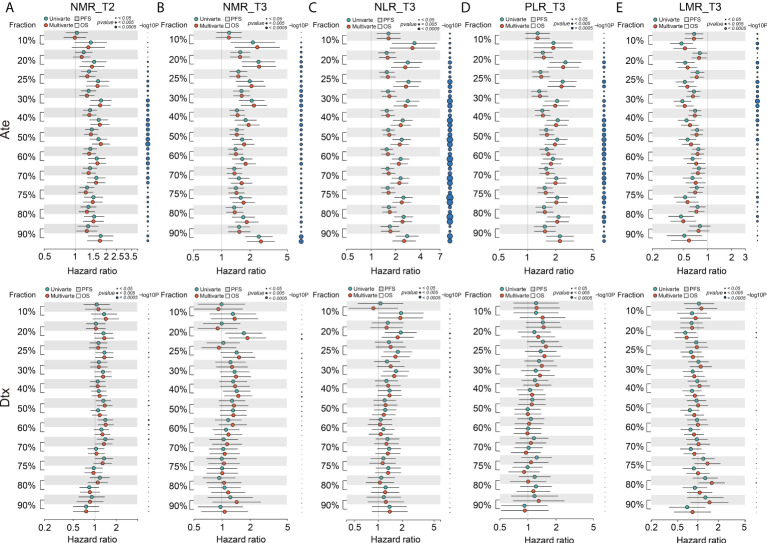
Forest plot of hazard ratio (HR) for overall survival (OS) and progression-free survival (PFS) of the BCT biomarkers **(A)** NMR_T2, **(B)** NMR_T3, **(C)** NLR_T3, **(D)** PLR_T3, and **(E)** LMR_T3 in decile patient fractions in the atezolizumab (Ate) or docetaxel (Dtx) treatment groups of the combined internal cohorts. Mean HRs for OS (white shade) or PFS (gray shade) under univariate (green) or multivariate (red) Cox analysis is indicated by the dots, the range of HR is indicated by the error bar of the forest plot; -log_10_ *p*-value of each calculated HR is indicated by the size of the blue dots adjacent to the forest plot.

Next, we performed univariate Cox analysis by decile patient fractions [Ref: Valero] at 10% intervals from 10% to 90% or by the quadrant percentiles of 25% and 75% for all five biomarkers in the combined datasets of atezolizumab-treated patients to calculate HRs for OS and PFS (**Figure 2**), respectively. NLR_T3 showed significant HR for all patient cutoffs examined in the atezolizumab-treated group and for both OS and PFS. PLR_T3 showed significant PFS HR for the > 10% patient fractions and significant OS HR for all defined patient fractions. LMR_T3 showed significant but inconsistent PFS HR between the 25% and 80% patient fractions and significant OS HR for the > 10% patient fraction. NMR_T2 showed significant PFS HR at the > 20% patient fractions and significant OS HR at the > 10% patient fractions. NMR_T3 showed significant PFS HR at the > 10% patient fractions and significant OS HR for all patient fractions.

On multivariate analysis, we initially screened for clinical factors that might confer to PFS and OS in atezolizumab-treated NSCLC patients (**Supplementary Table S2**). Similar to univariate analysis, we performed multivariate Cox analysis using the same patient fractions in the combined datasets for PFS and OS (**Figure 2**), respectively. NLR_T3, PLR_T3, NMR_T2, and NMR_T3 all depicted identical trends to univariate analysis. Alternatively, LMR_T3 showed significant PFS HR from 10% to 80% patient fractions and significant OS HR from 10% to 50% patient fractions. In contrast, all five BCT biomarkers showed non-significant HRs for both OS and PFS in the docetaxel-treated group (**Figure 2**). Collectively, these results suggested that LMR_T3 exhibited significant but inconsistent HRs as compared with the rest of the selected biomarkers. Furthermore, as deduced from its definition, LMR_T3 displayed HR < 1, whereas the other four biomarkers displayed HR > 1.

After that, we applied the univariate and multivariate Cox analysis with decile patient fractions to the cohort of atezolizumab-treated NSCLC patients in the four individual trials, respectively (**Supplementary Figure S3**). In concordance to the joint analyses, all of the five biomarkers showed no significant HRs for both PFS and OS in the docetaxel treatment group. In contrast, positive results, consistent to the combined cohort, were obtained for all biomarkers in the BIRCH and OAK cohorts for both PFS and OS. This was also true of the POPLAR cohort, except for LMR_T3. However, in the FIR cohort none of the biomarkers demonstrated significant HRs for either PFS or OS, but this is most likely because of the small sample size (21). Consequently, absolute integer cutoff values were set for the combined cohort using the patient fractions of 25–50% for all five biomarkers to establish a BCTscore model. The application of these variables to univariate and multivariate Cox analysis of each trial’s cohort succeeded in narrowing the range of each biomarker’s integer cutoff values to uncover the significant range (**Supplementary Figure S4**). NLR_T3, PLR_T3, and NMR_T2 confirmed consistently significant PFS and OS HRs in the cohorts of BIRCH, OAK, and POPLAR. In contrast, all cutoff values of LMR_T3 did not. Because LMR_T3 showed consistently poor prognostic power in all of the above outlined analyses, it was removed from the biomarker selection for the BCTscore.

Hence, all combinations of the three selected BCT biomarkers, namely, NLR_T3, PLR_T3, and NMR_T2, formed the 16 BCTscore candidates subjected to further optimization for clinical application (**Supplementary Table S3**).

### Optimization of BCT biomarker combinations to establish the BCTscore model

To establish the BCTscore model, the OAK study was used as our training cohort. Next, the BIRCH study was used as internal validation cohort 1, and the POPLAR combined with the FIR study as internal validation cohort 2. Univariate and multivariate Cox analysis demonstrated that all of the 16 BCTscore candidates demonstrated significant HRs in both OS and PFS, as well as RR for CB and ORR (**Supplementary Figure S5**). To further narrow down the BCTscore candidates, we performed ROC analysis for OS, PFS, CB, and ORR. The BCTscore candidate 2 (BCTscore #2) was the only candidate that had good AUC for OS, PFS, CB, and ORR in all of the three internal cohorts (**Supplementary Table S4**). Hence, BCTscore candidate 2, composing of the BCT biomarkers of NLR and PLR at 12 weeks on-treatment (T3) and NMR at 6 weeks on-treatment (T2) with absolute cutoff values of NLR_T3 = 5, PLR_T3 = 180, and NMR_T2 = 6, respectively, was selected as the BCTscore model for NSCLC.

**Figure 3:**
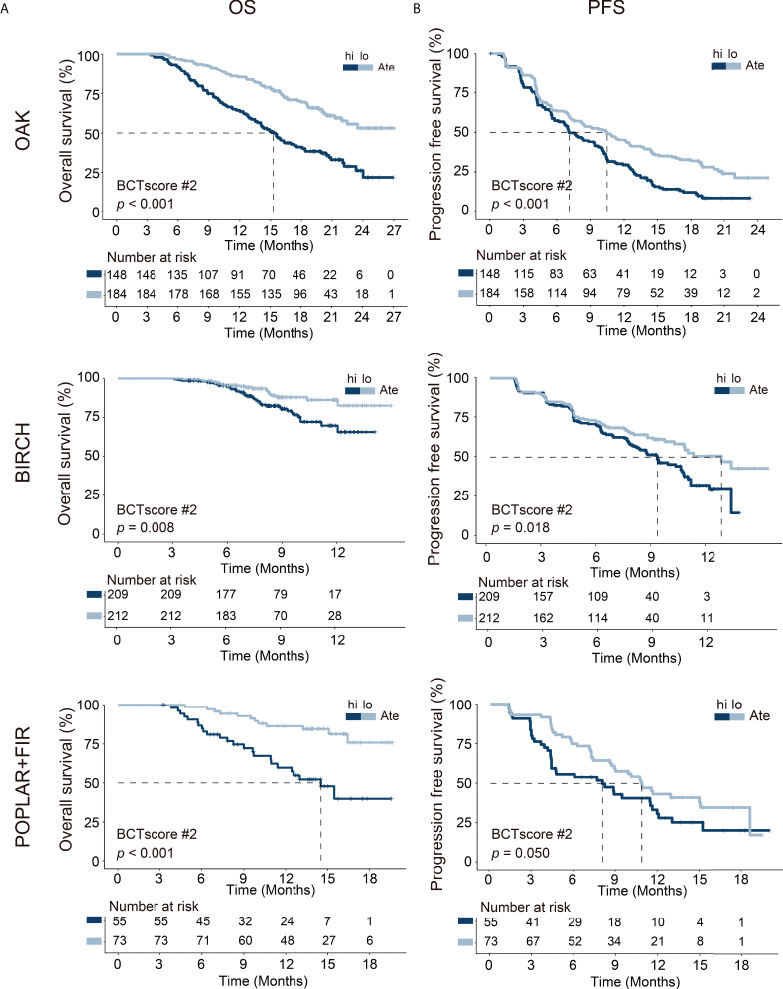
Kaplan–Meier analysis of **(A)** overall survival (OS) and **(B)** progression-free survival (PFS) between high-risk (hi) and low-risk (lo) patients, as defined with the identified BCTscore candidate 2 (BCTscore #2), treated with atezolizumab (Ate) of the training cohort (OAK) and the internal validation cohorts (BIRCH and POPLAR + FIR). The percentage of survival of high-risk (dark blue) and low-risk (light blue) patients is plotted against the time in months.

**Figure 4:**
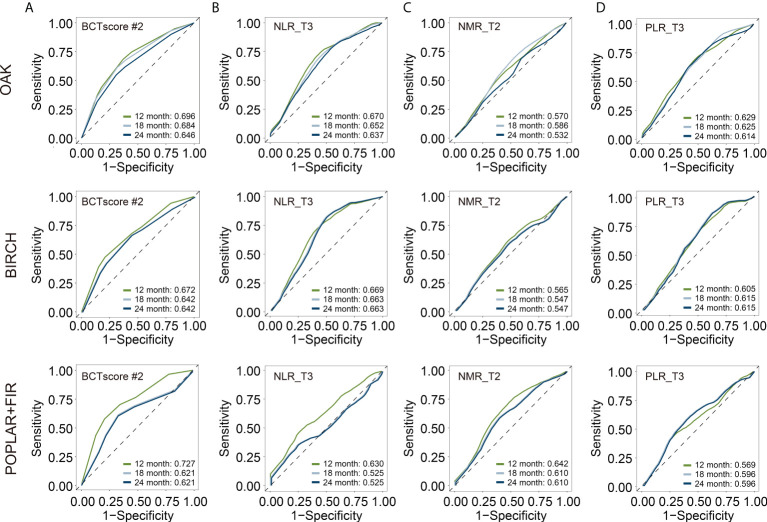
Time-dependent receiver-operating characteristic (ROC) analysis for overall survival (OS) to obtain the area under curve (AUC) of **(A)** BCTscore candidate 2 (BCTscore #2) and the BCT biomarkers **(B)** NLR_T3, **(C)** NMR_T2, and **(D)** PLR_T3 of the atezolizumab-treated patients of the training cohort (OAK) and the internal validation cohorts (BIRCH and POPLAR + FIR). Sensitivity is plotted against specificity.

This BCTscore model displayed significant OS and PFS HRs in both univariate and multivariate Cox analysis in all of the three cohorts (**Supplementary Figure S5A**). The OAK cohort’s RR for CB (univariate = 0.60 [95% CI: 0.39–0.93], *P* = 0.024; multivariate = 0.56 [95% CI: 0.35–0.88], *P* = 0.014) (**Supplementary Figure S5B**) and ORR (univariate = 0.53 [95% CI: 0.31–0.91], *P* = 0.22; multivariate = 0.58 [95% CI: 0.37–0.88], *P* = 0.013) with BCTscore stratification (**Supplementary Figure S5B**) were good. The rate of CB (high risk = 38%, low risk = 51%) and ORR (high risk = 17%, low risk = 28%) of the low-risk atezolizumab-treated patients in the OAK cohort after BCTscore stratification (**Supplementary Table S5**) were also higher than the 48% CB and 14% ORR reported in the original study (4). Furthermore, survival analysis also showed that our newly identified BCTscore model presented significant difference in both OS and PFS between high- and low-risk patients in the atezolizumab-treated group (**Figure 3**). ROC analysis resulted in a BCTscore model that consistently exhibited better OS AUC in the OAK (AUC_12month_ = 0.696), BIRCH (AUC_12month_ = 0.672), and POPLAR+FIR studies (AUC_12month_ = 0.727) than that of each of the three single BCT biomarkers in these studies (**Figure 4**). However, the AUCs of the BCTscore model were lower than those of NLR_T3 for PFS (**Supplementary Figure S6**), CB (**Supplementary Figure S7**), and ORR (**Supplementary Figure S7**) in the OAK cohort, whereas the BCTscore model depicted better AUCs than the standalone BCT biomarkers for these survival indicators in the BIRCH and POPLAR+FIR cohorts.

### Investigation of the BCTscore model as a predictive biomarker

Last, in order to test whether the newly identified BCTscore model could serve as a predictive biomarker, we performed survival analysis on the OAK and POPLAR RCTs. The Ate *versus* Dtx HRs of each BCT biomarker’s absolute cutoff value or decile fractionated BCTscore candidates above and below the cutoff were also calculated to determine whether each BCTscore candidate was a predictive biomarker (22). Results showed that NLR_T3 presented significant PFS prognosis in the OAK study and may be prognostic of OS in the POPLAR study, whereas PLR_T3 and NMR_T2 achieved no significant results in both RCTs (**Supplementary Table S6**). On the other hand, all the 16 BCTscore candidates had some predictive power in > 75% fractions in the OAK and the POPLAR RCTs (**Supplementary Table S7**).

**Figure 5:**
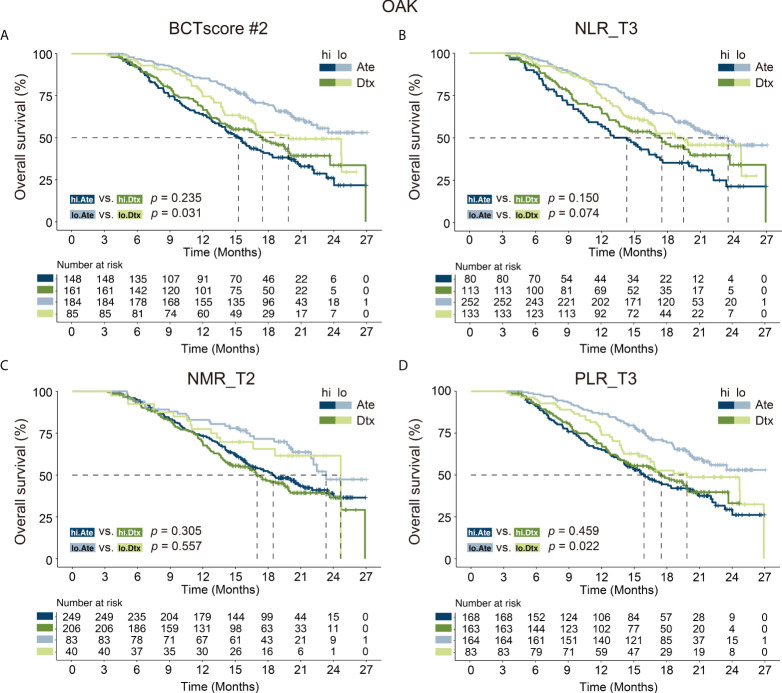
Kaplan–Meier analysis of overall survival (OS) for **(A)** BCTscore candidate 2 (BCTscore #2) and the BCT biomarkers **(B)** NLR_T3, **(C)** NMR_T2, and **(D)** PLR_T3 comparing atezolizumab (Ate)–treated patients (dark blue) against docetaxel (Dtx)–treated patients (dark green) in the high-risk (hi) group, and comparing between Ate-treated patients (light blue) against Dtx-treated patients (light green) in the low-risk (lo) group of the training cohort (OAK).

In this regard, our newly developed BCTscore model is a strong predictive model specific to atezolizumab-treated NSCLC patients in comparison to docetaxel-treated patients for OS in the cohorts of OAK (BCTscore low risk: HR _Ate_ vs. _Dtx_ = 1.54 [95% CI: 1.04–2.27], *P* = 0.031; high risk: HR _Ate_ vs. _Dtx_ = 0.84 [95% CI: 0.62–1.12], *P* = 0.235) (**Figure 5**) and POPLAR (BCTscore low risk: HR _Ate_ vs_. Dtx_ = 2.93 [95% CI: 1.21–7.10], *P* = 0.013; high risk: HR _Ate_ vs_. Dtx_ = 0.56 (95% CI: 0.29-1.07), *P* = 0.074) (**Supplementary Figure S8**).

In contrast, no significant difference was observed in PFS between the atezolizumab and docetaxel treatment groups in both the OAK (BCTscore low risk: HR _Ate_ vs_. Dtx_ = 1.22 [95% CI: 0.85–1.75], *P* = 0.267; high risk: HR _Ate_ vs_. Dtx_ = 0.79 [95% CI: 0.60–1.04], *P* = 0.092) (**Supplementary Figure S9**) and POPLAR studies (BCTscore low risk: HR _Ate_ vs_. Dtx_ = 1.06 [95% CI: 0.50–2.24], *P* = 0.877; high risk: HR _Ate_ vs_. Dtx_ = 0.87 (95% CI: 0.47–1.59), *P* = 0.652) (**Supplementary Figure S10**), in consistence to the findings of the two studies (3, 4). Similarly, analysis of the relative response rate suggested that our BCTscore model did not distinguish between the atezolizumab- and docetaxel-treated patients in both CB (OAK high risk: Ate *versus* Dtx = 1.25, low risk: Ate *versus* Dtx = 0.85; POPLAR high risk: Ate *versus* Dtx = 0.67, low risk: Ate *versus* Dtx = 0.95) and ORR (OAK high risk: Ate *versus* Dtx = 0.96, low risk: Ate *versus* Dtx = 0.95; POPLAR high risk: Ate *versus* Dtx = 0.79, low risk: Ate *versus* Dtx = 0.78) (**Supplementary Table S8**), reinforcing the fact that our newly defined BCTscore model is a predictive and prognostic biomarker particularly for OS.

Taken together, these results indicate that our BCTscore model can predict the overall survival of NSCLC patients treated with anti–PD-L1 atezolizumab therapy at 12 weeks on-treatment to decide whether treatment can be terminated or alternative treatment plans should be devised.

## Discussion

In spite of the success of ICI therapy in NSCLC treatment, robust prediction of treatment response remains one of the biggest challenges (23). BCT, which is a routine clinical procedure, provides an unbiased overview of the immune landscape for patient stratification and longitudinal ICI efficacy assessment without the need for specialized analysis. This study showed that the BCTscore model serves as both a strong prognostic and predictive biomarkers of ICI efficacy, especially the prediction of overall survival beyond the date of the BCT test. The strengths of this study are manyfold. First, the OS AUCs of our newly identified BCTscore model surpassed that of PD-L1 (24) and TMB (25, 26), both of which can only be applied by more invasive tissue biopsy procedures. Hence, our BCTscore model complements PD-L1 and TMB at later stages to predict survival beyond the initial treatment cycle. Next, an important feature of our analyses is easily obtainable longitudinal data. We presume that the immune landscape alters during treatment, so each time point signifies a discrete event (27). Simultaneously, we removed any BCT biomarker at any time point that is correlated to patient survival after docetaxel treatment during initial biomarker screening, thus selecting atezolizumab-specific BCT biomarkers. Our assumption is that BCT biomarkers typical to cancer prognosis regardless of biological mechanism will show significant HR in the docetaxel patient group and, hence, should be removed from subsequent analyses. Hence, prognostic biomarkers were ruled out using our approach.

Furthermore, delays in immune response have been frequently observed during ICI therapy (28, 29). That is why later time points are hypothesized to have better indicative power as compared with earlier time points. Nevertheless, the biological nature of the BCT biomarker still holds the key to a successful predictive biomarker. For example, both NMR_T2 and NMR_T3 depicted significant HRs in our preliminary analysis. However, NMR_T2 showed better results in the survival analysis of the OAK and POPLAR studies than NMR_T3, whereas NLR_T3 and PLR_T3 displayed good results in the same analysis. This result demonstrated that after the number of neutrophils increased at 6 weeks on-treatment (T2), subsequent increase had little impact on disease prognosis and prediction; instead, the reduction of lymphocytes and increase in platelet numbers at 12 weeks on-treatment (T3) come into play. No prior studies have examined this, to our understanding, in the context of ICI, and the biological mechanism on the temporal changes of the immune landscape during ICI treatment remains elusive.

Additionally, we picked immune cell ratios over absolute cell numbers to avoid systemic errors during blood sampling and sample analysis performed in different medical centers. The cell ratios used for our analysis were selected based on cell lineage and published data. For example, NMR and NLR were picked to distinguish changes in the neutrophil population against the immune cell lineages of monocytes and lymphocytes. It is known that lymphocytes are directly involved in tumor killing, whereas monocytes have more diverse biological roles. Indeed, the fact that NLR outperforms all other cell ratios as a single BCT biomarker reinforces previous observations that increasing neutrophil numbers and decreasing lymphocyte numbers result in poor cancer prognosis (30, 31). Alternatively, the poor correlation between LMR and survival supports the hypothesis that neutrophils, but not the entire monocyte population, contribute to cancer prognosis. Next, the improvement in prognostic ability by combining multiple immune cell ratios suggests that many factors play a role in ICI treatment; for instance, neutrophils were shown to promote tumor metastases (32, 33); platelets present antigens to trigger immune evasion (34). We deduce that future immunological studies will deepen our knowledge of the correlation between the immune landscape and ICI treatment success to unravel more effective and accurate biomarkers (35).

Finally, analysis of the four international, multicenter clinical trials consisting of 1,479 NSCLC patients treated with atezolizumab provides strong statistical evidence to support our findings. We observed statistically significant associations for OS, PFS, ORR, and CB with powerful diagnostic abilities, suggesting that the newly defined BCTscore has prognostic and predictive value in the context of anti–PD-L1 atezolizumab therapy. Nevertheless, because the mechanistic role of atezolizumab is restricted to tumor recognition by T cells, the application of our model to other ICI therapies, such as anti–PD-1 and anti–CTLA-4 therapies, remains to be tested.

A limitation of our study is the lack of TMB measurements in our dataset that would enable direct comparison between the predictive power of our newly defined BCTscore model and TMB. We were restricted in the validation of our results because of the shortage of patients treated by atezolizumab alone. Future studies will focus on the optimization of the absolute cutoff values of each BCT biomarker for different ICI therapies as well as combination therapies.

In summary, we demonstrated, for the first time, *via* a *post-hoc* analysis of four clinical trials, the predictive value of longitudinal blood cell count ratio for NSCLC patients treated with atezolizumab. Together, this study proved that the BCTscore combination of NLR at 12 weeks, PLR at 12 weeks, and NMR at 6 weeks provides prognostic and predictive information without the need to re-biopsy patients undergoing anti–PD-L1 atezolizumab monotherapy. Future studies utilizing our BCTscore model may demonstrate its broader versatility as a prognostic and predictive biomarker in all lung cancer patients undergoing atezolizumab treatment.

## Data availability statement

Data are available upon reasonable request from lead investigator: J-GZ, jianguo.zhou@yahoo.com.

## Ethics statement

The studies involving human participants were reviewed and approved by Zunyi Medical University. Written informed consent for participation was not required for this study in accordance with the national legislation and the institutional requirements. Written informed consent was not obtained from the individual(s) for the publication of any potentially identifiable images or data included in this article.

## Author contributions

Conceptualization, J-GZ, AHW, HM, UG, S-HJ, and DS. Methodology, J-GZ, AHW, and FT. Validation, Y-ZC, BS, RF, MH, HM, UG, and J-GZ. Data curation, J-GZ, AHW, GS, S-SH, HM, SC, and UG. Writing—original draft preparation, AHW, HW, J-GZ, and HW. Writing—review and editing, UG, HM, BF, SC, DS, and MH. Visualization, J-GZ and HW. Supervision, HM, UG, J-GZ, and DS. Project administration, J-GZ, HM, UG, and RW. Funding acquisition, J-GZ, S-HJ, and AHW. All authors contributed to the article and approved the submitted version.

## Funding

This research was funded by the National Natural Science Foundation of China (Grant No. 82060475), the National Natural Science Foundation of Guizhou Province (Grant Nos. [2020]1Z062, ZK2021-YB435, and ZK2022-YB632), Lian Yun Gang Shi Hui Lan Public Foundation (Grant No. HL-HS2020-92). AW Medical Company Limited received startup funding from the University Development Fund of University of Macau.

## Acknowledgments

We would like to thank all of the patients, investigators, and staff involved in the FIR, BIRCH, POPLAR, and OAK studies who released and shared their data. This publication is based on research using data from data contributors, Roche, that has been made available through Vivli, Inc. (Data Request ID: 5935; Lead Investigator: J-GZ). Vivli has not contributed to or approved, and is not in any way responsible for, the contents of this publication. The present work was performed by J-GZ in partial fulfillment of the requirements for containing the degree “Dr. rer. biol. hum.”

## Conflict of interest

MH and UG reports collaborations with Merck Serono (advisory role, speakers’ bureau, honoraria, travel expenses, and research funding), MSD (advisory role, speakers’ bureau, honoraria, travel expenses, and research funding), AstraZeneca (research funding), Novartis (research funding), BMS (advisory role, honoraria, speakers’ bureau), Teva (travel expenses). AHW is a founder and shareholder of AW Medical Co. Ltd.

The remaining authors declare that the research was conducted in the absence of any commercial or financial relationships that could be construed as a potential conflict of interest.

## Publisher’s Note

All claims expressed in this article are solely those of the authors and do not necessarily represent those of their affiliated organizations, or those of the publisher, the editors and the reviewers. Any product that may be evaluated in this article, or claim that may be made by its manufacturer, is not guaranteed or endorsed by the publisher.

## Footnotes

1 PD-1, programmed cell death protein 1

2 CD38 and CD39, clusters of differentiation 38 and 39
