# Supplementary material for: Definition of a new blood cell count score for early survival prediction for non-small cell lung cancer patients treated with atezolizumab: Integrated analysis of four multicenter clinical trials
Source: Front Immunol. 2022 Sep 2;13:961926. doi: 10.3389/fimmu.2022.961926 (PMC9478919; doi:10.3389/fimmu.2022.961926)
Supplement: Supplementary file 1 [file DataSheet_1.pdf]

# Definition of a new blood cell count (BCT) score for early survival prediction for non-small cell lung cancer patients treated with atezolizumab: Integrated analysis of 4 multicenter clinical trials

**Jian-Guo Zhou[1,2,3,4*], Ada Hang-Heng Wong[5*], Haitao Wang[6*], Su-Han Jin[7], Fangya Tan[8], Yu-Zhong Chen[9], Si-Si He[1], Gang Shen[1], Benjamin Frey[2,3,4], Rainer Fietkau[3,4], Markus Hecht[3,4], Shamus R. Carr[6], Ruihong Wang[6], Bo Shen[9], David S Schrump[6#], Hu Ma[1#] and Udo S. Gaipl[2,3,4 #]**

## Supplementary Figures

**Supp Figure 1** . . . . . . . . . . 2
**Supp Figure 2** . . . . . . . . . . 3
**Supp Figure 3** . . . . . . . . . . 5
**Supp Figure** 4 . . . . . . . . . . 6
**Supp Figure 5** . . . . . . . . . . 8
**Supp Figure** 6 . . . . . . . . . . 9
**Supp Figure 7** . . . . . . . . . . 10
**Supp Figure 8** . . . . . . . . . . 11
**Supp Figure 9** . . . . . . . . . . 12
**Supp Figure 10** . . . . . . . . . . 13

## Supplementary Tables

**Supp Table 1.**. . . . . . . . . . 14
**Supp Table 2.**. . . . . . . . . . 16
**Supp Table 3.**. . . . . . . . . . 19
**Supp Table 4.**. . . . . . . . . . 20
**Supp Table 5.**. . . . . . . . . . 39
**Supp Table 6.**. . . . . . . . . . 44
**Supp Table 7.**. . . . . . . . . . 68
**Supp Table 8.**. . . . . . . . . . 76

**Supplementary Figure S1.** Density plots of the ratio distribution of the BCT biomarkers (**A**) NLR, (**B**) PLR, (**C**) NMR and (**D**) LMR at baseline (T1) in the atezolizumab (Ate) and docetaxel (Dtx) treatment groups of the internal randomized controlled trials (OAK+POPLAR), accompanied by and (**E**) the comparison chart and *p*-values calculated by the Wilcoxon signed-rank test. Ratio density is plotted against the absolute ratio (except $\log_{10}$ ratio for PLR).

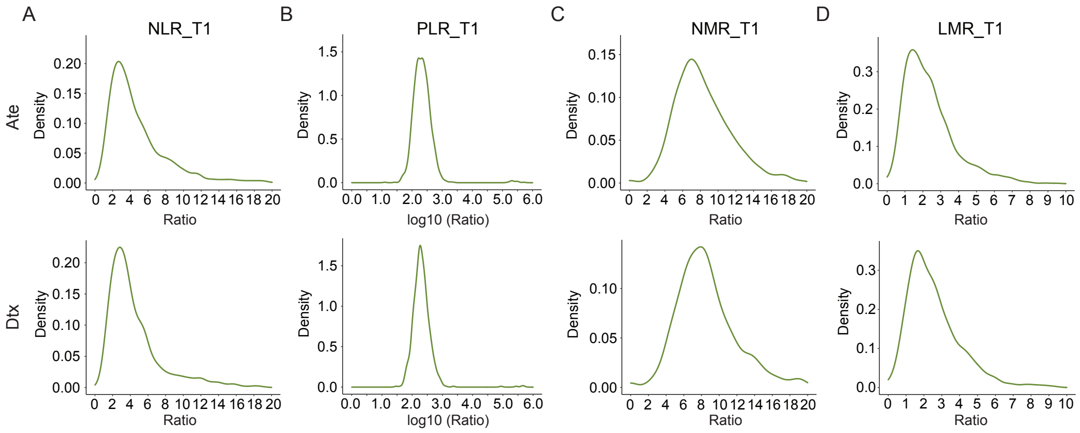


E

| BCT Biomarker | Mean Ratio: Ate | Mean Ratio: Dtx | Width | *p*-value |
|---|---|---|---|---|
| NLR_T1 | 5 | 5 | 270603 | 0.363 |
| PLR_T1 | 1441 | 2054 | 269252 | 0.488 |
| NMR_T1 | 24 | 10 | 250292 | 0.334 |
| LMR_T1 | 5 | 3 | 246724 | 0.143 |

**Supp Figure 1**

**Supplementary Figure S2.** Density plots of the ratio distribution of the BCT biomarkers (**A**) NLR, (**B**) PLR, (**C**) NMR and (**D**) LMR at baseline (T1), 6 weeks (T2) and 12 weeks (T3) on-treatment in the atezolizumab (Ate) and docetaxel (Dtx) treatment groups of the combined internal cohorts. Ratio density is plotted against the absolute ratio (except $\log_{10}$ ratio for PLR).

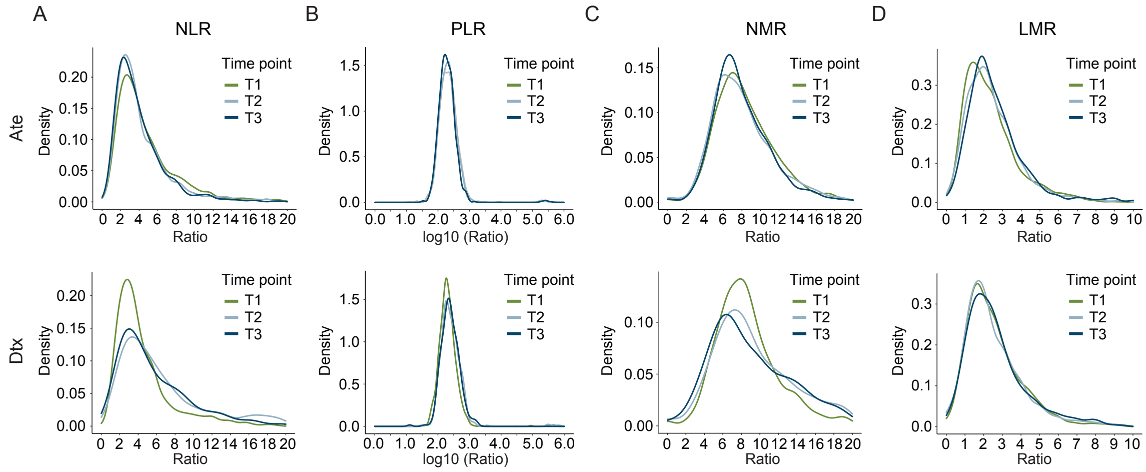


**Supp Figure 2**

**Supplementary Figure S3.** Forest plot of hazard ratio (HR) for overall survival (OS) and progression-free survival (PFS) of the BCT biomarkers (**A**) NMR_T2, (**B**) NMR_T3, (**C**) NLR_T3, (**D**) PLR_T3 and (**E**) LMR_T3 in decile patient fractions in the atezolizumab-treated patients of each internal cohort (OAK, BIRCH, POPLAR, FIR). Mean HRs for OS (white shade) or PFS (grey shade) under univariate (green) or multivariate (red) Cox analysis is indicated by the dots, the range of HR is indicated by the error bar of the forest plot; $-\log_{10}$ *p*-value of each calculated HR is indicated by the size of the blue dots adjacent to the forest plot.

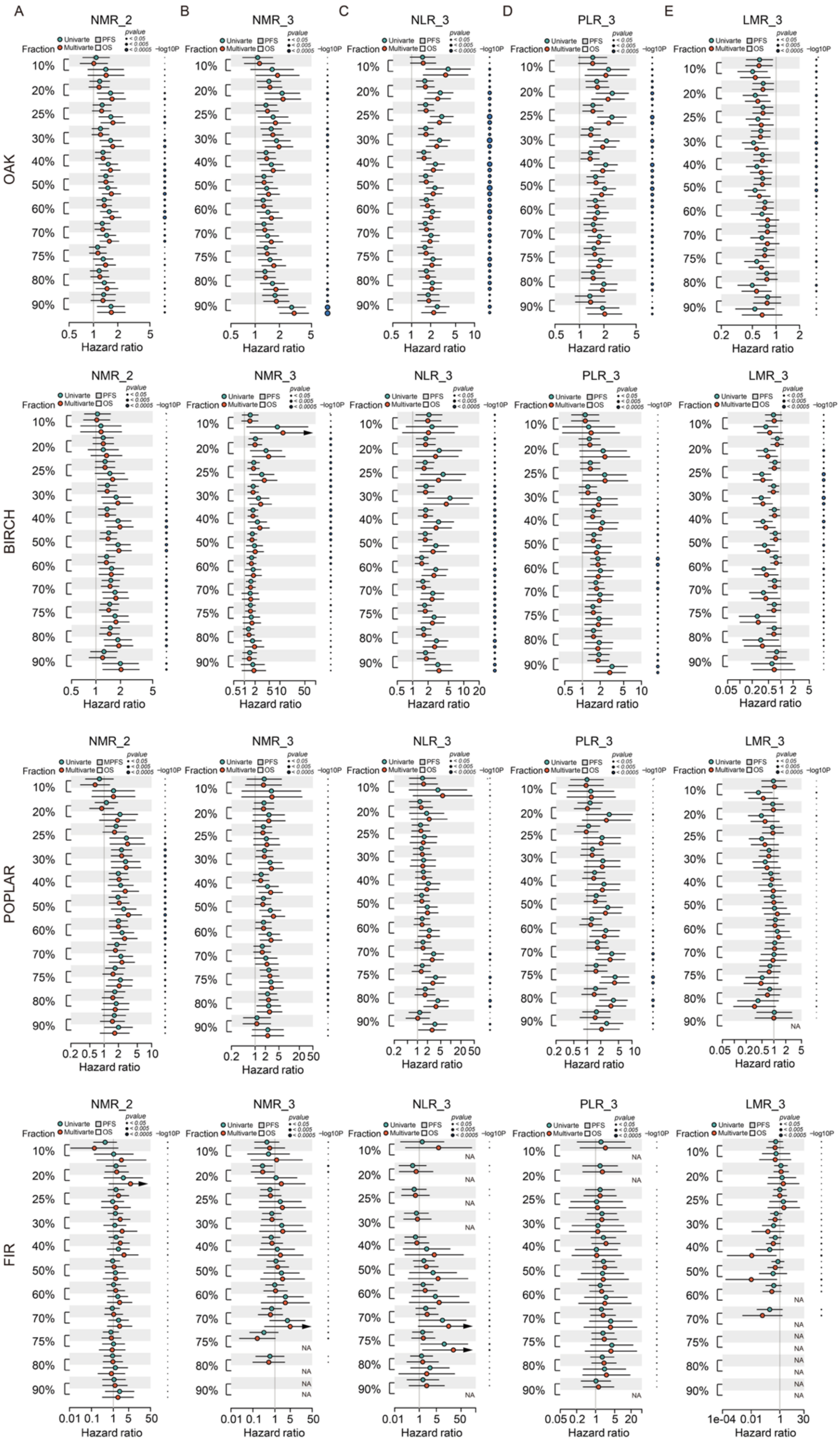


**Supp Figure 3**

**Supplementary Figure S4.** Forest plot of hazard ratio (HR) for overall survival (OS) and progression-free survival (PFS) of the BCT biomarkers (**A**) NMR_T2, (**B**) NMR_T3, (**C**) NLR_T3, (**D**) PLR_T3 and (**E**) LMR_T3 at different absolute cutoff values in the atezolizumab (Ate) or docetaxel (Dtx) treatment groups of the combined internal cohorts. Mean HRs for OS (white shade) or PFS (grey shade) under univariate (green) or multivariate (red) Cox analysis is indicated by the dots, the range of HR is indicated by the error bar of the forest plot; -$\log_{10}$ *p*-value of each calculated HR is indicated by the size of the blue dots adjacent to the forest plot.

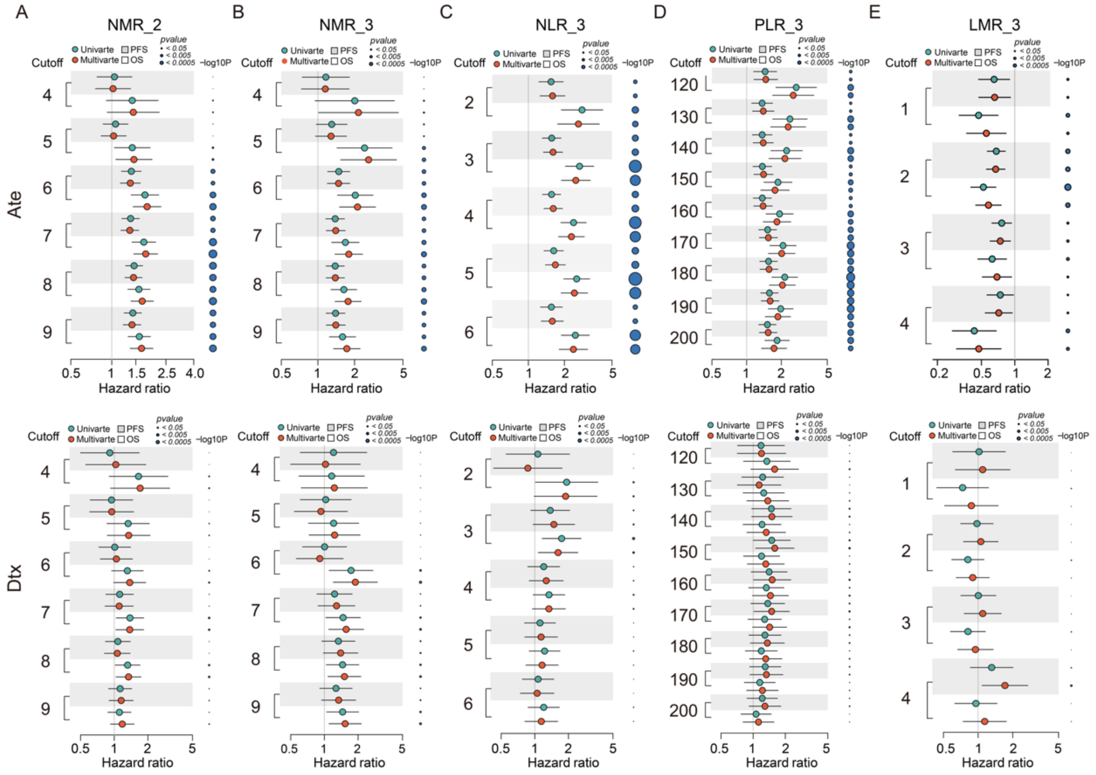


**Supp Figure** 4

**Supplementary Figure S5.** Forest plot of (**A**) hazard ratio (HR) for overall survival (OS) and progression-free survival (PFS) and (**B**) relative risk (RR) for clinical benefit (CB) and objective response rate (ORR) of the BCTscore candidates in the atezolizumab-treated patients of the training cohort (OAK) and the internal validation cohorts (BIRCH and POPLAR+FIR). Mean HRs for OS (white shade) or PFS (grey shade) or RRs for CB (white shade) or ORR (grey shade) under univariate (green) or multivariate (red) Cox analysis is indicated by the dots, the range of HR or RR is indicated by the error bar of the forest plot; $-\log_{10}$ *p*-value of each calculated HR or RR is indicated by the size of the blue dots adjacent to the forest plot.

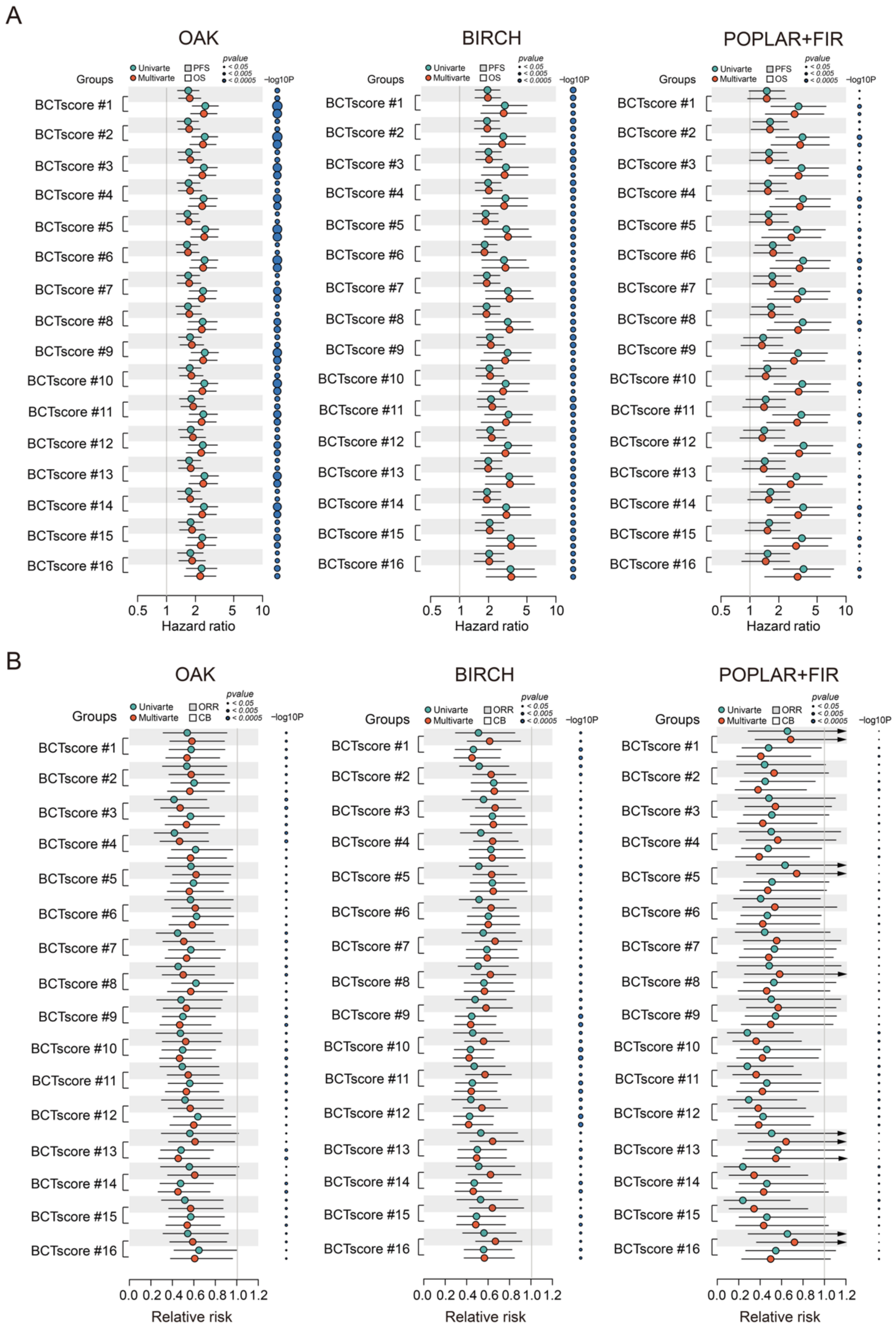


**Supp Figure 5**

**Supplementary Figure S6.** Time-dependent receiver-operating characteristic (ROC) analysis for progression-free survival (PFS) to obtain the area under curve (AUC) of (**A**) BCTscore candidate 2 (BCTscore #2) and the BCT biomarkers (**B**) NLR_T3, (**C**) NMR_T2 and (**D**) PLR_T3 of the atezolizumab-treated patients of the training cohort (OAK) and the internal validation cohorts (BIRCH and POPLAR+FIR). Sensitivity is plotted against specificity.

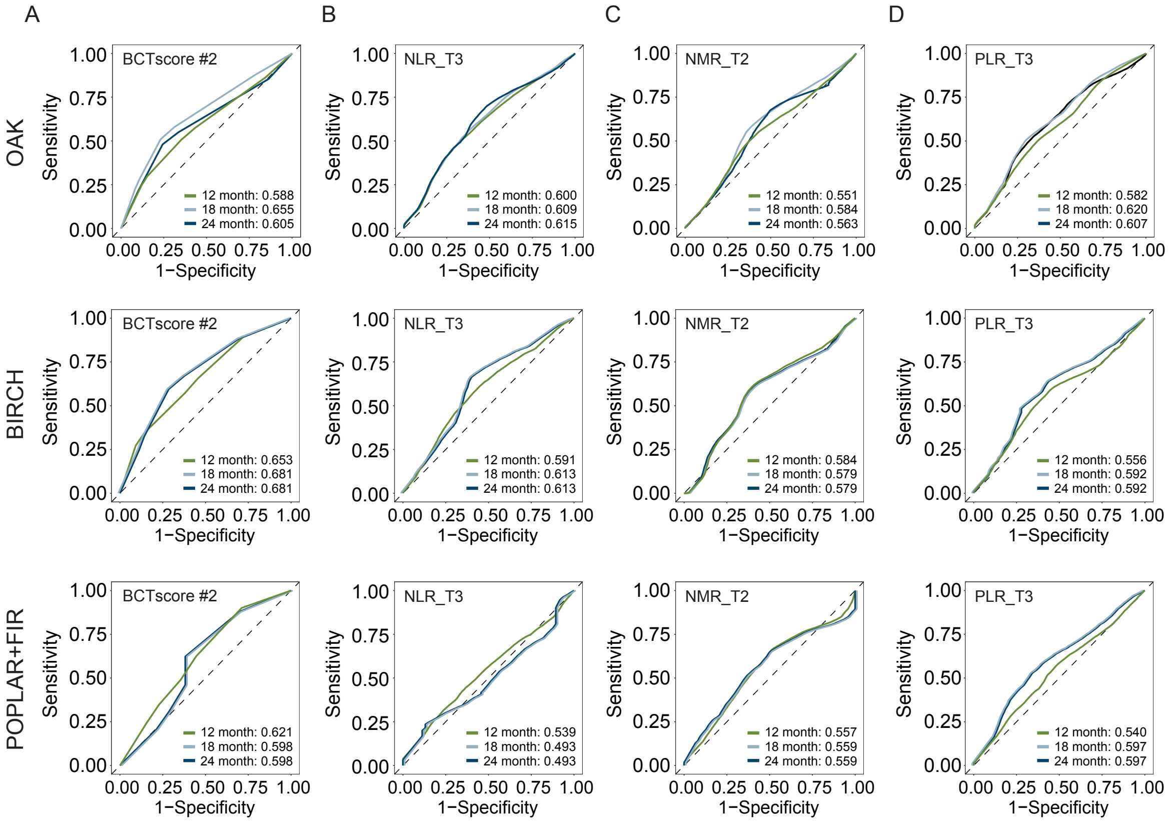


**Supp Figure** 6

**Supplementary Figure S7.** Receiver-operating characteristic (ROC) analysis for (**A**) clinical benefit (CB) and (**B**) objective response rate (ORR) to obtain the area under curve (AUC) of BCTscore candidate 2 (BCTscore #2), compared to the AUCs of the BCT biomarkers NLR_T3, NMR_T2 and PLR_T3 of the atezolizumab-treated patients of the training cohort (OAK) and the internal validation cohorts (BIRCH and POPLAR+FIR). Sensitivity is plotted against specificity.

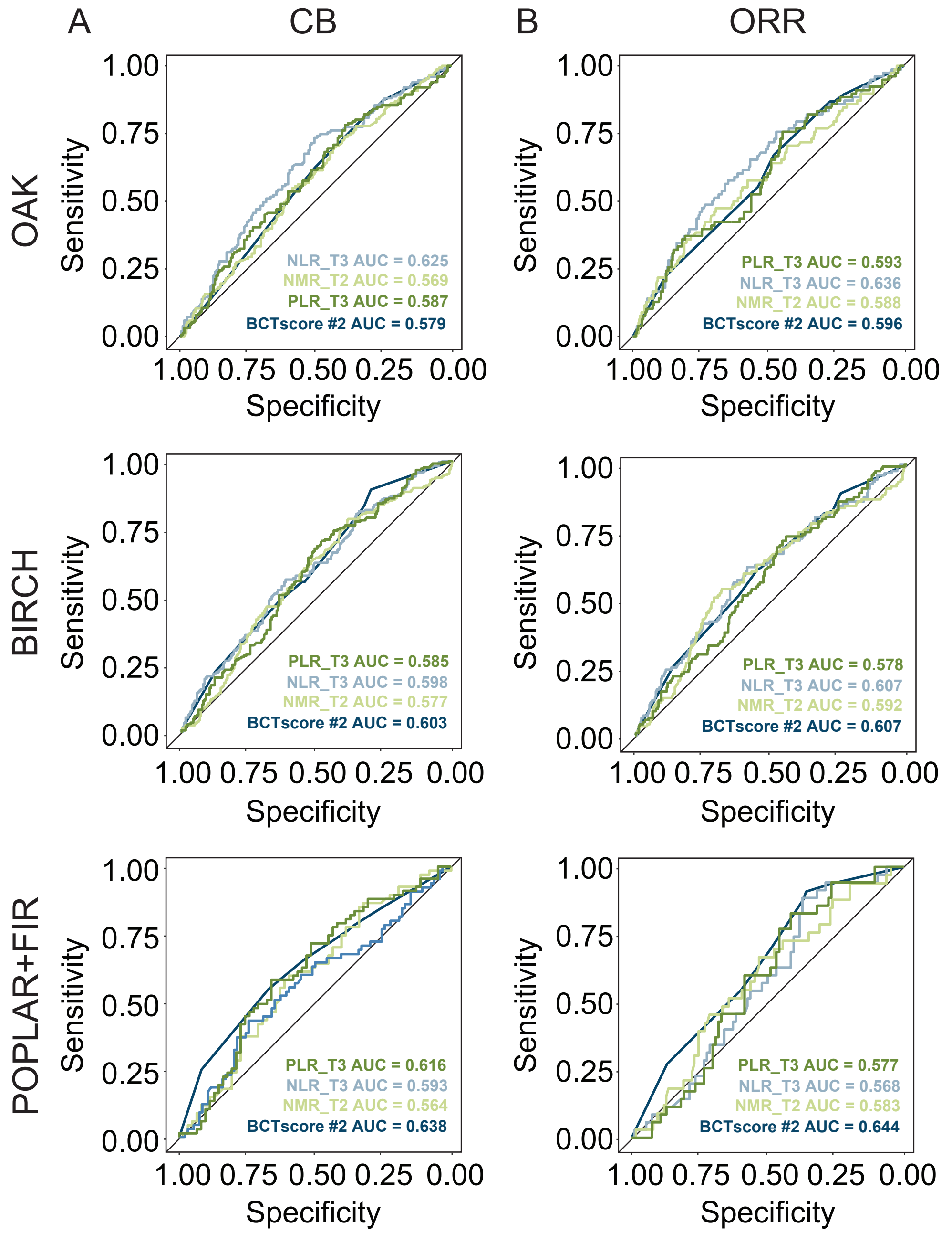


**Supp Figure 7**

**Supplementary Figure S8.** Kaplan-Meier analysis of overall survival (OS) for (**A**) BCTscore candidate 2 (BCTscore #2) and the BCT biomarkers (**B**) NLR_T3, (**C**) NMR_T2 and (**D**) PLR_T3 comparing between atezolizumab (Ate)-treated patients (dark blue) against docetaxel (Dtx)-treated patients (dark green) in the high-risk (hi) group, and comparing between Ate-treated patients (light blue) against Dtx-treated patients (light green) in the low-risk (lo) group of the internal validation cohort (POPLAR).

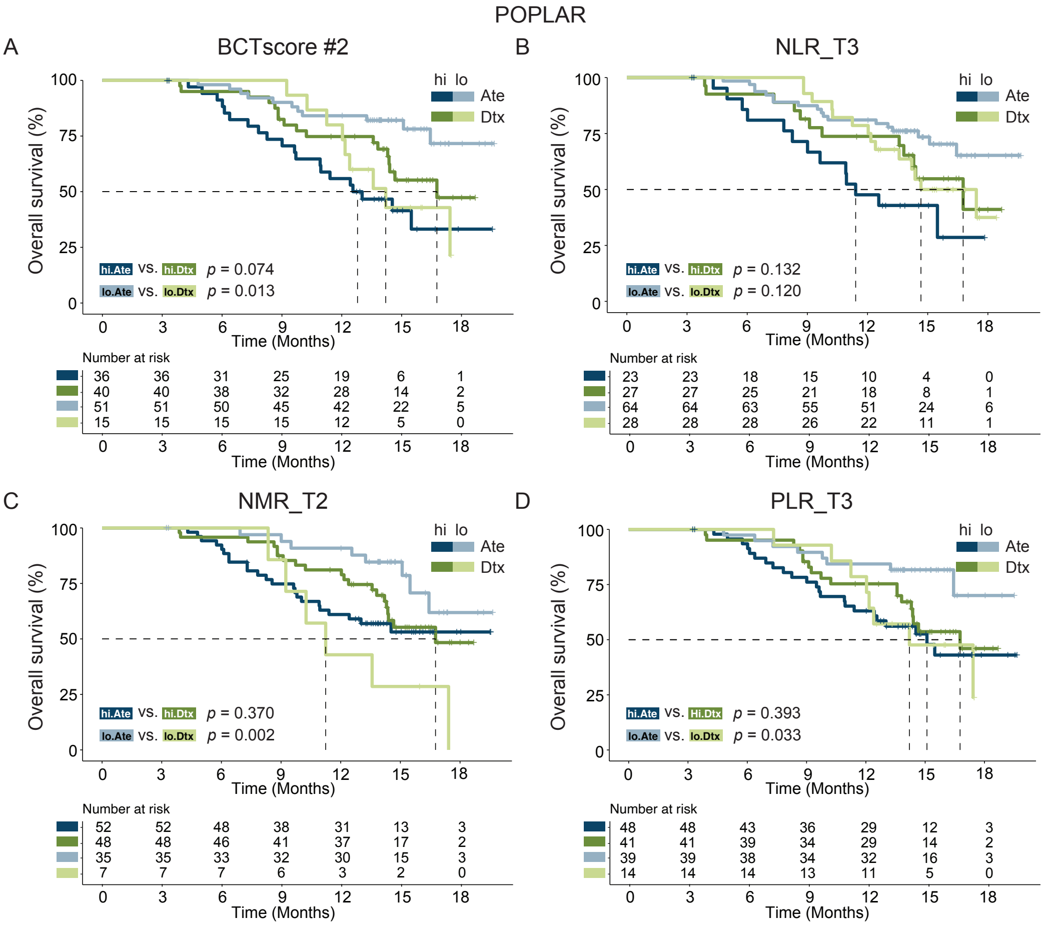


**Supp Figure 8**

**Supplementary Figure S9.** Kaplan-Meier analysis of progression-free survival (PFS) for (**A**) BCTscore candidate 2 (BCTscore #2) and the BCT biomarkers (**B**) NLR_T3, (**C**) NMR_T2 and (**D**) PLR_T3 comparing between atezolizumab (Ate)-treated patients (dark blue) against docetaxel (Dtx)-treated patients (dark green) in the high-risk (hi) group, and comparing between Ate-treated patients (light blue) against Dtx-treated patients (light green) in the low-risk (lo) group of the training cohort (OAK).

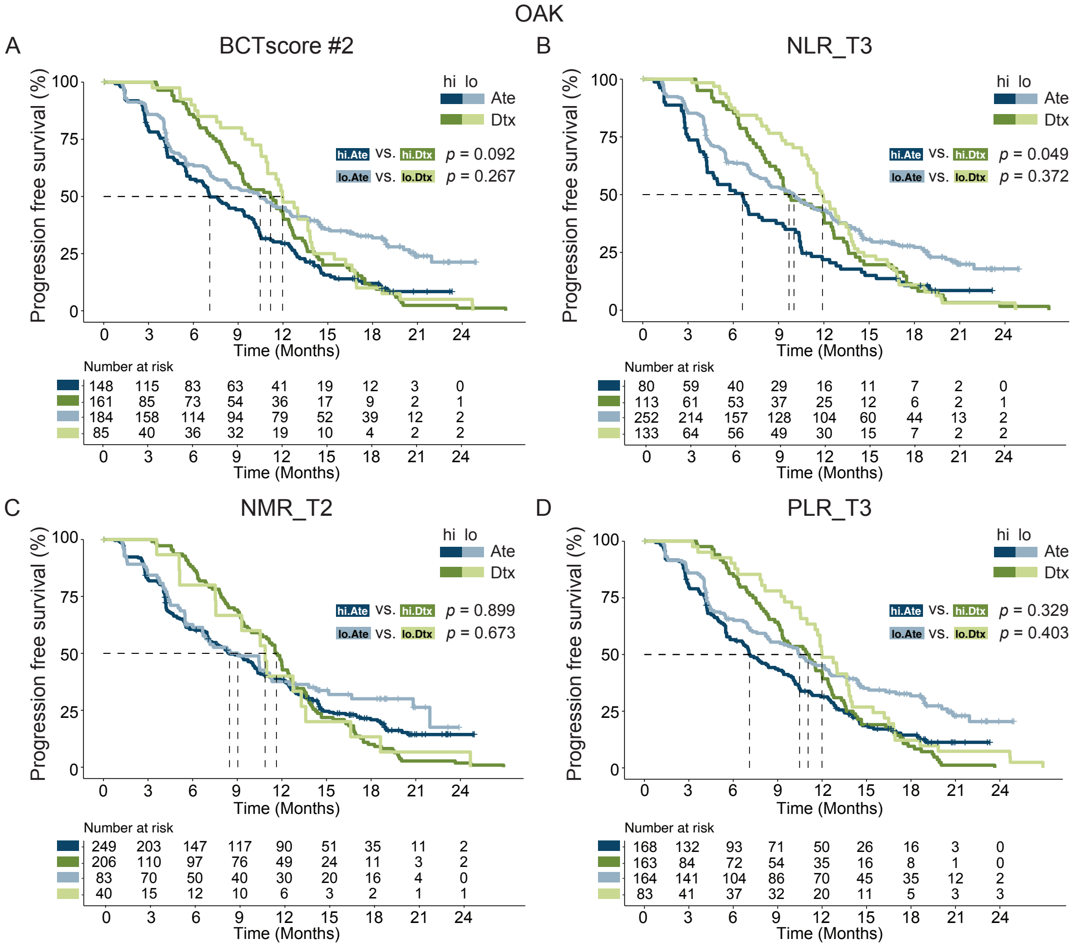


**Supp Figure 9**

**Supplementary Figure S10.** Kaplan-Meier analysis of progression-free survival (PFS) for (**A**) BCTscore candidate 2 (BCTscore #2) and the BCT biomarkers (**B**) NLR_T3, (**C**) NMR_T2 and (**D**) PLR_T3 comparing between atezolizumab (Ate)-treated patients (dark blue) against docetaxel (Dtx)-treated patients (dark green) in the high-risk (hi) group, and comparing between Ate-treated patients (light blue) against Dtx-treated patients (light green) in the low-risk (lo) group of the internal validation cohort (POPLAR).

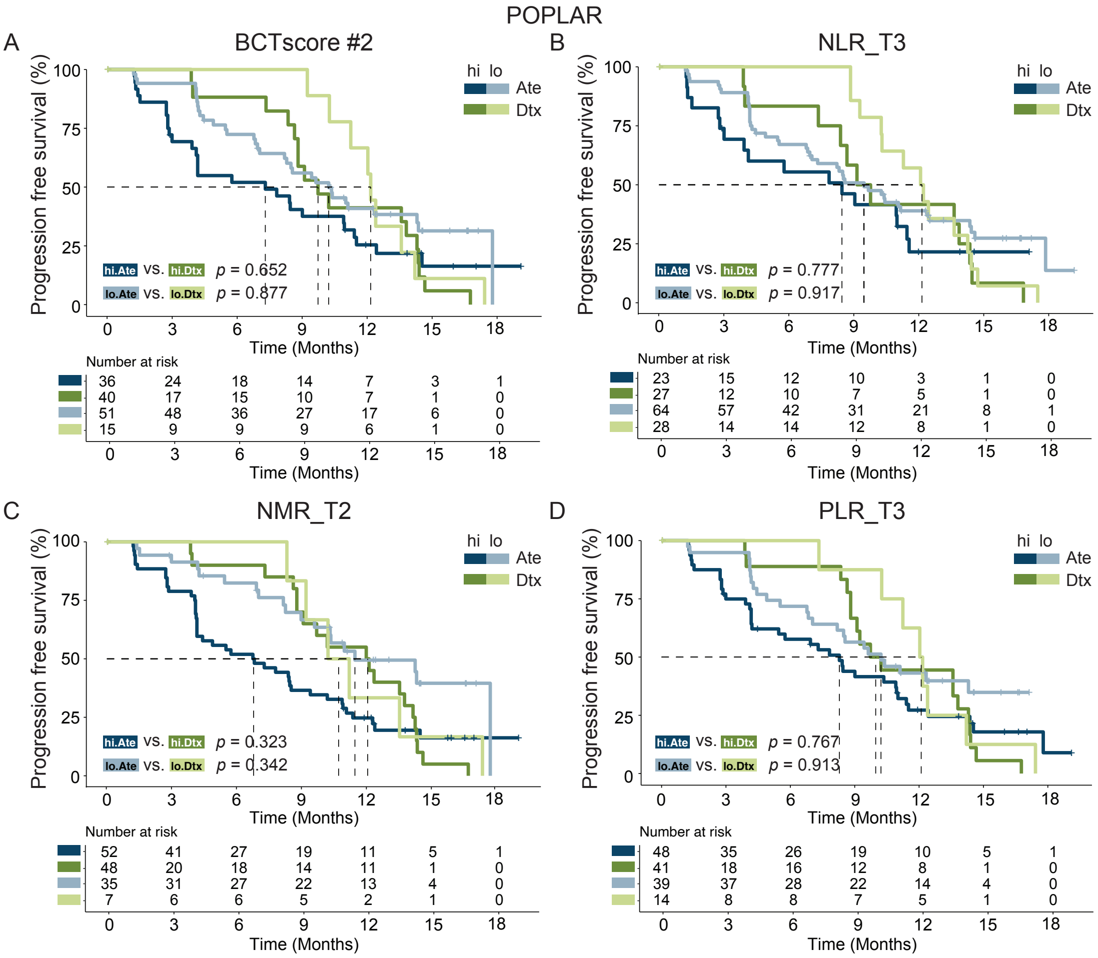


**Supp Figure 10**

**Supp Table 1.**

**Baseline characteristics of atezolizumab- and docetaxel- treated patients in this study.**

| | Atezolizumab-treated Patients | | | | Docetaxel-treated Patients | |
|---|---|---|---|---|---|---|
| | **OAK** | **POPLAR** | **FIR** | **BIRCH** | **OAK** | **POPLAR** |
| **Total** | **609** | **142** | **137** | **667** | **578** | **135** |
| **Age (Years)** | 63 (57-69) | 62 (55-68) | 66 (59-73) | 64 (57-71) | 63 (57-69) | 62 (56-68) |
| **Sex** | | | | | | |
| Male | 376 | 93 | 79 | 393 | 360 | 73 |
| Female | 233 | 49 | 58 | 274 | 218 | 62 |
| **Race** | | | | | | |
| White | 436 | 108 | 122 | 555 | 409 | 109 |
| Asian | 124 | 23 | 0 | 80 | 119 | 13 |
| Other | 27 | 9 | 15 | 21 | 30 | 8 |
| Missing | 22 | 2 | 0 | 11 | 20 | 5 |
| **ECOG PS** | | | | | | |
| 0 | 263 | 51 | 41 | 230 | 259 | 41 |
| 1 | 346 | 91 | 95 | 427 | 319 | 93 |
| ≥2 | 0 | 0 | 0 | 10 | 0 | 0 |
| Missing | 0 | 2 | 1 | 0 | 0 | 1 |
| **Status** | | | | | | |
| Locally advanced | 38 | 8 | 5 | 32 | 32 | 4 |
| Metastatic | 571 | 134 | 132 | 635 | 546 | 131 |
| **Smoking history** | | | | | | |
| Never | 111 | 27 | 19 | 114 | 87 | 26 |

| | | | | | | |
|---|---|---|---|---|---|---|
| Previous | 415 | 90 | 100 | 483 | 389 | 89 |
| Current | 83 | 25 | 18 | 70 | 102 | 20 |
| **PD-L1 expression** | | | | | | |
| Negative | 263 | 51 | 0 | 0 | 263 | 36 |
| Positive | 346 | 91 | 135 | 666 | 346 | 99 |
| Missing | 0 | 0 | 2 | 1 | 0 | 0 |
| **Prior treatment** | | | | | | |
| 0 | 0 | 0 | 32 | 142 | 0 | 1 |
| 1 | 463 | 111 | 52 | 271 | 435 | 108 |
| 2 | 146 | 30 | 34 | 254 | 143 | 25 |
| ≥3 | 0 | 1 | 19 | 0 | 0 | 1 |
| **Histology** | | | | | | |
| Non-squamous | 449 | 93 | 99 | 480 | 422 | 89 |
| Squamous | 160 | 49 | 38 | 187 | 156 | 46 |
| **BMI** | | | | | | |
| 18.5-24.9 (normal weight) | 323 | 73 | 76 | 365 | 324 | 67 |
| 25.0-29.9 (overweight) | 185 | 44 | 44 | 207 | 180 | 41 |
| ≥30.0 (obesity) | 101 | 25 | 17 | 95 | 74 | 27 |

ABBREVIATIONS: ECOG PS, Eastern Cooperative Oncology Group performance status; PD-L1, programmed cell death ligand 1; BMI, bone mass index.

**Supp Table 2.**

**Univariate and multivariate cox analysis of OS and PFS of atezolizumab-treated patients in all four cohorts.**

| Univariate and multivariate cox analysis of overall survival of atezolizumab-treated patients in all four cohorts. | | | | | | | |
|---|---|---|---|---|---|---|---|
| **Factor** | **Description** | **HR.mean** | **HR.lci** | **HR.uci** | **logP** | **Method** | **Outcome** |
| **AGE** | Median Age; continuous variable | 0.99 | 0.99 | 1.00 | 0.071 | Multivariate | OS |
| **SEXM** | Not Female | 1.30 | 1.11 | 1.52 | 0.001 | Multivariate | OS |
| **STATUS** | Metastasis = Yes/No | 0.91 | 0.64 | 1.30 | 0.610 | Multivariate | OS |
| **ECOGGR** | ECOG Performance Status | 1.99 | 1.69 | 2.34 | 0.000 | Multivariate | OS |
| **ASIAN** | Asian | 0.63 | 0.50 | 0.78 | 0.000 | Multivariate | OS |
| **OTHER.race** | Not Asian, Not White | 1.14 | 0.87 | 1.51 | 0.340 | Multivariate | OS |
| **NEVER** | Not Current, Not Previous smoker | 1.28 | 0.96 | 1.69 | 0.090 | Multivariate | OS |
| **PREVIOUS** | Previous smoker | 1.18 | 0.94 | 1.47 | 0.156 | Multivariate | OS |
| **BMI** | Normal, Overweight, Obese | 0.79 | 0.71 | 0.87 | 0.000 | Multivariate | OS |
| **METSITES** | Number of metastatic sites | 1.23 | 1.17 | 1.29 | 0.000 | Multivariate | OS |
| **PD-L1** | PD-L1 >= 1%; BIRCH >= 5% | 0.79 | 0.67 | 0.94 | 0.007 | Multivariate | OS |
| **AGE** | Median Age; continuous variable | 1.00 | 0.99 | 1.00 | 0.192 | Univariate | OS |
| **SEXM** | Not Female | 1.18 | 1.02 | 1.37 | 0.029 | Univariate | OS |
| **STATUS** | Metastasis = Yes/No | 1.23 | 0.88 | 1.73 | 0.230 | Univariate | OS |
| **ECOGGR** | ECOG Performance Status | 2.00 | 1.70 | 2.35 | 0.000 | Univariate | OS |
| **ASIAN** | Asian | 0.77 | 0.63 | 0.95 | 0.016 | Univariate | OS |
| **OTHER.race** | Not Asian, Not White | 1.15 | 0.88 | 1.52 | 0.304 | Univariate | OS |
| **NEVER** | Not Current, Not Previous smoker | 0.93 | 0.77 | 1.13 | 0.469 | Univariate | OS |
| **PREVIOUS** | Previous smoker | 1.08 | 0.92 | 1.26 | 0.336 | Univariate | OS |
| **BMI** | Normal, Overweight, Obese | 0.80 | 0.73 | 0.89 | 0.000 | Univariate | OS |

| | | | | | | | |
|---|---|---|---|---|---|---|---|
| **METSITES** | Number of metastatic sites | 1.26 | 1.20 | 1.32 | 0.000 | Univariate | OS |
| **PD-L1** | PD-L1 >= 1%; BIRCH >= 5% | 0.81 | 0.69 | 0.96 | 0.014 | Univariate | OS |
| Univariate and multivariate cox analysis of progression-free survival of atezolizumab-treated patients in all four cohorts. | | | | | | | |
| Factor | **Description** | **HR.mean** | **HR.lci** | **HR.uci** | **logP** | **Method** | **Outcome** |
| **AGE** | Median Age; continuous variable | 0.99 | 0.98 | 1.00 | 0.000 | Multivariate | PFS |
| **SEXM** | Not Female | 1.14 | 1.00 | 1.30 | 0.043 | Multivariate | PFS |
| **STATUS** | Metastasis = Yes/No | 0.91 | 0.68 | 1.22 | 0.519 | Multivariate | PFS |
| **ECOGGR** | ECOG Performance Status | 1.40 | 1.23 | 1.59 | 0.000 | Multivariate | PFS |
| **ASIAN** | Asian | 0.88 | 0.74 | 1.05 | 0.155 | Multivariate | PFS |
| **OTHER.race** | Not Asian, Not White | 1.08 | 0.86 | 1.37 | 0.503 | Multivariate | PFS |
| **NEVER** | Not Current, Not Previous smoker | 1.71 | 1.35 | 2.16 | 0.000 | Multivariate | PFS |
| **PREVIOUS** | Previous smoker | 1.26 | 1.04 | 1.52 | 0.017 | Multivariate | PFS |
| **BMI** | Normal, Overweight, Obese | 0.91 | 0.84 | 0.99 | 0.033 | Multivariate | PFS |
| **METSITES** | Number of metastatic sites | 1.12 | 1.08 | 1.17 | 0.000 | Multivariate | PFS |
| **PD-L1** | PD-L1 >= 1%; BIRCH >= 5% | 0.83 | 0.72 | 0.95 | 0.009 | Multivariate | PFS |
| **AGE** | Median Age; continuous variable | 0.99 | 0.98 | 1.00 | 0.001 | Univariate | PFS |
| **SEXM** | Not Female | 1.03 | 0.91 | 1.16 | 0.620 | Univariate | PFS |
| **STATUS** | Metastasis = Yes/No | 1.09 | 0.82 | 1.45 | 0.556 | Univariate | PFS |
| **ECOGGR** | ECOG Performance Status | 1.37 | 1.21 | 1.55 | 0.000 | Univariate | PFS |
| **ASIAN** | Asian | 1.05 | 0.89 | 1.23 | 0.592 | Univariate | PFS |
| **OTHER.race** | Not Asian, Not White | 1.09 | 0.87 | 1.38 | 0.452 | Univariate | PFS |
| **NEVER** | Not Current, Not Previous smoker | 1.32 | 1.14 | 1.54 | 0.000 | Univariate | PFS |
| **PREVIOUS** | Previous smoker | 0.93 | 0.82 | 1.06 | 0.297 | Univariate | PFS |
| **BMI** | Normal, Overweight, Obese | 0.91 | 0.84 | 0.99 | 0.025 | Univariate | PFS |

| | | | | | | | |
|---|---|---|---|---|---|---|---|
| **METSITES** | Number of metastatic sites | 1.15 | 1.10 | 1.20 | 0.000 | Univariate | PFS |
| **PD-L1** | PD-L1 >= 1%; BIRCH >= 5% | 0.83 | 0.72 | 0.95 | 0.009 | Univariate | PFS |

ABBREVIATIONS: ECOG, Eastern Cooperative Oncology Group; PD-L1,programmed cell death ligand 1; HR, hazard ratio; lci, lower confidence interval; uci, higher confidence interval; logP, log10 p-value; OS, overall survival; PFS,progression-free survival.

**Supp Table 3.**
**Combinations of BCT biomarkers in different BCTsocre candidates.**

| **BCTscore** | **NLR_T3** | **PLR_T3** | **NMR_T2** |
|---|---|---|---|
| BCTscore#1 | 5 | 170 | 6 |
| BCTscore#2 | 5 | 180 | 6 |
| BCTscore#3 | 5 | 190 | 6 |
| BCTscore#4 | 5 | 200 | 6 |
| BCTscore#5 | 6 | 170 | 6 |
| BCTscore#6 | 6 | 180 | 6 |
| BCTscore#7 | 6 | 190 | 6 |
| BCTscore#8 | 6 | 200 | 6 |
| BCTscore#9 | 5 | 170 | 9 |
| BCTscore#10 | 5 | 180 | 9 |
| BCTscore#11 | 5 | 190 | 9 |
| BCTscore#12 | 5 | 200 | 9 |
| BCTscore#13 | 6 | 170 | 9 |
| BCTscore#14 | 6 | 180 | 9 |
| BCTscore#15 | 6 | 190 | 9 |
| BCTscore#16 | 6 | 200 | 9 |

ABBREVIATIONS: NLR, neutrophile-to-lymphocyte ratio; PLR, platelet-to-lymphocyte ratio; NMR, neutrophile-to-monocyte ratio. T2: 6 weeks on-treatment; T2: 12 weeks on-treatment.

**Supp Table 4.**

**Comprehensive Ranking of different BCTscore candidates by the internal cohorts based on AUC analyses of 4 endpoints.**

A. Ranking of different BCTscore candidates by the internal and external cohorts based on ROC and AUC analyses.

| BCTscore | OAK.rank | BIRCH.rank | POPLAR+FIR.rank | AND.positive | OR.positive |
|---|---|---|---|---|---|
| **BCTscore#1** | 2 | 3 | 0 | 0 | 5 |
| **BCTscore#2** | 5 | 2 | 4 | 11 | 11 |
| **BCTscore#3** | 0 | 1 | 0 | 0 | 1 |
| **BCTscore#4** | 0 | 6 | 0 | 0 | 6 |
| **BCTscore#5** | 3 | 0 | 0 | 0 | 3 |
| **BCTscore#6** | 7 | 0 | 1 | 0 | 8 |
| **BCTscore#7** | 0 | 0 | 0 | 0 | 0 |
| **BCTscore#8** | 0 | 0 | 0 | 0 | 0 |
| **BCTscore#9** | 1 | 0 | 0 | 0 | 1 |
| **BCTscore#10** | 10 | 0 | 3 | 0 | 13 |
| **BCTscore#11** | 9 | 4 | 0 | 0 | 13 |
| **BCTscore#12** | 0 | 5 | 0 | 0 | 5 |
| **BCTscore#13** | 6 | 0 | 0 | 0 | 6 |
| **BCTscore#14** | 8 | 0 | 2 | 0 | 10 |
| **BCTscore#15** | 4 | 7 | 0 | 0 | 11 |
| **BCTscore#16** | 0 | 0 | 0 | 0 | 0 |

Appendix: For rank, the lower the better.

B. Estimation of relative response rate of clinical benefit of atezolizumab-treated patients in the internal cohorts.

| BCTscore | HiRsk.CB | LoRsk.CB | HiRsk.nonCB | LoRsk.nonCB | cohort | HiRsk.CB | LoRsk.CB |
|---|---|---|---|---|---|---|---|
| BCTscore#1 | 59 | 90 | 97 | 85 | OAK.Ate | 38% | 51% |
| BCTscore#2 | 56 | 93 | 91 | 91 | OAK.Ate | 38% | 51% |
| BCTscore#3 | 51 | 98 | 87 | 95 | OAK.Ate | 37% | 51% |
| BCTscore#4 | 50 | 99 | 82 | 100 | OAK.Ate | 38% | 50% |
| BCTscore#5 | 58 | 91 | 94 | 88 | OAK.Ate | 38% | 51% |
| BCTscore#6 | 55 | 94 | 88 | 94 | OAK.Ate | 38% | 50% |
| BCTscore#7 | 49 | 100 | 84 | 98 | OAK.Ate | 37% | 51% |
| BCTscore#8 | 48 | 101 | 79 | 103 | OAK.Ate | 38% | 50% |
| BCTscore#9 | 36 | 113 | 71 | 111 | OAK.Ate | 34% | 50% |
| BCTscore#10 | 34 | 115 | 68 | 114 | OAK.Ate | 33% | 50% |
| BCTscore#11 | 59 | 90 | 98 | 84 | OAK.Ate | 38% | 52% |
| BCTscore#12 | 58 | 91 | 91 | 91 | OAK.Ate | 39% | 50% |
| BCTscore#13 | 32 | 117 | 66 | 116 | OAK.Ate | 33% | 50% |
| BCTscore#14 | 30 | 119 | 63 | 119 | OAK.Ate | 32% | 50% |
| BCTscore#15 | 58 | 91 | 96 | 86 | OAK.Ate | 38% | 51% |
| BCTscore#16 | 57 | 92 | 89 | 93 | OAK.Ate | 39% | 50% |
| BCTscore#1 | 40 | 168 | 70 | 136 | BIRCH.Ate | 36% | 55% |
| BCTscore#2 | 93 | 115 | 114 | 92 | BIRCH.Ate | 45% | 56% |
| BCTscore#3 | 88 | 120 | 110 | 96 | BIRCH.Ate | 44% | 56% |
| BCTscore#4 | 82 | 126 | 105 | 101 | BIRCH.Ate | 44% | 56% |
| BCTscore#5 | 91 | 117 | 113 | 93 | BIRCH.Ate | 45% | 56% |
| BCTscore#6 | 84 | 124 | 109 | 97 | BIRCH.Ate | 44% | 56% |

| | | | | | | | |
|---|---|---|---|---|---|---|---|
| BCTscore#7 | 79 | 129 | 105 | 101 | BIRCH.Ate | 43% | 56% |
| BCTscore#8 | 72 | 136 | 100 | 106 | BIRCH.Ate | 42% | 56% |
| BCTscore#9 | 52 | 156 | 88 | 118 | BIRCH.Ate | 37% | 57% |
| BCTscore#10 | 51 | 157 | 88 | 118 | BIRCH.Ate | 37% | 57% |
| BCTscore#11 | 51 | 157 | 86 | 120 | BIRCH.Ate | 37% | 57% |
| BCTscore#12 | 48 | 160 | 85 | 121 | BIRCH.Ate | 36% | 57% |
| BCTscore#13 | 44 | 164 | 72 | 134 | BIRCH.Ate | 38% | 55% |
| BCTscore#14 | 42 | 166 | 72 | 134 | BIRCH.Ate | 37% | 55% |
| BCTscore#15 | 42 | 166 | 70 | 136 | BIRCH.Ate | 38% | 55% |
| BCTscore#16 | 86 | 122 | 115 | 91 | BIRCH.Ate | 43% | 57% |
| BCTscore#1 | 26 | 39 | 36 | 26 | POPLAR+FIR.Ate | 42% | 60% |
| BCTscore#2 | 22 | 43 | 33 | 29 | POPLAR+FIR.Ate | 40% | 60% |
| BCTscore#3 | 22 | 43 | 31 | 31 | POPLAR+FIR.Ate | 42% | 58% |
| BCTscore#4 | 21 | 44 | 31 | 31 | POPLAR+FIR.Ate | 40% | 59% |
| BCTscore#5 | 23 | 42 | 32 | 30 | POPLAR+FIR.Ate | 42% | 58% |
| BCTscore#6 | 19 | 46 | 29 | 33 | POPLAR+FIR.Ate | 40% | 58% |
| BCTscore#7 | 19 | 46 | 27 | 35 | POPLAR+FIR.Ate | 41% | 57% |
| BCTscore#8 | 18 | 47 | 26 | 36 | POPLAR+FIR.Ate | 41% | 57% |
| BCTscore#9 | 22 | 43 | 30 | 32 | POPLAR+FIR.Ate | 42% | 57% |
| BCTscore#10 | 18 | 47 | 28 | 34 | POPLAR+FIR.Ate | 39% | 58% |
| BCTscore#11 | 18 | 47 | 28 | 34 | POPLAR+FIR.Ate | 39% | 58% |
| BCTscore#12 | 17 | 48 | 28 | 34 | POPLAR+FIR.Ate | 38% | 59% |
| BCTscore#13 | 18 | 47 | 25 | 37 | POPLAR+FIR.Ate | 42% | 56% |
| BCTscore#14 | 14 | 51 | 23 | 39 | POPLAR+FIR.Ate | 38% | 57% |

| | | | | | | | |
|---|---|---|---|---|---|---|---|
| BCTscore#15 | 14 | 51 | 23 | 39 | POPLAR+FIR.Ate | 38% | 57% |
| BCTscore#16 | 27 | 38 | 35 | 27 | POPLAR+FIR.Ate | 44% | 58% |

C. Estimation of relative response rate of objective response of atezolizumab-treated patients in the internal cohorts.

| **BCTscore** | **HiRsk.ORR** | **LoRsk.ORR** | **HiRsk.nonORR** | **LoRsk.nonORR** | **cohort** | **HiRsk.ORR** | **LoRsk.ORR** |
|---|---|---|---|---|---|---|---|
| BCTscore#1 | 27 | 49 | 129 | 126 | OAK.Ate | 17% | 28% |
| BCTscore#2 | 25 | 51 | 122 | 133 | OAK.Ate | 17% | 28% |
| BCTscore#3 | 20 | 56 | 118 | 137 | OAK.Ate | 14% | 29% |
| BCTscore#4 | 19 | 57 | 113 | 142 | OAK.Ate | 14% | 29% |
| BCTscore#5 | 27 | 49 | 125 | 130 | OAK.Ate | 18% | 27% |
| BCTscore#6 | 25 | 51 | 118 | 137 | OAK.Ate | 17% | 27% |
| BCTscore#7 | 20 | 56 | 113 | 142 | OAK.Ate | 15% | 28% |
| BCTscore#8 | 19 | 57 | 108 | 147 | OAK.Ate | 15% | 28% |
| BCTscore#9 | 16 | 60 | 91 | 164 | OAK.Ate | 15% | 27% |
| BCTscore#10 | 15 | 61 | 87 | 168 | OAK.Ate | 15% | 27% |
| BCTscore#11 | 26 | 50 | 131 | 124 | OAK.Ate | 17% | 29% |
| BCTscore#12 | 25 | 51 | 124 | 131 | OAK.Ate | 17% | 28% |
| BCTscore#13 | 16 | 60 | 82 | 173 | OAK.Ate | 16% | 26% |
| BCTscore#14 | 15 | 61 | 78 | 177 | OAK.Ate | 16% | 26% |
| BCTscore#15 | 26 | 50 | 128 | 127 | OAK.Ate | 17% | 28% |
| BCTscore#16 | 25 | 51 | 121 | 134 | OAK.Ate | 17% | 28% |
| BCTscore#1 | 22 | 100 | 88 | 204 | BIRCH.Ate | 20% | 33% |
| BCTscore#2 | 47 | 75 | 160 | 132 | BIRCH.Ate | 23% | 36% |
| BCTscore#3 | 46 | 76 | 152 | 140 | BIRCH.Ate | 23% | 35% |

| | | | | | | | |
|---|---|---|---|---|---|---|---|
| BCTscore#4 | 42 | 80 | 145 | 147 | BIRCH.Ate | 22% | 35% |
| BCTscore#5 | 46 | 76 | 158 | 134 | BIRCH.Ate | 23% | 36% |
| BCTscore#6 | 43 | 79 | 150 | 142 | BIRCH.Ate | 22% | 36% |
| BCTscore#7 | 42 | 80 | 142 | 150 | BIRCH.Ate | 23% | 35% |
| BCTscore#8 | 37 | 85 | 135 | 157 | BIRCH.Ate | 22% | 35% |
| BCTscore#9 | 28 | 94 | 112 | 180 | BIRCH.Ate | 20% | 34% |
| BCTscore#10 | 27 | 95 | 112 | 180 | BIRCH.Ate | 19% | 35% |
| BCTscore#11 | 27 | 95 | 110 | 182 | BIRCH.Ate | 20% | 34% |
| BCTscore#12 | 25 | 97 | 108 | 184 | BIRCH.Ate | 19% | 35% |
| BCTscore#13 | 24 | 98 | 92 | 200 | BIRCH.Ate | 21% | 33% |
| BCTscore#14 | 23 | 99 | 91 | 201 | BIRCH.Ate | 20% | 33% |
| BCTscore#15 | 23 | 99 | 89 | 203 | BIRCH.Ate | 21% | 33% |
| BCTscore#16 | 47 | 75 | 154 | 138 | BIRCH.Ate | 23% | 35% |
| BCTscore#1 | 14 | 20 | 48 | 45 | POPLAR+FIR.Ate | 23% | 31% |
| BCTscore#2 | 10 | 24 | 45 | 48 | POPLAR+FIR.Ate | 18% | 33% |
| BCTscore#3 | 10 | 24 | 43 | 50 | POPLAR+FIR.Ate | 19% | 32% |
| BCTscore#4 | 10 | 24 | 42 | 51 | POPLAR+FIR.Ate | 19% | 32% |
| BCTscore#5 | 12 | 22 | 43 | 50 | POPLAR+FIR.Ate | 22% | 31% |
| BCTscore#6 | 8 | 26 | 40 | 53 | POPLAR+FIR.Ate | 17% | 33% |
| BCTscore#7 | 8 | 26 | 38 | 55 | POPLAR+FIR.Ate | 17% | 32% |
| BCTscore#8 | 8 | 26 | 36 | 57 | POPLAR+FIR.Ate | 18% | 31% |
| BCTscore#9 | 10 | 24 | 42 | 51 | POPLAR+FIR.Ate | 19% | 32% |
| BCTscore#10 | 6 | 28 | 40 | 53 | POPLAR+FIR.Ate | 13% | 35% |
| BCTscore#11 | 6 | 28 | 40 | 53 | POPLAR+FIR.Ate | 13% | 35% |

| | | | | | | | |
|---|---|---|---|---|---|---|---|
| BCTscore#12 | 6 | 28 | 39 | 54 | POPLAR+FIR.Ate | 13% | 34% |
| BCTscore#13 | 8 | 26 | 35 | 58 | POPLAR+FIR.Ate | 19% | 31% |
| BCTscore#14 | 4 | 30 | 33 | 60 | POPLAR+FIR.Ate | 11% | 33% |
| BCTscore#15 | 4 | 30 | 33 | 60 | POPLAR+FIR.Ate | 11% | 33% |
| BCTscore#16 | 14 | 20 | 48 | 45 | POPLAR+FIR.Ate | 23% | 31% |

D. Evaluation of the BCT biomarkers at different cutoff values for application as prognostic and predictive biomarkers by the internal cohorts.

| **BCT biomarker** | **BCT biomarker. cutoff** | **OAK. prognostic_ predictive** | **POPLAR. prognostic_ predictive** | **AND. prognostic_ predictive** | **OAK. predictive** | **POPLAR. predictive** | **AND.predictive** |
|---|---|---|---|---|---|---|---|
| NMR_T2 | 4 | 1 | 0 | 1 | 0 | 0 | 0 |
| NMR_T2 | 5 | 1 | 1 | 2 | 0 | 0 | 0 |
| NMR_T2 | 6 | 1 | 2 | 3 | 0 | 0 | 0 |
| NMR_T2 | 7 | 1 | 1 | 2 | 0 | 0 | 0 |
| NMR_T2 | 8 | 0 | 1 | 1 | 0 | 0 | 0 |
| NMR_T2 | 9 | 0 | 1 | 1 | 0 | 0 | 0 |
| NLR_T3 | 2 | 0 | 0 | 0 | 0 | 0 | 0 |
| NLR_T3 | 3 | 0 | 0 | 0 | 0 | 0 | 0 |
| NLR_T3 | 4 | 2 | 0 | 2 | PFS | 0 | 1 |
| NLR_T3 | 5 | 2 | 0 | 2 | PFS | 0 | 1 |
| NLR_T3 | 6 | 2 | 2 | 4 | PFS | OS | 2 |
| PLR_T3 | 120 | 0 | 0 | 0 | 0 | 0 | 0 |
| PLR_T3 | 130 | 0 | 1 | 1 | 0 | 0 | 0 |

| | | | | | | | |
|---|---|---|---|---|---|---|---|
| PLR_T3 | 140 | 0 | 1 | 1 | 0 | 0 | 0 |
| PLR_T3 | 150 | 0 | 1 | 1 | 0 | 0 | 0 |
| PLR_T3 | 160 | 0 | 0 | 0 | 0 | 0 | 0 |
| PLR_T3 | 170 | 0 | 1 | 1 | 0 | 0 | 0 |
| PLR_T3 | 180 | 1 | 1 | 2 | 0 | 0 | 0 |
| PLR_T3 | 190 | 0 | 1 | 1 | 0 | 0 | 0 |
| PLR_T3 | 200 | 0 | 1 | 1 | 0 | 0 | 0 |
| LMR_T3 | 1 | 0 | 0 | 0 | 0 | 0 | 0 |
| LMR_T3 | 2 | 0 | 0 | 0 | 0 | 0 | 0 |
| LMR_T3 | 3 | 0 | 0 | 0 | 0 | 0 | 0 |
| LMR_T3 | 4 | 1 | 0 | 1 | 0 | 0 | 0 |
| NMR_T3 | 4 | 0 | 0 | 0 | 0 | 0 | 0 |
| NMR_T3 | 5 | 0 | 1 | 1 | 0 | 0 | 0 |
| NMR_T3 | 6 | 0 | 0 | 0 | 0 | 0 | 0 |
| NMR_T3 | 7 | 0 | 0 | 0 | 0 | 0 | 0 |
| NMR_T3 | 8 | 0 | 0 | 0 | 0 | 0 | 0 |
| NMR_T3 | 9 | 0 | 0 | 0 | 0 | 0 | 0 |

E. Evaulation of the BCTscore candidates at different fractions for application as prognostic and predictive biomarkers in the internal cohorts.

| BCTscore | BCTscore. fraction | OAK. prognostic_ predictive_ deltaMedian | POPLAR. prognostic_ predictive_ deltaMedian | AND.prognostic_ predictive_ deltaMedian | OAK. predictive | POPLAR. predictive | Predictive? |
|---|---|---|---|---|---|---|---|
| **BCTscore#1** | 0.1 | 0 | 0 | 0 | 0 | 0 | 0 |
| **BCTscore#1** | 0.2 | 0 | 0 | 0 | 0 | 0 | 0 |
| **BCTscore#1** | 0.25 | 0 | 2 | 1 | 0 | 0 | 0 |
| **BCTscore#1** | 0.3 | 0 | 2 | 1 | 0 | 0 | 0 |
| **BCTscore#1** | 0.4 | 0 | 2 | 1 | 0 | 0 | 0 |
| **BCTscore#1** | 0.5 | 0 | 2 | 1 | 0 | 0 | 0 |
| **BCTscore#1** | 0.6 | 0 | 2 | 1 | 0 | 0 | 0 |
| **BCTscore#1** | 0.7 | 1 | 0 | 1 | 0 | 0 | 0 |
| **BCTscore#1** | 0.75 | 2 | 0 | 1 | PFS | 0 | 1 |
| **BCTscore#1** | 0.8 | 2 | 0 | 1 | PFS | 0 | 1 |
| **BCTscore#1** | 0.9 | 2 | 0 | 1 | PFS | 0 | 1 |
| **BCTscore#2** | 0.1 | 0 | 0 | 0 | 0 | 0 | 0 |
| **BCTscore#2** | 0.2 | 0 | 0 | 0 | 0 | 0 | 0 |
| **BCTscore#2** | 0.25 | 0 | 0 | 0 | 0 | 0 | 0 |
| **BCTscore#2** | 0.3 | 0 | 0 | 0 | 0 | 0 | 0 |
| **BCTscore#2** | 0.4 | 0 | 0 | 0 | 0 | 0 | 0 |
| **BCTscore#2** | 0.5 | 2 | 2 | 2 | 0 | 0 | 0 |
| **BCTscore#2** | 0.6 | 2 | 2 | 2 | 0 | 0 | 0 |

| | | | | | | | |
|---|---|---|---|---|---|---|---|
| **BCTscore#2** | 0.7 | 0 | 0 | 0 | 0 | 0 | 0 |
| **BCTscore#2** | 0.75 | 2 | 0 | 1 | PFS | 0 | 1 |
| **BCTscore#2** | 0.8 | 2 | 0 | 1 | PFS | 0 | 1 |
| **BCTscore#2** | 0.9 | 2 | 0 | 1 | PFS | 0 | 1 |
| **BCTscore#3** | 0.1 | 0 | 0 | 0 | 0 | 0 | 0 |
| **BCTscore#3** | 0.2 | 0 | 0 | 0 | 0 | 0 | 0 |
| **BCTscore#3** | 0.25 | 0 | 2 | 1 | 0 | 0 | 0 |
| **BCTscore#3** | 0.3 | 0 | 2 | 1 | 0 | 0 | 0 |
| **BCTscore#3** | 0.4 | 0 | 2 | 1 | 0 | 0 | 0 |
| **BCTscore#3** | 0.5 | 2 | 2 | 2 | 0 | 0 | 0 |
| **BCTscore#3** | 0.6 | 2 | 2 | 2 | 0 | 0 | 0 |
| **BCTscore#3** | 0.7 | 0 | 0 | 0 | 0 | 0 | 0 |
| **BCTscore#3** | 0.75 | 2 | 0 | 1 | PFS | 0 | 1 |
| **BCTscore#3** | 0.8 | 2 | 0 | 1 | PFS | 0 | 1 |
| **BCTscore#3** | 0.9 | 2 | 0 | 1 | PFS | 0 | 1 |
| **BCTscore#4** | 0.1 | 0 | 0 | 0 | 0 | 0 | 0 |
| **BCTscore#4** | 0.2 | 0 | 0 | 0 | 0 | 0 | 0 |
| **BCTscore#4** | 0.25 | 0 | 2 | 1 | 0 | 0 | 0 |
| **BCTscore#4** | 0.3 | 0 | 2 | 1 | 0 | 0 | 0 |
| **BCTscore#4** | 0.4 | 0 | 2 | 1 | 0 | 0 | 0 |
| **BCTscore#4** | 0.5 | 0 | 2 | 1 | 0 | 0 | 0 |
| **BCTscore#4** | 0.6 | 0 | 2 | 1 | 0 | 0 | 0 |
| **BCTscore#4** | 0.7 | 0 | 0 | 0 | 0 | 0 | 0 |
| **BCTscore#4** | 0.75 | 0 | 1 | 1 | PFS | OS | 2 |

| | | | | | | | |
|---|---|---|---|---|---|---|---|
| **BCTscore#4** | 0.8 | 0 | 1 | 1 | PFS | OS | 2 |
| **BCTscore#4** | 0.9 | 0 | 1 | 1 | PFS | OS | 2 |
| **BCTscore#5** | 0.1 | 0 | 0 | 0 | 0 | 0 | 0 |
| **BCTscore#5** | 0.2 | 0 | 0 | 0 | 0 | 0 | 0 |
| **BCTscore#5** | 0.25 | 0 | 0 | 0 | 0 | 0 | 0 |
| **BCTscore#5** | 0.3 | 0 | 0 | 0 | 0 | 0 | 0 |
| **BCTscore#5** | 0.4 | 0 | 0 | 0 | 0 | 0 | 0 |
| **BCTscore#5** | 0.5 | 0 | 2 | 1 | 0 | 0 | 0 |
| **BCTscore#5** | 0.6 | 0 | 2 | 1 | 0 | 0 | 0 |
| **BCTscore#5** | 0.7 | 0 | 2 | 1 | 0 | 0 | 0 |
| **BCTscore#5** | 0.75 | 0 | 2 | 1 | 0 | OS | 1 |
| **BCTscore#5** | 0.8 | 2 | 2 | 2 | PFS | OS | 2 |
| **BCTscore#5** | 0.9 | 2 | 2 | 2 | PFS | OS | 2 |
| **BCTscore#6** | 0.1 | 0 | 0 | 0 | 0 | 0 | 0 |
| **BCTscore#6** | 0.2 | 0 | 0 | 0 | 0 | 0 | 0 |
| **BCTscore#6** | 0.25 | 0 | 0 | 0 | 0 | 0 | 0 |
| **BCTscore#6** | 0.3 | 0 | 0 | 0 | 0 | 0 | 0 |
| **BCTscore#6** | 0.4 | 0 | 0 | 0 | 0 | 0 | 0 |
| **BCTscore#6** | 0.5 | 0 | 2 | 1 | 0 | 0 | 0 |
| **BCTscore#6** | 0.6 | 0 | 2 | 1 | 0 | 0 | 0 |
| **BCTscore#6** | 0.7 | 0 | 2 | 1 | 0 | 0 | 0 |
| **BCTscore#6** | 0.75 | 0 | 2 | 1 | 0 | OS | 1 |
| **BCTscore#6** | 0.8 | 3 | 2 | 2 | OS | OS | 2 |
| **BCTscore#6** | 0.9 | 3 | 2 | 2 | OS | OS | 2 |

| | | | | | | | | |
|---|---|---|---|---|---|---|---|---|
| **BCTscore#7** | 0.1 | 0 | 0 | 0 | 0 | 0 | 0 |
| **BCTscore#7** | 0.2 | 0 | 0 | 0 | 0 | 0 | 0 |
| **BCTscore#7** | 0.25 | 0 | 0 | 0 | 0 | 0 | 0 |
| **BCTscore#7** | 0.3 | 0 | 2 | 1 | 0 | 0 | 0 |
| **BCTscore#7** | 0.4 | 0 | 2 | 1 | 0 | 0 | 0 |
| **BCTscore#7** | 0.5 | 0 | 2 | 1 | 0 | 0 | 0 |
| **BCTscore#7** | 0.6 | 0 | 3 | 1 | 0 | 0 | 0 |
| **BCTscore#7** | 0.7 | 0 | 3 | 1 | 0 | 0 | 0 |
| **BCTscore#7** | 0.75 | 0 | 0 | 0 | 0 | 0 | 0 |
| **BCTscore#7** | 0.8 | 2 | 2 | 2 | PFS | OS | 2 |
| **BCTscore#7** | 0.9 | 2 | 2 | 2 | PFS | OS | 2 |
| **BCTscore#8** | 0.1 | 0 | 0 | 0 | 0 | 0 | 0 |
| **BCTscore#8** | 0.2 | 0 | 0 | 0 | 0 | 0 | 0 |
| **BCTscore#8** | 0.25 | 0 | 0 | 0 | 0 | 0 | 0 |
| **BCTscore#8** | 0.3 | 0 | 2 | 1 | 0 | 0 | 0 |
| **BCTscore#8** | 0.4 | 0 | 2 | 1 | 0 | 0 | 0 |
| **BCTscore#8** | 0.5 | 0 | 2 | 1 | 0 | 0 | 0 |
| **BCTscore#8** | 0.6 | 1 | 3 | 2 | 0 | 0 | 0 |
| **BCTscore#8** | 0.7 | 1 | 3 | 2 | 0 | 0 | 0 |
| **BCTscore#8** | 0.75 | 0 | 0 | 0 | 0 | 0 | 0 |
| **BCTscore#8** | 0.8 | 0 | 2 | 1 | PFS | OS | 2 |
| **BCTscore#8** | 0.9 | 0 | 2 | 1 | PFS | OS | 2 |
| **BCTscore#9** | 0.1 | 0 | 0 | 0 | 0 | 0 | 0 |
| **BCTscore#9** | 0.2 | 0 | 0 | 0 | 0 | 0 | 0 |

| | | | | | | | |
|---|---|---|---|---|---|---|---|
| **BCTscore#9** | 0.25 | 0 | 0 | 0 | 0 | 0 | 0 |
| **BCTscore#9** | 0.3 | 0 | 0 | 0 | 0 | 0 | 0 |
| **BCTscore#9** | 0.4 | 0 | 0 | 0 | 0 | 0 | 0 |
| **BCTscore#9** | 0.5 | 0 | 0 | 0 | 0 | 0 | 0 |
| **BCTscore#9** | 0.6 | 0 | 3 | 1 | PFS | 0 | 1 |
| **BCTscore#9** | 0.7 | 0 | 0 | 0 | 0 | 0 | 0 |
| **BCTscore#9** | 0.75 | 0 | 0 | 0 | 0 | 0 | 0 |
| **BCTscore#9** | 0.8 | 2 | 0 | 1 | PFS | 0 | 1 |
| **BCTscore#9** | 0.9 | 2 | 0 | 1 | PFS | 0 | 1 |
| **BCTscore#10** | 0.1 | 0 | 0 | 0 | 0 | 0 | 0 |
| **BCTscore#10** | 0.2 | 0 | 0 | 0 | 0 | 0 | 0 |
| **BCTscore#10** | 0.25 | 0 | 0 | 0 | 0 | 0 | 0 |
| **BCTscore#10** | 0.3 | 0 | 0 | 0 | 0 | 0 | 0 |
| **BCTscore#10** | 0.4 | 0 | 0 | 0 | 0 | 0 | 0 |
| **BCTscore#10** | 0.5 | 2 | 0 | 1 | 0 | 0 | 0 |
| **BCTscore#10** | 0.6 | 0 | 3 | 1 | PFS | 0 | 1 |
| **BCTscore#10** | 0.7 | 0 | 0 | 0 | 0 | 0 | 0 |
| **BCTscore#10** | 0.75 | 0 | 0 | 0 | 0 | 0 | 0 |
| **BCTscore#10** | 0.8 | 2 | 0 | 1 | PFS | 0 | 1 |
| **BCTscore#10** | 0.9 | 2 | 0 | 1 | PFS | 0 | 1 |
| **BCTscore#11** | 0.1 | 0 | 0 | 0 | 0 | 0 | 0 |
| **BCTscore#11** | 0.2 | 0 | 0 | 0 | 0 | 0 | 0 |
| **BCTscore#11** | 0.25 | 0 | 0 | 0 | 0 | 0 | 0 |
| **BCTscore#11** | 0.3 | 0 | 2 | 1 | 0 | 0 | 0 |

| | | | | | | | |
|---|---|---|---|---|---|---|---|
| **BCTscore#11** | 0.4 | 0 | 2 | 1 | 0 | 0 | 0 |
| **BCTscore#11** | 0.5 | 0 | 2 | 1 | 0 | 0 | 0 |
| **BCTscore#11** | 0.6 | 2 | 3 | 2 | PFS | 0 | 1 |
| **BCTscore#11** | 0.7 | 1 | 0 | 1 | 0 | 0 | 0 |
| **BCTscore#11** | 0.75 | 2 | 0 | 1 | PFS | 0 | 1 |
| **BCTscore#11** | 0.8 | 2 | 0 | 1 | PFS | 0 | 1 |
| **BCTscore#11** | 0.9 | 2 | 0 | 1 | PFS | 0 | 1 |
| **BCTscore#12** | 0.1 | 0 | 0 | 0 | 0 | 0 | 0 |
| **BCTscore#12** | 0.2 | 0 | 0 | 0 | 0 | 0 | 0 |
| **BCTscore#12** | 0.25 | 0 | 0 | 0 | 0 | 0 | 0 |
| **BCTscore#12** | 0.3 | 0 | 0 | 0 | 0 | 0 | 0 |
| **BCTscore#12** | 0.4 | 0 | 2 | 1 | 0 | 0 | 0 |
| **BCTscore#12** | 0.5 | 0 | 0 | 0 | 0 | 0 | 0 |
| **BCTscore#12** | 0.6 | 2 | 3 | 2 | PFS | 0 | 1 |
| **BCTscore#12** | 0.7 | 1 | 0 | 1 | 0 | 0 | 0 |
| **BCTscore#12** | 0.75 | 0 | 0 | 0 | PFS | 0 | 1 |
| **BCTscore#12** | 0.8 | 2 | 0 | 1 | PFS | 0 | 1 |
| **BCTscore#12** | 0.9 | 2 | 0 | 1 | PFS | 0 | 1 |
| **BCTscore#13** | 0.1 | 0 | 0 | 0 | 0 | 0 | 0 |
| **BCTscore#13** | 0.2 | 0 | 0 | 0 | 0 | 0 | 0 |
| **BCTscore#13** | 0.25 | 0 | 0 | 0 | 0 | 0 | 0 |
| **BCTscore#13** | 0.3 | 0 | 0 | 0 | 0 | 0 | 0 |
| **BCTscore#13** | 0.4 | 0 | 0 | 0 | 0 | 0 | 0 |
| **BCTscore#13** | 0.5 | 0 | 0 | 0 | 0 | 0 | 0 |

| | | | | | | | |
|---|---|---|---|---|---|---|---|
| **BCTscore#13** | 0.6 | 2 | 3 | 2 | PFS | 0 | 1 |
| **BCTscore#13** | 0.7 | 2 | 3 | 2 | PFS | 0 | 1 |
| **BCTscore#13** | 0.75 | 0 | 2 | 1 | 0 | OS | 1 |
| **BCTscore#13** | 0.8 | 0 | 2 | 1 | 0 | OS | 1 |
| **BCTscore#13** | 0.9 | 4 | 0 | 1 | OS | 0 | 1 |
| **BCTscore#14** | 0.1 | 0 | 0 | 0 | 0 | 0 | 0 |
| **BCTscore#14** | 0.2 | 0 | 0 | 0 | 0 | 0 | 0 |
| **BCTscore#14** | 0.25 | 0 | 0 | 0 | 0 | 0 | 0 |
| **BCTscore#14** | 0.3 | 0 | 0 | 0 | 0 | 0 | 0 |
| **BCTscore#14** | 0.4 | 0 | 0 | 0 | 0 | 0 | 0 |
| **BCTscore#14** | 0.5 | 2 | 0 | 1 | 0 | 0 | 0 |
| **BCTscore#14** | 0.6 | 2 | 3 | 2 | 0 | 0 | 0 |
| **BCTscore#14** | 0.7 | 2 | 3 | 2 | PFS | 0 | 1 |
| **BCTscore#14** | 0.75 | 0 | 2 | 1 | 0 | OS | 1 |
| **BCTscore#14** | 0.8 | 0 | 2 | 1 | 0 | OS | 1 |
| **BCTscore#14** | 0.9 | 4 | 0 | 1 | OS | 0 | 1 |
| **BCTscore#15** | 0.1 | 0 | 0 | 0 | 0 | 0 | 0 |
| **BCTscore#15** | 0.2 | 0 | 0 | 0 | 0 | 0 | 0 |
| **BCTscore#15** | 0.25 | 0 | 0 | 0 | 0 | 0 | 0 |
| **BCTscore#15** | 0.3 | 0 | 2 | 1 | 0 | 0 | 0 |
| **BCTscore#15** | 0.4 | 0 | 2 | 1 | 0 | 0 | 0 |
| **BCTscore#15** | 0.5 | 0 | 2 | 1 | 0 | 0 | 0 |
| **BCTscore#15** | 0.6 | 0 | 3 | 1 | 0 | 0 | 0 |
| **BCTscore#15** | 0.7 | 2 | 3 | 2 | PFS | 0 | 1 |

| | | | | | | | |
|---|---|---|---|---|---|---|---|
| **BCTscore#15** | 0.75 | 0 | 2 | 1 | 0 | OS | 1 |
| **BCTscore#15** | 0.8 | 0 | 2 | 1 | 0 | OS | 1 |
| **BCTscore#15** | 0.9 | 4 | 0 | 1 | OS | 0 | 1 |
| **BCTscore#16** | 0.1 | 0 | 0 | 0 | 0 | 0 | 0 |
| **BCTscore#16** | 0.2 | 0 | 0 | 0 | 0 | 0 | 0 |
| **BCTscore#16** | 0.25 | 0 | 0 | 0 | 0 | 0 | 0 |
| **BCTscore#16** | 0.3 | 0 | 0 | 0 | 0 | 0 | 0 |
| **BCTscore#16** | 0.4 | 0 | 2 | 1 | 0 | 0 | 0 |
| **BCTscore#16** | 0.5 | 0 | 0 | 0 | 0 | 0 | 0 |
| **BCTscore#16** | 0.6 | 0 | 0 | 0 | 0 | 0 | 0 |
| **BCTscore#16** | 0.7 | 2 | 3 | 2 | PFS | 0 | 1 |
| **BCTscore#16** | 0.75 | 0 | 0 | 0 | 0 | 0 | 0 |
| **BCTscore#16** | 0.8 | 0 | 2 | 1 | 0 | OS | 1 |
| **BCTscore#16** | 0.9 | 4 | 0 | 1 | OS | 0 | 1 |

F. Estimation of relative response rate of clinical benefit and objective response in the OAK cohort.

| **BCTscore** | **Ate. HiRsk. CB** | **Dtx. HiRsk. CB** | **HiRsk. CB. rate** | **Ate. LoRsk.CB** | **Dtx. LoRsk. CB** | **LoRsk. CB. rate** | **Ate. HiRsk. ORR** | **Dtx. HiRsk. ORR** | **HiRsk. ORR. rate** | **Ate. LoRsk. ORR** | **Dtx. LoRsk. ORR** | **LoRsk. ORR. rate** |
|---|---|---|---|---|---|---|---|---|---|---|---|---|
| BCTscore#1 | 38% | 30% | 1.24 | 51% | 60% | 0.86 | 36% | 34% | 1.04 | 51% | 51% | 1.00 |
| BCTscore#2 | 38% | 30% | 1.25 | 51% | 60% | 0.85 | 33% | 34% | 0.96 | 48% | 51% | 0.95 |
| BCTscore#3 | 37% | 30% | 1.21 | 51% | 59% | 0.86 | 26% | 34% | 0.77 | 46% | 50% | 0.93 |
| BCTscore#4 | 38% | 30% | 1.24 | 50% | 60% | 0.84 | 25% | 34% | 0.73 | 44% | 51% | 0.88 |

| | | | | | | | | | | | | |
|---|---|---|---|---|---|---|---|---|---|---|---|---|
| BCTscore#5 | 38% | 23% | 1.63 | 51% | 50% | 1.01 | 36% | 24% | 1.46 | 49% | 43% | 1.14 |
| BCTscore#6 | 38% | 23% | 1.64 | 50% | 50% | 0.99 | 33% | 24% | 1.35 | 46% | 43% | 1.07 |
| BCTscore#7 | 37% | 23% | 1.57 | 51% | 50% | 1.02 | 26% | 24% | 1.08 | 44% | 43% | 1.04 |
| BCTscore#8 | 38% | 23% | 1.61 | 50% | 50% | 1.00 | 25% | 24% | 1.03 | 42% | 43% | 0.99 |
| BCTscore#9 | 34% | 30% | 1.11 | 50% | 60% | 0.85 | 21% | 34% | 0.61 | 36% | 51% | 0.71 |
| BCTscore#10 | 33% | 30% | 1.13 | 50% | 56% | 0.90 | 20% | 33% | 0.60 | 34% | 48% | 0.71 |
| BCTscore#11 | 38% | 30% | 1.23 | 52% | 60% | 0.87 | 34% | 34% | 1.00 | 51% | 51% | 1.02 |
| BCTscore#12 | 39% | 30% | 1.28 | 50% | 60% | 0.84 | 33% | 34% | 0.96 | 49% | 51% | 0.96 |
| BCTscore#13 | 33% | 23% | 1.39 | 50% | 48% | 1.04 | 21% | 24% | 0.87 | 32% | 41% | 0.78 |
| BCTscore#14 | 32% | 23% | 1.37 | 50% | 47% | 1.06 | 20% | 24% | 0.81 | 31% | 41% | 0.75 |
| BCTscore#15 | 38% | 40% | 0.94 | 51% | 59% | 0.87 | 34% | 43% | 0.80 | 50% | 53% | 0.95 |
| BCTscore#16 | 39% | 23% | 1.66 | 50% | 50% | 0.99 | 33% | 24% | 1.35 | 47% | 43% | 1.10 |

G. Estimation of relative response rate of clinical benefit and objective response in the POPLAR cohort.

| **BCTscore** | **Ate. HiRsk. CB** | **Dtx. HiRsk. CB** | **HiRsk. CB. rate** | **Ate. LoRsk.CB** | **Dtx. LoRsk. CB** | **LoRsk. CB. rate** | **Ate. HiRsk. ORR** | **Dtx. HiRsk. ORR** | **HiRsk. ORR. rate** | **Ate. LoRsk. ORR** | **Dtx. LoRsk. ORR** | **LoRsk. ORR. rate** |
|---|---|---|---|---|---|---|---|---|---|---|---|---|
| BCTscore#1 | 31% | 43% | 0.72 | 59% | 58% | 1.02 | 30% | 32% | 0.95 | 50% | 60% | 0.83 |
| BCTscore#2 | 29% | 43% | 0.67 | 55% | 58% | 0.95 | 25% | 32% | 0.79 | 47% | 60% | 0.78 |
| BCTscore#3 | 29% | 43% | 0.67 | 50% | 58% | 0.87 | 25% | 32% | 0.79 | 44% | 60% | 0.73 |
| BCTscore#4 | 26% | 43% | 0.61 | 50% | 58% | 0.87 | 25% | 32% | 0.79 | 42% | 60% | 0.71 |
| BCTscore#5 | 29% | 32% | 0.89 | 55% | 54% | 1.01 | 25% | 16% | 1.58 | 47% | 57% | 0.82 |
| BCTscore#6 | 26% | 32% | 0.81 | 50% | 54% | 0.93 | 20% | 16% | 1.27 | 44% | 57% | 0.77 |

| | | | | | | | | | | | | |
|---|---|---|---|---|---|---|---|---|---|---|---|---|
| BCTscore#7 | 26% | 32% | 0.81 | 45% | 54% | 0.84 | 20% | 16% | 1.27 | 41% | 57% | 0.72 |
| BCTscore#8 | 24% | 32% | 0.74 | 43% | 54% | 0.80 | 20% | 16% | 1.27 | 38% | 57% | 0.66 |
| BCTscore#9 | 26% | 43% | 0.61 | 48% | 58% | 0.83 | 20% | 32% | 0.63 | 42% | 60% | 0.71 |
| BCTscore#10 | 24% | 39% | 0.61 | 45% | 58% | 0.79 | 15% | 26% | 0.57 | 41% | 60% | 0.68 |
| BCTscore#11 | 24% | 43% | 0.56 | 45% | 58% | 0.79 | 15% | 32% | 0.48 | 41% | 60% | 0.68 |
| BCTscore#12 | 21% | 43% | 0.50 | 45% | 58% | 0.79 | 15% | 32% | 0.48 | 39% | 60% | 0.66 |
| BCTscore#13 | 21% | 29% | 0.75 | 41% | 54% | 0.76 | 15% | 11% | 1.43 | 36% | 57% | 0.64 |
| BCTscore#14 | 19% | 29% | 0.67 | 39% | 54% | 0.72 | 10% | 11% | 0.95 | 35% | 57% | 0.61 |
| BCTscore#15 | 19% | 29% | 0.67 | 39% | 54% | 0.72 | 10% | 11% | 0.95 | 35% | 57% | 0.61 |
| BCTscore#16 | 43% | 32% | 1.33 | 57% | 54% | 1.06 | 50% | 16% | 3.17 | 50% | 57% | 0.88 |

## H. OS and PFS AUC values of BCTscore candidates at each cohorts

| BCTscore | time-point(Month) | OAK | | BIRCH | | FIR+POPLAR | |
|---|---|---|---|---|---|---|---|
| | | OS AUC | PFS AUC | OS AUC | PFS AUC | OS AUC | PFS AUC |
| BCTscore#1 | 12 | 0.674 | 0.580 | 0.689 | 0.651 | 0.720 | 0.588 |
| BCTscore#1 | 18 | 0.677 | 0.645 | 0.656 | 0.676 | 0.591 | 0.473 |
| BCTscore#1 | 24 | 0.645 | 0.606 | 0.656 | 0.676 | 0.591 | 0.473 |
| BCTscore#2 | 12 | 0.696 | 0.588 | 0.672 | 0.653 | 0.727 | 0.621 |
| BCTscore#2 | 18 | 0.684 | 0.655 | 0.642 | 0.681 | 0.621 | 0.598 |
| BCTscore#2 | 24 | 0.646 | 0.605 | 0.642 | 0.681 | 0.621 | 0.598 |
| BCTscore#3 | 12 | 0.678 | 0.589 | 0.680 | 0.648 | 0.736 | 0.612 |
| BCTscore#3 | 18 | 0.661 | 0.648 | 0.648 | 0.670 | 0.604 | 0.537 |
| BCTscore#3 | 24 | 0.634 | 0.603 | 0.648 | 0.670 | 0.604 | 0.537 |

| | | | | | | | |
|---|---|---|---|---|---|---|---|
| BCTscore#4 | 12 | 0.671 | 0.581 | 0.675 | 0.655 | 0.733 | 0.609 |
| BCTscore#4 | 18 | 0.660 | 0.642 | 0.644 | 0.674 | 0.633 | 0.529 |
| BCTscore#4 | 24 | 0.605 | 0.601 | 0.644 | 0.674 | 0.633 | 0.529 |
| BCTscore#5 | 12 | 0.685 | 0.582 | 0.694 | 0.649 | 0.680 | 0.559 |
| BCTscore#5 | 18 | 0.672 | 0.649 | 0.664 | 0.690 | 0.582 | 0.512 |
| BCTscore#5 | 24 | 0.655 | 0.658 | 0.664 | 0.690 | 0.582 | 0.512 |
| BCTscore#6 | 12 | 0.692 | 0.579 | 0.673 | 0.648 | 0.716 | 0.612 |
| BCTscore#6 | 18 | 0.676 | 0.653 | 0.647 | 0.688 | 0.623 | 0.628 |
| BCTscore#6 | 24 | 0.643 | 0.610 | 0.647 | 0.688 | 0.623 | 0.628 |
| BCTscore#7 | 12 | 0.672 | 0.577 | 0.678 | 0.628 | 0.742 | 0.611 |
| BCTscore#7 | 18 | 0.651 | 0.642 | 0.651 | 0.667 | 0.602 | 0.539 |
| BCTscore#7 | 24 | 0.625 | 0.604 | 0.651 | 0.667 | 0.602 | 0.539 |
| BCTscore#8 | 12 | 0.664 | 0.572 | 0.667 | 0.633 | 0.737 | 0.601 |
| BCTscore#8 | 18 | 0.649 | 0.636 | 0.646 | 0.665 | 0.617 | 0.530 |
| BCTscore#8 | 24 | 0.597 | 0.603 | 0.646 | 0.665 | 0.617 | 0.530 |
| BCTscore#9 | 12 | 0.694 | 0.604 | 0.678 | 0.647 | 0.698 | 0.585 |
| BCTscore#9 | 18 | 0.655 | 0.638 | 0.639 | 0.652 | 0.589 | 0.525 |
| BCTscore#9 | 24 | 0.653 | 0.628 | 0.639 | 0.652 | 0.589 | 0.525 |
| BCTscore#10 | 12 | 0.692 | 0.594 | 0.661 | 0.644 | 0.694 | 0.606 |
| BCTscore#10 | 18 | 0.657 | 0.628 | 0.630 | 0.654 | 0.618 | 0.581 |
| BCTscore#10 | 24 | 0.654 | 0.593 | 0.630 | 0.654 | 0.618 | 0.581 |
| BCTscore#11 | 12 | 0.675 | 0.595 | 0.666 | 0.640 | 0.683 | 0.601 |
| BCTscore#11 | 18 | 0.629 | 0.611 | 0.626 | 0.635 | 0.595 | 0.553 |
| BCTscore#11 | 24 | 0.636 | 0.591 | 0.626 | 0.635 | 0.595 | 0.553 |

| | | | | | | | |
|---|---|---|---|---|---|---|---|
| BCTscore#12 | 12 | 0.673 | 0.591 | 0.653 | 0.638 | 0.687 | 0.592 |
| BCTscore#12 | 18 | 0.629 | 0.605 | 0.618 | 0.618 | 0.618 | 0.544 |
| BCTscore#12 | 24 | 0.603 | 0.589 | 0.618 | 0.618 | 0.618 | 0.544 |
| BCTscore#13 | 12 | 0.691 | 0.596 | 0.673 | 0.630 | 0.693 | 0.563 |
| BCTscore#13 | 18 | 0.649 | 0.636 | 0.649 | 0.656 | 0.570 | 0.527 |
| BCTscore#13 | 24 | 0.650 | 0.636 | 0.649 | 0.656 | 0.570 | 0.527 |
| BCTscore#14 | 12 | 0.695 | 0.594 | 0.654 | 0.630 | 0.696 | 0.596 |
| BCTscore#14 | 18 | 0.653 | 0.639 | 0.635 | 0.654 | 0.660 | 0.619 |
| BCTscore#14 | 24 | 0.642 | 0.611 | 0.635 | 0.654 | 0.660 | 0.619 |
| BCTscore#15 | 12 | 0.679 | 0.597 | 0.662 | 0.625 | 0.685 | 0.594 |
| BCTscore#15 | 18 | 0.626 | 0.633 | 0.642 | 0.652 | 0.638 | 0.595 |
| BCTscore#15 | 24 | 0.626 | 0.610 | 0.642 | 0.652 | 0.638 | 0.595 |
| BCTscore#16 | 12 | 0.673 | 0.589 | 0.648 | 0.619 | 0.677 | 0.577 |
| BCTscore#16 | 18 | 0.623 | 0.623 | 0.638 | 0.630 | 0.642 | 0.584 |
| BCTscore#16 | 24 | 0.591 | 0.604 | 0.638 | 0.630 | 0.642 | 0.584 |

ABBREVIATIONS: Ate, atezolizumab; AUC, area under curve; CB, clinical benefit; Dtx, docetaxel; FP, false positivity, HiRsk, high-risk; HR, hazard ratio; lci, lower confidence interval; logP, log10 p-value; LoRsk, low-risk; NLR, neutrophile-to-lymphocyte ratio; NMR, neutrophile-to-monocyte ratio; ORR, objective response rate; OS, overall survival; P, p-value; PFS, progression-free survival; PLR, platelet-to-lymphocyte ratio; ROC, receiver-operating characteristic; RskGrp, risk group; TP, true positivity; uci, higher confidence interval.

**Supp Table 5.**

**Estimation of relative response rate of clinical benefit and objective response of atezolizumab-treated patients in the internal cohorts.**

A. Estimation of relative response rate of clinical benefit of atezolizumab-treated patients in the internal cohorts.

| BCTscore | HiRsk.CB | LoRsk.CB | HiRsk.nonCB | LoRsk.nonCB | cohort | HiRsk.CB | LoRsk.CB |
|---|---|---|---|---|---|---|---|
| BCTscore#1 | 59 | 90 | 97 | 85 | OAK.Ate | 38% | 51% |
| BCTscore#2 | 56 | 93 | 91 | 91 | OAK.Ate | 38% | 51% |
| BCTscore#3 | 51 | 98 | 87 | 95 | OAK.Ate | 37% | 51% |
| BCTscore#4 | 50 | 99 | 82 | 100 | OAK.Ate | 38% | 50% |
| BCTscore#5 | 58 | 91 | 94 | 88 | OAK.Ate | 38% | 51% |
| BCTscore#6 | 55 | 94 | 88 | 94 | OAK.Ate | 38% | 50% |
| BCTscore#7 | 49 | 100 | 84 | 98 | OAK.Ate | 37% | 51% |
| BCTscore#8 | 48 | 101 | 79 | 103 | OAK.Ate | 38% | 50% |
| BCTscore#9 | 36 | 113 | 71 | 111 | OAK.Ate | 34% | 50% |
| BCTscore#10 | 34 | 115 | 68 | 114 | OAK.Ate | 33% | 50% |
| BCTscore#11 | 59 | 90 | 98 | 84 | OAK.Ate | 38% | 52% |
| BCTscore#12 | 58 | 91 | 91 | 91 | OAK.Ate | 39% | 50% |
| BCTscore#13 | 32 | 117 | 66 | 116 | OAK.Ate | 33% | 50% |
| BCTscore#14 | 30 | 119 | 63 | 119 | OAK.Ate | 32% | 50% |
| BCTscore#15 | 58 | 91 | 96 | 86 | OAK.Ate | 38% | 51% |
| BCTscore#16 | 57 | 92 | 89 | 93 | OAK.Ate | 39% | 50% |
| BCTscore#1 | 40 | 168 | 70 | 136 | BIRCH.Ate | 36% | 55% |
| BCTscore#2 | 93 | 115 | 114 | 92 | BIRCH.Ate | 45% | 56% |
| BCTscore#3 | 88 | 120 | 110 | 96 | BIRCH.Ate | 44% | 56% |
| BCTscore#4 | 82 | 126 | 105 | 101 | BIRCH.Ate | 44% | 56% |

| | | | | | | | |
|---|---|---|---|---|---|---|---|
| BCTscore#5 | 91 | 117 | 113 | 93 | BIRCH.Ate | 45% | 56% |
| BCTscore#6 | 84 | 124 | 109 | 97 | BIRCH.Ate | 44% | 56% |
| BCTscore#7 | 79 | 129 | 105 | 101 | BIRCH.Ate | 43% | 56% |
| BCTscore#8 | 72 | 136 | 100 | 106 | BIRCH.Ate | 42% | 56% |
| BCTscore#9 | 52 | 156 | 88 | 118 | BIRCH.Ate | 37% | 57% |
| BCTscore#10 | 51 | 157 | 88 | 118 | BIRCH.Ate | 37% | 57% |
| BCTscore#11 | 51 | 157 | 86 | 120 | BIRCH.Ate | 37% | 57% |
| BCTscore#12 | 48 | 160 | 85 | 121 | BIRCH.Ate | 36% | 57% |
| BCTscore#13 | 44 | 164 | 72 | 134 | BIRCH.Ate | 38% | 55% |
| BCTscore#14 | 42 | 166 | 72 | 134 | BIRCH.Ate | 37% | 55% |
| BCTscore#15 | 42 | 166 | 70 | 136 | BIRCH.Ate | 38% | 55% |
| BCTscore#16 | 86 | 122 | 115 | 91 | BIRCH.Ate | 43% | 57% |
| BCTscore#1 | 26 | 39 | 36 | 26 | POPLAR+FIR.Ate | 42% | 60% |
| BCTscore#2 | 22 | 43 | 33 | 29 | POPLAR+FIR.Ate | 40% | 60% |
| BCTscore#3 | 22 | 43 | 31 | 31 | POPLAR+FIR.Ate | 42% | 58% |
| BCTscore#4 | 21 | 44 | 31 | 31 | POPLAR+FIR.Ate | 40% | 59% |
| BCTscore#5 | 23 | 42 | 32 | 30 | POPLAR+FIR.Ate | 42% | 58% |
| BCTscore#6 | 19 | 46 | 29 | 33 | POPLAR+FIR.Ate | 40% | 58% |
| BCTscore#7 | 19 | 46 | 27 | 35 | POPLAR+FIR.Ate | 41% | 57% |
| BCTscore#8 | 18 | 47 | 26 | 36 | POPLAR+FIR.Ate | 41% | 57% |
| BCTscore#9 | 22 | 43 | 30 | 32 | POPLAR+FIR.Ate | 42% | 57% |
| BCTscore#10 | 18 | 47 | 28 | 34 | POPLAR+FIR.Ate | 39% | 58% |
| BCTscore#11 | 18 | 47 | 28 | 34 | POPLAR+FIR.Ate | 39% | 58% |
| BCTscore#12 | 17 | 48 | 28 | 34 | POPLAR+FIR.Ate | 38% | 59% |

| | | | | | | | |
|---|---|---|---|---|---|---|---|
| BCTscore#13 | 18 | 47 | 25 | 37 | POPLAR+FIR.Ate | 42% | 56% |
| BCTscore#14 | 14 | 51 | 23 | 39 | POPLAR+FIR.Ate | 38% | 57% |
| BCTscore#15 | 14 | 51 | 23 | 39 | POPLAR+FIR.Ate | 38% | 57% |
| BCTscore#16 | 27 | 38 | 35 | 27 | POPLAR+FIR.Ate | 44% | 58% |

B. Estimation of relative response rate of objective response of atezolizumab-treated patients in the internal cohorts.

| **BCTscore** | **HiRsk.ORR** | **LoRsk.ORR** | **HiRsk.nonORR** | **LoRsk.nonORR** | **cohort** | **HiRsk.ORR** | **LoRsk.ORR** |
|---|---|---|---|---|---|---|---|
| BCTscore#1 | 27 | 49 | 129 | 126 | OAK.Ate | 17% | 28% |
| BCTscore#2 | 25 | 51 | 122 | 133 | OAK.Ate | 17% | 28% |
| BCTscore#3 | 20 | 56 | 118 | 137 | OAK.Ate | 14% | 29% |
| BCTscore#4 | 19 | 57 | 113 | 142 | OAK.Ate | 14% | 29% |
| BCTscore#5 | 27 | 49 | 125 | 130 | OAK.Ate | 18% | 27% |
| BCTscore#6 | 25 | 51 | 118 | 137 | OAK.Ate | 17% | 27% |
| BCTscore#7 | 20 | 56 | 113 | 142 | OAK.Ate | 15% | 28% |
| BCTscore#8 | 19 | 57 | 108 | 147 | OAK.Ate | 15% | 28% |
| BCTscore#9 | 16 | 60 | 91 | 164 | OAK.Ate | 15% | 27% |
| BCTscore#10 | 15 | 61 | 87 | 168 | OAK.Ate | 15% | 27% |
| BCTscore#11 | 26 | 50 | 131 | 124 | OAK.Ate | 17% | 29% |
| BCTscore#12 | 25 | 51 | 124 | 131 | OAK.Ate | 17% | 28% |
| BCTscore#13 | 16 | 60 | 82 | 173 | OAK.Ate | 16% | 26% |
| BCTscore#14 | 15 | 61 | 78 | 177 | OAK.Ate | 16% | 26% |
| BCTscore#15 | 26 | 50 | 128 | 127 | OAK.Ate | 17% | 28% |
| BCTscore#16 | 25 | 51 | 121 | 134 | OAK.Ate | 17% | 28% |
| BCTscore#1 | 22 | 100 | 88 | 204 | BIRCH.Ate | 20% | 33% |

| | | | | | | | |
|---|---|---|---|---|---|---|---|
| BCTscore#2 | 47 | 75 | 160 | 132 | BIRCH.Ate | 23% | 36% |
| BCTscore#3 | 46 | 76 | 152 | 140 | BIRCH.Ate | 23% | 35% |
| BCTscore#4 | 42 | 80 | 145 | 147 | BIRCH.Ate | 22% | 35% |
| BCTscore#5 | 46 | 76 | 158 | 134 | BIRCH.Ate | 23% | 36% |
| BCTscore#6 | 43 | 79 | 150 | 142 | BIRCH.Ate | 22% | 36% |
| BCTscore#7 | 42 | 80 | 142 | 150 | BIRCH.Ate | 23% | 35% |
| BCTscore#8 | 37 | 85 | 135 | 157 | BIRCH.Ate | 22% | 35% |
| BCTscore#9 | 28 | 94 | 112 | 180 | BIRCH.Ate | 20% | 34% |
| BCTscore#10 | 27 | 95 | 112 | 180 | BIRCH.Ate | 19% | 35% |
| BCTscore#11 | 27 | 95 | 110 | 182 | BIRCH.Ate | 20% | 34% |
| BCTscore#12 | 25 | 97 | 108 | 184 | BIRCH.Ate | 19% | 35% |
| BCTscore#13 | 24 | 98 | 92 | 200 | BIRCH.Ate | 21% | 33% |
| BCTscore#14 | 23 | 99 | 91 | 201 | BIRCH.Ate | 20% | 33% |
| BCTscore#15 | 23 | 99 | 89 | 203 | BIRCH.Ate | 21% | 33% |
| BCTscore#16 | 47 | 75 | 154 | 138 | BIRCH.Ate | 23% | 35% |
| BCTscore#1 | 14 | 20 | 48 | 45 | POPLAR+FIR.Ate | 23% | 31% |
| BCTscore#2 | 10 | 24 | 45 | 48 | POPLAR+FIR.Ate | 18% | 33% |
| BCTscore#3 | 10 | 24 | 43 | 50 | POPLAR+FIR.Ate | 19% | 32% |
| BCTscore#4 | 10 | 24 | 42 | 51 | POPLAR+FIR.Ate | 19% | 32% |
| BCTscore#5 | 12 | 22 | 43 | 50 | POPLAR+FIR.Ate | 22% | 31% |
| BCTscore#6 | 8 | 26 | 40 | 53 | POPLAR+FIR.Ate | 17% | 33% |
| BCTscore#7 | 8 | 26 | 38 | 55 | POPLAR+FIR.Ate | 17% | 32% |
| BCTscore#8 | 8 | 26 | 36 | 57 | POPLAR+FIR.Ate | 18% | 31% |
| BCTscore#9 | 10 | 24 | 42 | 51 | POPLAR+FIR.Ate | 19% | 32% |

| | | | | | | | |
|---|---|---|---|---|---|---|---|
| BCTscore#10 | 6 | 28 | 40 | 53 | POPLAR+FIR.Ate | 13% | 35% |
| BCTscore#11 | 6 | 28 | 40 | 53 | POPLAR+FIR.Ate | 13% | 35% |
| BCTscore#12 | 6 | 28 | 39 | 54 | POPLAR+FIR.Ate | 13% | 34% |
| BCTscore#13 | 8 | 26 | 35 | 58 | POPLAR+FIR.Ate | 19% | 31% |
| BCTscore#14 | 4 | 30 | 33 | 60 | POPLAR+FIR.Ate | 11% | 33% |
| BCTscore#15 | 4 | 30 | 33 | 60 | POPLAR+FIR.Ate | 11% | 33% |
| BCTscore#16 | 14 | 20 | 48 | 45 | POPLAR+FIR.Ate | 23% | 31% |

ABBREVIATIONS: Ate, atezolizumab; CB, clinical benefit; ORR, objective response rate; HiRsk, high-risk; LoRsk, low-risk.

**Supp Table 6.**

**Comprehensive Evaluation of the BCT biomarkers at different cutoff values for application as prognostic and predictive biomarkers by the internal cohorts.**

A. Evaluation of the BCT biomarkers at different cutoff values for application as prognostic and predictive biomarkers by the internal cohorts.

| BCTbiomarker | BCTbiomarker.cutoff | OAK.prognostic_predictive | POPLAR.prognostic_predictive | AND.prognostic_predictive | OAK.predictive | POPLAR.predictive | AND.predictive |
|---|---|---|---|---|---|---|---|
| NMR_T2 | 4 | 1 | 0 | 1 | 0 | 0 | 0 |
| NMR_T2 | 5 | 1 | 1 | 2 | 0 | 0 | 0 |
| NMR_T2 | 6 | 1 | 2 | 3 | 0 | 0 | 0 |
| NMR_T2 | 7 | 1 | 1 | 2 | 0 | 0 | 0 |
| NMR_T2 | 8 | 0 | 1 | 1 | 0 | 0 | 0 |
| NMR_T2 | 9 | 0 | 1 | 1 | 0 | 0 | 0 |
| NLR_T3 | 2 | 0 | 0 | 0 | 0 | 0 | 0 |
| NLR_T3 | 3 | 0 | 0 | 0 | 0 | 0 | 0 |
| NLR_T3 | 4 | 2 | 0 | 2 | PFS | 0 | 1 |
| NLR_T3 | 5 | 2 | 0 | 2 | PFS | 0 | 1 |
| NLR_T3 | 6 | 2 | 2 | 4 | PFS | OS | 2 |
| PLR_T3 | 120 | 0 | 0 | 0 | 0 | 0 | 0 |
| PLR_T3 | 130 | 0 | 1 | 1 | 0 | 0 | 0 |
| PLR_T3 | 140 | 0 | 1 | 1 | 0 | 0 | 0 |
| PLR_T3 | 150 | 0 | 1 | 1 | 0 | 0 | 0 |
| PLR_T3 | 160 | 0 | 0 | 0 | 0 | 0 | 0 |

| | | | | | | | |
|---|---|---|---|---|---|---|---|
| PLR_T3 | 170 | 0 | 1 | 1 | 0 | 0 | 0 |
| PLR_T3 | 180 | 1 | 1 | 2 | 0 | 0 | 0 |
| PLR_T3 | 190 | 0 | 1 | 1 | 0 | 0 | 0 |
| PLR_T3 | 200 | 0 | 1 | 1 | 0 | 0 | 0 |
| LMR_T3 | 1 | 0 | 0 | 0 | 0 | 0 | 0 |
| LMR_T3 | 2 | 0 | 0 | 0 | 0 | 0 | 0 |
| LMR_T3 | 3 | 0 | 0 | 0 | 0 | 0 | 0 |
| LMR_T3 | 4 | 1 | 0 | 1 | 0 | 0 | 0 |
| NMR_T3 | 4 | 0 | 0 | 0 | 0 | 0 | 0 |
| NMR_T3 | 5 | 0 | 1 | 1 | 0 | 0 | 0 |
| NMR_T3 | 6 | 0 | 0 | 0 | 0 | 0 | 0 |
| NMR_T3 | 7 | 0 | 0 | 0 | 0 | 0 | 0 |
| NMR_T3 | 8 | 0 | 0 | 0 | 0 | 0 | 0 |
| NMR_T3 | 9 | 0 | 0 | 0 | 0 | 0 | 0 |

B. Evaluation of the BCT biomarkers at different cutoff values for application as prognostic and predictive biomarkers in the OAK cohort.

| BCT biomarker | BCT biomarker. cutoff | prognostic_ predictive ,sum | HiRsk. OS | LoRsk.OS | HiRsk .PFS | LoRsk .PFS | HiRsk.OS_ HiRsk.PFS | LoRsk. OS_ LoRsk.PFS | HiRsk . OS_ LoRsk. PFS | LpRsk. OS_ HiRsk . PFS | prognostic. sum | OS. predict | PFS. predict | Predictive? | predictive. sum |
|---|---|---|---|---|---|---|---|---|---|---|---|---|---|---|---|
| NMR_T2 | 4 | 1 | 1 | 0 | 0 | 0 | 0 | 0 | 0 | 0 | 1 | 0 | 0 | 0 | 0 |
| NMR_T2 | 5 | 1 | 1 | 0 | 0 | 0 | 0 | 0 | 0 | 0 | 1 | 0 | 0 | 0 | 0 |
| NMR_T2 | 6 | 1 | 1 | 0 | 0 | 0 | 0 | 0 | 0 | 0 | 1 | 0 | 0 | 0 | 0 |
| NMR_T2 | 7 | 1 | 1 | 0 | 0 | 0 | 0 | 0 | 0 | 0 | 1 | 0 | 0 | 0 | 0 |
| NMR_T2 | 8 | 0 | 0 | 0 | 0 | 0 | 0 | 0 | 0 | 0 | 0 | 0 | 0 | 0 | 0 |
| NMR_T2 | 9 | 0 | 0 | 0 | 0 | 0 | 0 | 0 | 0 | 0 | 0 | 0 | 0 | 0 | 0 |
| NLR_T3 | 2 | 0 | 0 | 0 | 0 | 0 | 0 | 0 | 0 | 0 | 0 | 0 | 0 | 0 | 0 |
| NLR_T3 | 3 | 0 | 0 | 0 | 0 | 0 | 0 | 0 | 0 | 0 | 0 | 0 | 0 | 0 | 0 |
| NLR_T3 | 4 | 2 | 0 | 0 | 1 | 0 | 0 | 0 | 0 | 0 | 1 | 0 | 1 | PFS | 1 |
| NLR_T3 | 5 | 2 | 0 | 0 | 1 | 0 | 0 | 0 | 0 | 0 | 1 | 0 | 1 | PFS | 1 |
| NLR_T3 | 6 | 2 | 0 | 0 | 1 | 0 | 0 | 0 | 0 | 0 | 1 | 0 | 1 | PFS | 1 |
| PLR_T3 | 120 | 0 | 0 | 0 | 0 | 0 | 0 | 0 | 0 | 0 | 0 | 0 | 0 | 0 | 0 |
| PLR_T3 | 130 | 0 | 0 | 0 | 0 | 0 | 0 | 0 | 0 | 0 | 0 | 0 | 0 | 0 | 0 |
| PLR_T3 | 140 | 0 | 0 | 0 | 0 | 0 | 0 | 0 | 0 | 0 | 0 | 0 | 0 | 0 | 0 |
| PLR_T3 | 150 | 0 | 0 | 0 | 0 | 0 | 0 | 0 | 0 | 0 | 0 | 0 | 0 | 0 | 0 |

| | | | | | | | | | | | | | | | |
|---|---|---|---|---|---|---|---|---|---|---|---|---|---|---|---|
| PLR_T3 | 160 | 0 | 0 | 0 | 0 | 0 | 0 | 0 | 0 | 0 | 0 | 0 | 0 | 0 | 0 |
| PLR_T3 | 170 | 0 | 0 | 0 | 0 | 0 | 0 | 0 | 0 | 0 | 0 | 0 | 0 | 0 | 0 |
| PLR_T3 | 180 | 1 | 0 | 1 | 0 | 0 | 0 | 0 | 0 | 0 | 1 | 0 | 0 | 0 | 0 |
| PLR_T3 | 190 | 0 | 0 | 0 | 0 | 0 | 0 | 0 | 0 | 0 | 0 | 0 | 0 | 0 | 0 |
| PLR_T3 | 200 | 0 | 0 | 0 | 0 | 0 | 0 | 0 | 0 | 0 | 0 | 0 | 0 | 0 | 0 |
| LMR_T3 | 1 | 0 | 0 | 0 | 0 | 0 | 0 | 0 | 0 | 0 | 0 | 0 | 0 | 0 | 0 |
| LMR_T3 | 2 | 0 | 0 | 0 | 0 | 0 | 0 | 0 | 0 | 0 | 0 | 0 | 0 | 0 | 0 |
| LMR_T3 | 3 | 0 | 0 | 0 | 0 | 0 | 0 | 0 | 0 | 0 | 0 | 0 | 0 | 0 | 0 |
| LMR_T3 | 4 | 1 | 1 | 0 | 0 | 0 | 0 | 0 | 0 | 0 | 1 | 0 | 0 | 0 | 0 |
| NMR_T3 | 4 | 0 | 0 | 0 | 0 | 0 | 0 | 0 | 0 | 0 | 0 | 0 | 0 | 0 | 0 |
| NMR_T3 | 5 | 0 | 0 | 0 | 0 | 0 | 0 | 0 | 0 | 0 | 0 | 0 | 0 | 0 | 0 |
| NMR_T3 | 6 | 0 | 0 | 0 | 0 | 0 | 0 | 0 | 0 | 0 | 0 | 0 | 0 | 0 | 0 |
| NMR_T3 | 7 | 0 | 0 | 0 | 0 | 0 | 0 | 0 | 0 | 0 | 0 | 0 | 0 | 0 | 0 |
| NMR_T3 | 8 | 0 | 0 | 0 | 0 | 0 | 0 | 0 | 0 | 0 | 0 | 0 | 0 | 0 | 0 |
| NMR_T3 | 9 | 0 | 0 | 0 | 0 | 0 | 0 | 0 | 0 | 0 | 0 | 0 | 0 | 0 | 0 |

C. Kaplan-Meier OS survival analysis of the BCT biomarkers at different cutoff values for application as prognostic biomarkers in the OAK cohort with High-Risk.

| **BCTbiomarker** | **BCT biomarker. cutoff** | **HR.mean** | **HR.lci** | **HR.uci** | **logP** | **Ate.OS.median** | **Ate.OS.lci** | **Ate.OS.uci** | **Dtx.OS.median** | **Dtx.OS.lci** | **Dtx.OS.uci** | **logP<=0.05** |
|---|---|---|---|---|---|---|---|---|---|---|---|---|
| NMR_T2 | 4 | 1.31 | 1.09 | 1.57 | 0.004 | 16.3 | 15.2 | 18.6 | 13.0 | 11.9 | 14.7 | 1 |
| NMR_T2 | 5 | 1.28 | 1.06 | 1.55 | 0.010 | 16.1 | 14.9 | 18.4 | 13.0 | 11.9 | 14.7 | 1 |

| | | | | | | | | | | | | |
|---|---|---|---|---|---|---|---|---|---|---|---|---|
| NMR_T2 | 6 | 1.25 | 1.03 | 1.52 | 0.026 | 15.6 | 14.1 | 17.6 | 12.6 | 11.5 | 14.4 | 1 |
| NMR_T2 | 7 | 1.27 | 1.03 | 1.56 | 0.027 | 15.1 | 14.1 | 17.0 | 12.0 | 11.1 | 13.8 | 1 |
| NMR_T2 | 8 | 1.24 | 0.99 | 1.56 | 0.058 | 14.3 | 13.1 | 16.3 | 12.0 | 11.1 | 13.9 | 0 |
| NMR_T2 | 9 | 1.13 | 0.88 | 1.46 | 0.344 | 14.1 | 11.8 | 17.0 | 12.0 | 11.2 | 14.7 | 0 |
| NLR_T3 | 2 | 1.06 | 0.83 | 1.35 | 0.634 | 17.6 | 15.9 | 20.5 | 16.9 | 14.7 | 19.8 | 0 |
| NLR_T3 | 3 | 0.95 | 0.73 | 1.25 | 0.731 | 15.6 | 13.7 | 18.4 | 16.5 | 13.6 | 18.7 | 0 |
| NLR_T3 | 4 | 0.83 | 0.61 | 1.14 | 0.252 | 14.7 | 12.3 | 17.0 | 16.5 | 13.5 | 20.0 | 0 |
| NLR_T3 | 5 | 0.76 | 0.53 | 1.09 | 0.139 | 13.1 | 10.8 | 17.3 | 17.3 | 13.6 | 20.0 | 0 |
| NLR_T3 | 6 | 0.75 | 0.50 | 1.13 | 0.179 | 13.1 | 10.3 | 19.9 | 17.3 | 13.3 | 23.7 | 0 |
| PLR_T3 | 120 | 1.06 | 0.83 | 1.35 | 0.654 | 18.0 | 15.9 | 20.9 | 17.3 | 15.4 | 19.9 | 0 |
| PLR_T3 | 130 | 1.03 | 0.80 | 1.32 | 0.830 | 17.5 | 15.6 | 20.7 | 17.0 | 14.7 | 19.8 | 0 |
| PLR_T3 | 140 | 1.00 | 0.78 | 1.30 | 0.977 | 17.0 | 15.5 | 20.7 | 17.0 | 14.7 | 19.8 | 0 |
| PLR_T3 | 150 | 1.01 | 0.77 | 1.32 | 0.948 | 17.5 | 15.5 | 20.9 | 17.3 | 14.7 | 19.9 | 0 |
| PLR_T3 | 160 | 1.01 | 0.77 | 1.32 | 0.952 | 17.0 | 15.0 | 20.9 | 17.0 | 14.4 | 19.8 | 0 |
| PLR_T3 | 170 | 0.95 | 0.72 | 1.25 | 0.716 | 16.0 | 14.2 | 20.4 | 17.0 | 14.3 | 19.8 | 0 |
| PLR_T3 | 180 | 0.91 | 0.68 | 1.21 | 0.513 | 15.9 | 14.1 | 19.9 | 17.5 | 14.4 | 19.9 | 0 |
| PLR_T3 | 190 | 0.93 | 0.69 | 1.25 | 0.635 | 15.9 | 14.1 | 20.7 | 17.3 | 14.4 | 20.0 | 0 |
| PLR_T3 | 200 | 0.93 | 0.69 | 1.27 | 0.656 | 15.9 | 13.7 | 20.7 | 17.5 | 14.7 | 20.0 | 0 |
| LMR_T3 | 1 | 1.20 | 0.94 | 1.54 | 0.138 | 20.9 | 18.6 | 24.0 | 18.1 | 16.6 | NA | 0 |
| LMR_T3 | 2 | 1.34 | 0.99 | 1.81 | 0.057 | 22.5 | 20.1 | NA | 18.2 | 16.8 | NA | 0 |
| LMR_T3 | 3 | 1.33 | 0.87 | 2.03 | 0.190 | 23.5 | 20.0 | NA | 19.8 | 16.9 | NA | 0 |
| LMR_T3 | 4 | 2.00 | 1.04 | 3.83 | 0.038 | NA | 23.5 | NA | 16.9 | 13.7 | NA | 1 |
| NMR_T3 | 4 | 1.18 | 0.93 | 1.49 | 0.178 | 20.5 | 18.4 | 23.3 | 17.8 | 16.3 | 20.0 | 0 |
| NMR_T3 | 5 | 1.16 | 0.91 | 1.48 | 0.231 | 20.0 | 17.6 | 22.5 | 17.5 | 15.4 | 20.0 | 0 |

| | | | | | | | | | | | | |
|---|---|---|---|---|---|---|---|---|---|---|---|---|
| NMR_T3 | 6 | 1.20 | 0.94 | 1.54 | 0.150 | 18.7 | 16.3 | 22.5 | 16.9 | 14.3 | 19.5 | 0 |
| NMR_T3 | 7 | 1.14 | 0.87 | 1.50 | 0.351 | 18.5 | 15.9 | 22.0 | 16.8 | 14.0 | 18.7 | 0 |
| NMR_T3 | 8 | 1.11 | 0.82 | 1.51 | 0.493 | 18.4 | 15.0 | 22.0 | 16.8 | 13.6 | 18.7 | 0 |
| NMR_T3 | 9 | 1.06 | 0.76 | 1.47 | 0.735 | 17.0 | 14.1 | 21.3 | 16.8 | 13.6 | 18.7 | 0 |

D. Kaplan-Meier survival analysis of the BCT biomarkers at different cutoff values for application as prognostic biomarkers in the OAK cohort with low-Risk.

| BCTbiomarker | BCTbiomarker.cutoff | HR.mean | HR.lci | HR.uci | logP | Ate.OS.median | Ate.OS.lci | Ate.OS.uci | Dtx.OS.median | Dtx.OS.lci | Dtx.OS.uci | logP<=0.05 |
|---|---|---|---|---|---|---|---|---|---|---|---|---|
| NMR_T2 | 4 | 0.73 | 0.26 | 2.02 | 0.543 | 20.2 | 16.7 | NA | NA | 17.9 | NA | 0 |
| NMR_T2 | 5 | 1.17 | 0.61 | 2.22 | 0.644 | 22.2 | 16.7 | NA | 24.7 | 12.7 | NA | 0 |
| NMR_T2 | 6 | 1.20 | 0.75 | 1.91 | 0.445 | 22.5 | 19.9 | NA | 24.7 | 13.6 | NA | 0 |
| NMR_T2 | 7 | 1.08 | 0.74 | 1.56 | 0.696 | 20.4 | 18.0 | NA | 19.4 | 16.6 | NA | 0 |
| NMR_T2 | 8 | 1.10 | 0.80 | 1.51 | 0.547 | 20.0 | 17.6 | 22.2 | 18.6 | 13.6 | NA | 0 |
| NMR_T2 | 9 | 1.29 | 0.99 | 1.68 | 0.059 | 18.6 | 16.4 | 22.0 | 14.6 | 12.7 | 19.4 | 0 |
| NLR_T3 | 2 | 1.34 | 0.59 | 3.05 | 0.498 | NA | 23.5 | NA | 24.7 | 24.7 | NA | 0 |
| NLR_T3 | 3 | 1.25 | 0.77 | 2.04 | 0.376 | NA | 22.5 | NA | 24.7 | 19.8 | NA | 0 |
| NLR_T3 | 4 | 1.36 | 0.95 | 1.94 | 0.097 | 24.0 | 21.3 | NA | 19.8 | 16.9 | NA | 0 |
| NLR_T3 | 5 | 1.33 | 0.98 | 1.82 | 0.072 | 23.5 | 20.4 | NA | 19.4 | 16.6 | NA | 0 |
| NLR_T3 | 6 | 1.28 | 0.96 | 1.71 | 0.098 | 22.2 | 20.1 | NA | 18.6 | 16.3 | NA | 0 |
| PLR_T3 | 120 | 1.94 | 0.97 | 3.89 | 0.067 | NA | 23.5 | NA | 24.7 | 16.9 | NA | 0 |
| PLR_T3 | 130 | 1.66 | 0.90 | 3.07 | 0.110 | NA | 23.5 | NA | 24.7 | 16.9 | NA | 0 |
| PLR_T3 | 140 | 1.64 | 0.96 | 2.79 | 0.077 | NA | 23.5 | NA | 24.7 | 16.9 | NA | 0 |
| PLR_T3 | 150 | 1.51 | 0.94 | 2.44 | 0.096 | NA | 21.3 | NA | 24.7 | 16.5 | NA | 0 |

| | | | | | | | | | | | | |
|---|---|---|---|---|---|---|---|---|---|---|---|---|
| PLR_T3 | 160 | 1.42 | 0.90 | 2.23 | 0.138 | NA | 21.3 | NA | 24.7 | 16.5 | NA | 0 |
| PLR_T3 | 170 | 1.50 | 0.98 | 2.31 | 0.068 | NA | 21.3 | NA | 24.7 | 16.5 | NA | 0 |
| PLR_T3 | 180 | 1.58 | 1.06 | 2.35 | 0.027 | NA | 21.3 | NA | 19.8 | 16.2 | NA | 1 |
| PLR_T3 | 190 | 1.44 | 0.99 | 2.10 | 0.058 | NA | 20.5 | NA | 18.7 | 16.5 | NA | 0 |
| PLR_T3 | 200 | 1.43 | 1.00 | 2.04 | 0.056 | 22.5 | 20.2 | NA | 18.7 | 15.4 | NA | 0 |
| LMR_T3 | 1 | 0.92 | 0.44 | 1.93 | 0.823 | 14.7 | 10.1 | NA | 14.4 | 9.2 | NA | 0 |
| LMR_T3 | 2 | 0.93 | 0.65 | 1.34 | 0.702 | 15.6 | 12.9 | 20.7 | 16.8 | 12.7 | NA | 0 |
| LMR_T3 | 3 | 1.11 | 0.84 | 1.46 | 0.467 | 18.7 | 15.9 | 22.0 | 17.0 | 14.7 | 24.7 | 0 |
| LMR_T3 | 4 | 1.08 | 0.84 | 1.39 | 0.531 | 18.7 | 16.3 | 21.9 | 17.8 | 16.2 | 23.7 | 0 |
| NMR_T3 | 4 | 1.42 | 0.47 | 4.27 | 0.528 | NA | 16.7 | NA | 24.7 | 15.9 | NA | 0 |
| NMR_T3 | 5 | 1.59 | 0.70 | 3.62 | 0.270 | NA | 20.2 | NA | 24.7 | 15.9 | NA | 0 |
| NMR_T3 | 6 | 0.99 | 0.52 | 1.88 | 0.975 | 22.2 | 20.2 | NA | 24.7 | 24.7 | NA | 0 |
| NMR_T3 | 7 | 1.14 | 0.74 | 1.76 | 0.565 | 23.3 | 20.2 | NA | 24.7 | 19.8 | NA | 0 |
| NMR_T3 | 8 | 1.12 | 0.77 | 1.61 | 0.559 | 22.2 | 19.9 | NA | 24.7 | 18.6 | NA | 0 |
| NMR_T3 | 9 | 1.11 | 0.78 | 1.56 | 0.570 | 22.0 | 19.9 | NA | 24.7 | 16.8 | NA | 0 |

E. Kaplan-Meier survival analysis of the BCT biomarkers at different cutoff values for application as prognostic biomarkers in the OAK cohort with High-Risk.

| **BCT biomarker** | **BCT biomarker. cutoff** | **HR.mean** | **HR.lci** | **HR.uci** | **logP** | **Ate. PFS. median** | **Ate. PFS. lci** | **Ate. PFS. uci** | **Dtx. PFS. median** | **Dtx. PFS. lci** | **Dtx. PFS. uci** | **logP <=0.05** |
|---|---|---|---|---|---|---|---|---|---|---|---|---|
| NMR_T2 | 4 | 1.01 | 0.85 | 1.20 | 0.887 | 5.6 | 4.7 | 7.1 | 8.6 | 7.9 | 9.7 | 0 |
| NMR_T2 | 5 | 1.00 | 0.84 | 1.19 | 0.987 | 5.6 | 4.5 | 7.1 | 8.8 | 8.0 | 9.7 | 0 |
| NMR_T2 | 6 | 0.97 | 0.81 | 1.17 | 0.751 | 5.6 | 4.4 | 7.1 | 8.4 | 7.9 | 9.5 | 0 |

| | | | | | | | | | | | | |
|---|---|---|---|---|---|---|---|---|---|---|---|---|
| NMR_T2 | 7 | 0.94 | 0.77 | 1.14 | 0.508 | 5.6 | 4.3 | 7.0 | 8.4 | 7.8 | 9.6 | 0 |
| NMR_T2 | 8 | 0.85 | 0.69 | 1.05 | 0.137 | 4.9 | 4.1 | 6.8 | 8.8 | 7.6 | 9.8 | 0 |
| NMR_T2 | 9 | 0.85 | 0.67 | 1.08 | 0.179 | 4.7 | 4.0 | 6.8 | 8.1 | 6.9 | 9.3 | 0 |
| NLR_T3 | 2 | 0.93 | 0.74 | 1.17 | 0.536 | 7.7 | 7.0 | 9.4 | 11.5 | 10.5 | 12.5 | 0 |
| NLR_T3 | 3 | 0.85 | 0.66 | 1.09 | 0.190 | 7.2 | 5.8 | 9.0 | 11.1 | 9.4 | 12.0 | 0 |
| NLR_T3 | 4 | 0.71 | 0.53 | 0.95 | 0.019 | 6.1 | 4.9 | 7.9 | 10.7 | 9.1 | 12.0 | 1 |
| NLR_T3 | 5 | 0.69 | 0.49 | 0.97 | 0.030 | 6.6 | 4.3 | 8.8 | 9.8 | 9.0 | 12.6 | 1 |
| NLR_T3 | 6 | 0.67 | 0.46 | 0.99 | 0.044 | 6.1 | 4.3 | 10.1 | 9.8 | 8.4 | 12.6 | 1 |
| PLR_T3 | 120 | 0.94 | 0.74 | 1.18 | 0.575 | 7.9 | 7.0 | 9.7 | 11.4 | 10.0 | 12.5 | 0 |
| PLR_T3 | 130 | 0.93 | 0.73 | 1.17 | 0.534 | 7.6 | 7.0 | 9.7 | 11.5 | 10.0 | 12.5 | 0 |
| PLR_T3 | 140 | 0.94 | 0.74 | 1.20 | 0.641 | 7.4 | 6.7 | 9.7 | 11.3 | 9.7 | 12.5 | 0 |
| PLR_T3 | 150 | 0.95 | 0.74 | 1.22 | 0.707 | 7.4 | 6.7 | 9.4 | 11.2 | 9.4 | 12.4 | 0 |
| PLR_T3 | 160 | 0.97 | 0.75 | 1.25 | 0.818 | 7.7 | 6.7 | 10.1 | 11.2 | 9.4 | 12.5 | 0 |
| PLR_T3 | 170 | 0.88 | 0.68 | 1.13 | 0.314 | 7.1 | 6.1 | 9.3 | 11.1 | 9.3 | 12.4 | 0 |
| PLR_T3 | 180 | 0.86 | 0.66 | 1.12 | 0.259 | 7.2 | 5.8 | 9.4 | 11.1 | 9.3 | 12.4 | 0 |
| PLR_T3 | 190 | 0.84 | 0.63 | 1.10 | 0.203 | 7.1 | 5.6 | 9.4 | 11.0 | 9.2 | 12.4 | 0 |
| PLR_T3 | 200 | 0.83 | 0.63 | 1.11 | 0.206 | 7.2 | 5.8 | 9.7 | 11.0 | 9.2 | 12.5 | 0 |
| LMR_T3 | 1 | 1.08 | 0.87 | 1.36 | 0.484 | 9.0 | 7.3 | 10.4 | 11.9 | 11.0 | 12.7 | 0 |
| LMR_T3 | 2 | 1.19 | 0.91 | 1.57 | 0.212 | 10.1 | 8.2 | 12.3 | 12.0 | 11.2 | 13.5 | 0 |
| LMR_T3 | 3 | 1.24 | 0.85 | 1.83 | 0.272 | 9.7 | 7.6 | 13.5 | 11.8 | 10.5 | 13.7 | 0 |
| LMR_T3 | 4 | 1.45 | 0.83 | 2.51 | 0.199 | 10.1 | 7.6 | 15.1 | 11.1 | 9.7 | 14.0 | 0 |
| NMR_T3 | 4 | 1.05 | 0.85 | 1.31 | 0.644 | 8.5 | 7.2 | 10.3 | 11.5 | 10.7 | 12.5 | 0 |
| NMR_T3 | 5 | 1.05 | 0.84 | 1.31 | 0.687 | 8.5 | 7.1 | 10.2 | 11.5 | 10.5 | 12.6 | 0 |
| NMR_T3 | 6 | 0.98 | 0.78 | 1.23 | 0.851 | 8.2 | 7.0 | 9.7 | 11.6 | 10.7 | 12.7 | 0 |

| | | | | | | | | | | | | |
|---|---|---|---|---|---|---|---|---|---|---|---|---|
| NMR_T3 | 7 | 0.97 | 0.75 | 1.25 | 0.801 | 7.8 | 7.0 | 9.7 | 11.6 | 10.5 | 12.6 | 0 |
| NMR_T3 | 8 | 0.98 | 0.74 | 1.29 | 0.874 | 7.6 | 6.1 | 9.7 | 11.5 | 9.7 | 12.6 | 0 |
| NMR_T3 | 9 | 0.93 | 0.69 | 1.25 | 0.619 | 7.3 | 5.8 | 9.4 | 11.5 | 9.7 | 12.6 | 0 |

F. Kaplan-Meier survival analysis of the BCT biomarkers at different cutoff values for application as prognostic biomarkers in the OAK cohort with low-Risk.

| **BCTbiomarker** | **BCTbiomarker.cutoff** | **HR.mean** | **HR.lci** | **HR.uci** | **logP** | **Ate.PFS.median** | **Ate.PFS.lci** | **Ate.PFS.uci** | **Dtx.PFS.median** | **Dtx.PFS.lci** | **Dtx.PFS.uci** | **logP<=0.05** |
|---|---|---|---|---|---|---|---|---|---|---|---|---|
| NMR_T2 | 4 | 0.95 | 0.38 | 2.39 | 0.920 | 6.7 | 5.5 | 12.5 | 8.7 | 5.1 | NA | 0 |
| NMR_T2 | 5 | 1.05 | 0.58 | 1.89 | 0.884 | 6.7 | 5.5 | 10.5 | 7.3 | 5.1 | 16.8 | 0 |
| NMR_T2 | 6 | 1.08 | 0.70 | 1.66 | 0.731 | 6.7 | 5.2 | 10.5 | 10.3 | 7.5 | 12.7 | 0 |
| NMR_T2 | 7 | 1.07 | 0.76 | 1.52 | 0.700 | 6.7 | 5.0 | 9.4 | 9.5 | 7.9 | 13.0 | 0 |
| NMR_T2 | 8 | 1.13 | 0.84 | 1.52 | 0.437 | 7.2 | 5.5 | 10.4 | 8.6 | 7.9 | 10.9 | 0 |
| NMR_T2 | 9 | 1.05 | 0.82 | 1.34 | 0.696 | 7.0 | 5.5 | 9.2 | 9.5 | 8.3 | 11.0 | 0 |
| NLR_T3 | 2 | 1.45 | 0.68 | 3.12 | 0.358 | 13.5 | 10.0 | 18.9 | 12.3 | 11.0 | NA | 0 |
| NLR_T3 | 3 | 1.15 | 0.73 | 1.81 | 0.549 | 11.3 | 9.2 | 13.8 | 13.7 | 11.7 | 16.6 | 0 |
| NLR_T3 | 4 | 1.19 | 0.86 | 1.66 | 0.302 | 10.8 | 9.0 | 12.6 | 12.7 | 11.5 | 13.9 | 0 |
| NLR_T3 | 5 | 1.14 | 0.86 | 1.52 | 0.365 | 10.1 | 8.3 | 12.2 | 11.9 | 11.3 | 13.5 | 0 |
| NLR_T3 | 6 | 1.11 | 0.85 | 1.45 | 0.437 | 9.4 | 8.2 | 10.9 | 11.9 | 11.1 | 13.5 | 0 |
| PLR_T3 | 120 | 1.41 | 0.76 | 2.62 | 0.291 | 12.3 | 9.4 | 19.0 | 12.3 | 11.5 | 18.6 | 0 |
| PLR_T3 | 130 | 1.21 | 0.69 | 2.13 | 0.516 | 11.3 | 9.2 | 14.8 | 11.9 | 10.5 | 16.9 | 0 |
| PLR_T3 | 140 | 1.11 | 0.68 | 1.82 | 0.682 | 10.8 | 8.5 | 14.5 | 12.0 | 10.5 | 16.9 | 0 |
| PLR_T3 | 150 | 1.08 | 0.69 | 1.68 | 0.745 | 10.5 | 8.3 | 13.8 | 12.7 | 11.5 | 15.4 | 0 |
| PLR_T3 | 160 | 1.05 | 0.69 | 1.60 | 0.819 | 10.3 | 8.2 | 12.3 | 12.0 | 11.5 | 14.0 | 0 |

| | | | | | | | | | | | | |
|---|---|---|---|---|---|---|---|---|---|---|---|---|
| PLR_T3 | 170 | 1.15 | 0.78 | 1.70 | 0.493 | 10.5 | 8.3 | 13.5 | 12.4 | 11.7 | 14.0 | 0 |
| PLR_T3 | 180 | 1.17 | 0.81 | 1.68 | 0.407 | 10.5 | 8.3 | 12.5 | 12.0 | 11.5 | 13.8 | 0 |
| PLR_T3 | 190 | 1.16 | 0.82 | 1.64 | 0.410 | 10.4 | 8.3 | 12.5 | 12.4 | 11.5 | 13.8 | 0 |
| PLR_T3 | 200 | 1.16 | 0.83 | 1.62 | 0.392 | 10.3 | 7.8 | 12.5 | 12.0 | 11.5 | 13.7 | 0 |
| LMR_T3 | 1 | 0.66 | 0.32 | 1.36 | 0.245 | 4.7 | 3.0 | 10.5 | 9.2 | 7.1 | NA | 0 |
| LMR_T3 | 2 | 0.76 | 0.54 | 1.08 | 0.121 | 6.7 | 5.0 | 9.3 | 9.5 | 8.3 | 12.4 | 0 |
| LMR_T3 | 3 | 0.93 | 0.72 | 1.20 | 0.580 | 7.9 | 6.7 | 10.3 | 11.5 | 9.5 | 12.5 | 0 |
| LMR_T3 | 4 | 0.97 | 0.77 | 1.23 | 0.815 | 8.3 | 7.0 | 10.2 | 11.9 | 10.7 | 12.7 | 0 |
| NMR_T3 | 4 | 0.82 | 0.30 | 2.23 | 0.696 | 8.0 | 3.2 | NA | 12.7 | 5.1 | NA | 0 |
| NMR_T3 | 5 | 0.89 | 0.43 | 1.84 | 0.759 | 8.2 | 5.5 | 16.7 | 12.1 | 9.2 | NA | 0 |
| NMR_T3 | 6 | 1.25 | 0.70 | 2.23 | 0.465 | 10.8 | 8.3 | 14.5 | 10.9 | 9.2 | 16.6 | 0 |
| NMR_T3 | 7 | 1.12 | 0.74 | 1.68 | 0.595 | 10.4 | 7.4 | 12.3 | 11.6 | 9.5 | 13.9 | 0 |
| NMR_T3 | 8 | 1.03 | 0.73 | 1.46 | 0.852 | 10.3 | 7.8 | 11.3 | 12.1 | 10.5 | 13.9 | 0 |
| NMR_T3 | 9 | 1.04 | 0.75 | 1.44 | 0.807 | 10.1 | 7.8 | 11.3 | 11.9 | 10.5 | 13.8 | 0 |

G. Kaplan-Meier OS survival analysis of the BCT biomarkers at different cutoff values for application as predictive biomarkers in the OAK cohort.

| **BCTbiomarker** | **BCT biomarker. cutoff** | **cutoff** | **HR. mean** | **HR. lci** | **HR. uci** | **P** | **cutoff** | **HR. mean** | **HR. lci** | **HR. uci** | **P** | **Predictive?** |
|---|---|---|---|---|---|---|---|---|---|---|---|---|
| NMR_T2 | 4 | >cut-off | 1.31 | 1.09 | 1.57 | 0.004 | <cut-off | 0.73 | 0.26 | 2.02 | 0.548 | 0 |
| NMR_T2 | 5 | >cut-off | 1.28 | 1.06 | 1.55 | 0.009 | <cut-off | 1.17 | 0.61 | 2.22 | 0.642 | 0 |
| NMR_T2 | 6 | >cut-off | 1.25 | 1.03 | 1.52 | 0.025 | <cut-off | 1.20 | 0.75 | 1.91 | 0.442 | 0 |
| NMR_T2 | 7 | >cut-off | 1.27 | 1.03 | 1.56 | 0.027 | <cut-off | 1.08 | 0.74 | 1.56 | 0.695 | 0 |

| | | | | | | | | | | | | |
|---|---|---|---|---|---|---|---|---|---|---|---|---|
| NMR_T2 | 8 | >cut-off | 1.24 | 0.99 | 1.56 | 0.059 | <cut-off | 1.10 | 0.80 | 1.51 | 0.545 | 0 |
| NMR_T2 | 9 | >cut-off | 1.13 | 0.88 | 1.46 | 0.345 | <cut-off | 1.29 | 0.99 | 1.68 | 0.056 | 0 |
| NLR_T3 | 2 | >cut-off | 1.06 | 0.83 | 1.35 | 0.634 | <cut-off | 1.34 | 0.59 | 3.05 | 0.489 | 0 |
| NLR_T3 | 3 | >cut-off | 0.95 | 0.73 | 1.25 | 0.731 | <cut-off | 1.25 | 0.77 | 2.04 | 0.368 | 0 |
| NLR_T3 | 4 | >cut-off | 0.83 | 0.61 | 1.14 | 0.251 | <cut-off | 1.36 | 0.95 | 1.94 | 0.092 | 0 |
| NLR_T3 | 5 | >cut-off | 0.76 | 0.53 | 1.09 | 0.137 | <cut-off | 1.33 | 0.98 | 1.82 | 0.068 | 0 |
| NLR_T3 | 6 | >cut-off | 0.75 | 0.50 | 1.13 | 0.175 | <cut-off | 1.28 | 0.96 | 1.71 | 0.094 | 0 |
| PLR_T3 | 120 | >cut-off | 1.06 | 0.83 | 1.35 | 0.654 | <cut-off | 1.94 | 0.97 | 3.89 | 0.061 | 0 |
| PLR_T3 | 130 | >cut-off | 1.03 | 0.80 | 1.32 | 0.830 | <cut-off | 1.66 | 0.90 | 3.07 | 0.102 | 0 |
| PLR_T3 | 140 | >cut-off | 1.00 | 0.78 | 1.30 | 0.977 | <cut-off | 1.64 | 0.96 | 2.79 | 0.071 | 0 |
| PLR_T3 | 150 | >cut-off | 1.01 | 0.77 | 1.32 | 0.948 | <cut-off | 1.51 | 0.94 | 2.44 | 0.090 | 0 |
| PLR_T3 | 160 | >cut-off | 1.01 | 0.77 | 1.32 | 0.952 | <cut-off | 1.42 | 0.90 | 2.23 | 0.132 | 0 |
| PLR_T3 | 170 | >cut-off | 0.95 | 0.72 | 1.25 | 0.716 | <cut-off | 1.50 | 0.98 | 2.31 | 0.063 | 0 |
| PLR_T3 | 180 | >cut-off | 0.91 | 0.68 | 1.21 | 0.514 | <cut-off | 1.58 | 1.06 | 2.35 | 0.024 | 0 |
| PLR_T3 | 190 | >cut-off | 0.93 | 0.69 | 1.25 | 0.635 | <cut-off | 1.44 | 0.99 | 2.10 | 0.054 | 0 |
| PLR_T3 | 200 | >cut-off | 0.93 | 0.69 | 1.27 | 0.656 | <cut-off | 1.43 | 1.00 | 2.04 | 0.052 | 0 |
| LMR_T3 | 1 | >cut-off | 1.20 | 0.94 | 1.54 | 0.136 | <cut-off | 0.92 | 0.44 | 1.93 | 0.824 | 0 |
| LMR_T3 | 2 | >cut-off | 1.34 | 0.99 | 1.81 | 0.055 | <cut-off | 0.93 | 0.65 | 1.34 | 0.702 | 0 |
| LMR_T3 | 3 | >cut-off | 1.33 | 0.87 | 2.03 | 0.186 | <cut-off | 1.11 | 0.84 | 1.46 | 0.466 | 0 |
| LMR_T3 | 4 | >cut-off | 2.00 | 1.04 | 3.83 | 0.038 | <cut-off | 1.08 | 0.84 | 1.39 | 0.530 | 0 |
| NMR_T3 | 4 | >cut-off | 1.18 | 0.93 | 1.49 | 0.177 | <cut-off | 1.42 | 0.47 | 4.27 | 0.529 | 0 |
| NMR_T3 | 5 | >cut-off | 1.16 | 0.91 | 1.48 | 0.229 | <cut-off | 1.59 | 0.70 | 3.62 | 0.270 | 0 |
| NMR_T3 | 6 | >cut-off | 1.20 | 0.94 | 1.54 | 0.148 | <cut-off | 0.99 | 0.52 | 1.88 | 0.975 | 0 |
| NMR_T3 | 7 | >cut-off | 1.14 | 0.87 | 1.50 | 0.350 | <cut-off | 1.14 | 0.74 | 1.76 | 0.562 | 0 |

| | | | | | | | | | | | | |
|---|---|---|---|---|---|---|---|---|---|---|---|---|
| NMR_T3 | 8 | >cut-off | 1.11 | 0.82 | 1.51 | 0.493 | <cut-off | 1.12 | 0.77 | 1.61 | 0.557 | 0 |
| NMR_T3 | 9 | >cut-off | 1.06 | 0.76 | 1.47 | 0.735 | <cut-off | 1.11 | 0.78 | 1.56 | 0.568 | 0 |

H. Kaplan-Meier PFS survival analysis of the BCT biomarkers at different cutoff values for application as predictive biomarkers in the OAK cohort.

| BCTbiomarker | BCT biomarker. cutoff | cutoff | HR.mean | HR.lci | HR.uci | P | cutoff | HR.mean | HR.lci | HR.uci | P | Predictive? |
|---|---|---|---|---|---|---|---|---|---|---|---|---|
| NMR_T2 | 4 | >cut-off | 1.01 | 0.85 | 1.20 | 0.887 | <cut-off | 0.95 | 0.38 | 2.39 | 0.920 | 0 |
| NMR_T2 | 5 | >cut-off | 1.00 | 0.84 | 1.19 | 0.987 | <cut-off | 1.05 | 0.58 | 1.89 | 0.884 | 0 |
| NMR_T2 | 6 | >cut-off | 0.97 | 0.81 | 1.17 | 0.751 | <cut-off | 1.08 | 0.70 | 1.66 | 0.729 | 0 |
| NMR_T2 | 7 | >cut-off | 0.94 | 0.77 | 1.14 | 0.508 | <cut-off | 1.07 | 0.76 | 1.52 | 0.698 | 0 |
| NMR_T2 | 8 | >cut-off | 0.85 | 0.69 | 1.05 | 0.137 | <cut-off | 1.13 | 0.84 | 1.52 | 0.432 | 0 |
| NMR_T2 | 9 | >cut-off | 0.85 | 0.67 | 1.08 | 0.179 | <cut-off | 1.05 | 0.82 | 1.34 | 0.695 | 0 |
| NLR_T3 | 2 | >cut-off | 0.93 | 0.74 | 1.17 | 0.537 | <cut-off | 1.45 | 0.68 | 3.12 | 0.338 | 0 |
| NLR_T3 | 3 | >cut-off | 0.85 | 0.66 | 1.09 | 0.192 | <cut-off | 1.15 | 0.73 | 1.81 | 0.543 | 0 |
| NLR_T3 | 4 | >cut-off | 0.71 | 0.53 | 0.95 | 0.020 | <cut-off | 1.19 | 0.86 | 1.66 | 0.295 | 1 |
| NLR_T3 | 5 | >cut-off | 0.69 | 0.49 | 0.97 | 0.031 | <cut-off | 1.14 | 0.86 | 1.52 | 0.360 | 1 |
| NLR_T3 | 6 | >cut-off | 0.67 | 0.46 | 0.99 | 0.044 | <cut-off | 1.11 | 0.85 | 1.45 | 0.433 | 1 |
| PLR_T3 | 120 | >cut-off | 0.94 | 0.74 | 1.18 | 0.576 | <cut-off | 1.41 | 0.76 | 2.62 | 0.275 | 0 |
| PLR_T3 | 130 | >cut-off | 0.93 | 0.73 | 1.17 | 0.535 | <cut-off | 1.21 | 0.69 | 2.13 | 0.507 | 0 |
| PLR_T3 | 140 | >cut-off | 0.94 | 0.74 | 1.20 | 0.642 | <cut-off | 1.11 | 0.68 | 1.82 | 0.679 | 0 |
| PLR_T3 | 150 | >cut-off | 0.95 | 0.74 | 1.22 | 0.708 | <cut-off | 1.08 | 0.69 | 1.68 | 0.744 | 0 |
| PLR_T3 | 160 | >cut-off | 0.97 | 0.75 | 1.25 | 0.818 | <cut-off | 1.05 | 0.69 | 1.60 | 0.819 | 0 |

| | | | | | | | | | | | | |
|---|---|---|---|---|---|---|---|---|---|---|---|---|
| PLR_T3 | 170 | >cut-off | 0.88 | 0.68 | 1.13 | 0.316 | <cut-off | 1.15 | 0.78 | 1.70 | 0.488 | 0 |
| PLR_T3 | 180 | >cut-off | 0.86 | 0.66 | 1.12 | 0.261 | <cut-off | 1.17 | 0.81 | 1.68 | 0.402 | 0 |
| PLR_T3 | 190 | >cut-off | 0.84 | 0.63 | 1.10 | 0.206 | <cut-off | 1.16 | 0.82 | 1.64 | 0.404 | 0 |
| PLR_T3 | 200 | >cut-off | 0.83 | 0.63 | 1.11 | 0.209 | <cut-off | 1.16 | 0.83 | 1.62 | 0.386 | 0 |
| LMR_T3 | 1 | >cut-off | 1.08 | 0.87 | 1.36 | 0.482 | <cut-off | 0.66 | 0.32 | 1.36 | 0.254 | 0 |
| LMR_T3 | 2 | >cut-off | 1.19 | 0.91 | 1.57 | 0.207 | <cut-off | 0.76 | 0.54 | 1.08 | 0.126 | 0 |
| LMR_T3 | 3 | >cut-off | 1.24 | 0.85 | 1.83 | 0.266 | <cut-off | 0.93 | 0.72 | 1.20 | 0.582 | 0 |
| LMR_T3 | 4 | >cut-off | 1.45 | 0.83 | 2.51 | 0.191 | <cut-off | 0.97 | 0.77 | 1.23 | 0.815 | 0 |
| NMR_T3 | 4 | >cut-off | 1.05 | 0.85 | 1.31 | 0.643 | <cut-off | 0.82 | 0.30 | 2.23 | 0.698 | 0 |
| NMR_T3 | 5 | >cut-off | 1.05 | 0.84 | 1.31 | 0.687 | <cut-off | 0.89 | 0.43 | 1.84 | 0.761 | 0 |
| NMR_T3 | 6 | >cut-off | 0.98 | 0.78 | 1.23 | 0.852 | <cut-off | 1.25 | 0.70 | 2.23 | 0.456 | 0 |
| NMR_T3 | 7 | >cut-off | 0.97 | 0.75 | 1.25 | 0.801 | <cut-off | 1.12 | 0.74 | 1.68 | 0.591 | 0 |
| NMR_T3 | 8 | >cut-off | 0.98 | 0.74 | 1.29 | 0.874 | <cut-off | 1.03 | 0.73 | 1.46 | 0.851 | 0 |
| NMR_T3 | 9 | >cut-off | 0.93 | 0.69 | 1.25 | 0.620 | <cut-off | 1.04 | 0.75 | 1.44 | 0.806 | 0 |

I. Evaluation of the BCT biomarkers at different cutoff values for application as prognostic and predictive biomarkers in the POPLAR cohort.

| **BCT biomarker** | **BCT biomarker. cutoff** | **prognostic_ predictive ,sum** | **HiRsk. OS** | **LoRsk.OS** | **HiRsk .PFS** | **LoRsk .PFS** | **HiRsk.OS_ HiRsk.PFS** | **LoRsk. OS_ LoRsk.PFS** | **HiRsk. OS_ LoRsk. PFS** | **LpRsk. OS_ HiRsk. PFS** | **prognostic. sum** | **OS. predict** | **PFS. predict** | **Predictive?** | **predictive. sum** |
|---|---|---|---|---|---|---|---|---|---|---|---|---|---|---|---|

| | | | | | | | | | | | | | | | |
|---|---|---|---|---|---|---|---|---|---|---|---|---|---|---|---|
| NMR_T2 | 4 | 0 | 0 | 0 | 0 | 0 | 0 | 0 | 0 | 0 | 0 | 0 | 0 | 0 | 0 |
| NMR_T2 | 5 | 1 | 0 | 1 | 0 | 0 | 0 | 0 | 0 | 0 | 1 | 0 | 0 | 0 | 0 |
| NMR_T2 | 6 | 2 | 0 | 1 | 0 | 1 | 0 | 1 | 0 | 0 | 2 | 0 | 0 | 0 | 0 |
| NMR_T2 | 7 | 1 | 0 | 1 | 0 | 0 | 0 | 0 | 0 | 0 | 1 | 0 | 0 | 0 | 0 |
| NMR_T2 | 8 | 1 | 0 | 1 | 0 | 0 | 0 | 0 | 0 | 0 | 1 | 0 | 0 | 0 | 0 |
| NMR_T2 | 9 | 1 | 0 | 1 | 0 | 0 | 0 | 0 | 0 | 0 | 1 | 0 | 0 | 0 | 0 |
| NLR_T3 | 2 | 0 | 0 | 0 | 0 | 0 | 0 | 0 | 0 | 0 | 0 | 0 | 0 | 0 | 0 |
| NLR_T3 | 3 | 0 | 0 | 0 | 0 | 0 | 0 | 0 | 0 | 0 | 0 | 0 | 0 | 0 | 0 |
| NLR_T3 | 4 | 0 | 0 | 0 | 0 | 0 | 0 | 0 | 0 | 0 | 0 | 0 | 0 | 0 | 0 |
| NLR_T3 | 5 | 0 | 0 | 0 | 0 | 0 | 0 | 0 | 0 | 0 | 0 | 0 | 0 | 0 | 0 |
| NLR_T3 | 6 | 2 | 1 | 0 | 0 | 0 | 0 | 0 | 0 | 0 | 1 | 1 | 0 | OS | 1 |
| PLR_T3 | 120 | 0 | 0 | 0 | 0 | 0 | 0 | 0 | 0 | 0 | 0 | 0 | 0 | 0 | 0 |
| PLR_T3 | 130 | 1 | 0 | 1 | 0 | 0 | 0 | 0 | 0 | 0 | 1 | 0 | 0 | 0 | 0 |
| PLR_T3 | 140 | 1 | 0 | 1 | 0 | 0 | 0 | 0 | 0 | 0 | 1 | 0 | 0 | 0 | 0 |
| PLR_T3 | 150 | 1 | 0 | 1 | 0 | 0 | 0 | 0 | 0 | 0 | 1 | 0 | 0 | 0 | 0 |
| PLR_T3 | 160 | 0 | 0 | 0 | 0 | 0 | 0 | 0 | 0 | 0 | 0 | 0 | 0 | 0 | 0 |
| PLR_T3 | 170 | 1 | 0 | 1 | 0 | 0 | 0 | 0 | 0 | 0 | 1 | 0 | 0 | 0 | 0 |
| PLR_T3 | 180 | 1 | 0 | 1 | 0 | 0 | 0 | 0 | 0 | 0 | 1 | 0 | 0 | 0 | 0 |
| PLR_T3 | 190 | 1 | 0 | 1 | 0 | 0 | 0 | 0 | 0 | 0 | 1 | 0 | 0 | 0 | 0 |
| PLR_T3 | 200 | 1 | 0 | 1 | 0 | 0 | 0 | 0 | 0 | 0 | 1 | 0 | 0 | 0 | 0 |
| LMR_T3 | 1 | 0 | 0 | 0 | 0 | 0 | 0 | 0 | 0 | 0 | 0 | 0 | 0 | 0 | 0 |
| LMR_T3 | 2 | 0 | 0 | 0 | 0 | 0 | 0 | 0 | 0 | 0 | 0 | 0 | 0 | 0 | 0 |
| LMR_T3 | 3 | 0 | 0 | 0 | 0 | 0 | 0 | 0 | 0 | 0 | 0 | 0 | 0 | 0 | 0 |
| LMR_T3 | 4 | 0 | 0 | 0 | 0 | 0 | 0 | 0 | 0 | 0 | 0 | 0 | 0 | 0 | 0 |

| | | | | | | | | | | | | | | |
|---|---|---|---|---|---|---|---|---|---|---|---|---|---|---|
| NMR_T3 | 4 | 0 | 0 | 0 | 0 | 0 | 0 | 0 | 0 | 0 | 0 | 0 | 0 | 0 | 0 |
| NMR_T3 | 5 | 1 | 0 | 1 | 0 | 0 | 0 | 0 | 0 | 0 | 1 | 0 | 0 | 0 | 0 |
| NMR_T3 | 6 | 0 | 0 | 0 | 0 | 0 | 0 | 0 | 0 | 0 | 0 | 0 | 0 | 0 | 0 |
| NMR_T3 | 7 | 0 | 0 | 0 | 0 | 0 | 0 | 0 | 0 | 0 | 0 | 0 | 0 | 0 | 0 |
| NMR_T3 | 8 | 0 | 0 | 0 | 0 | 0 | 0 | 0 | 0 | 0 | 0 | 0 | 0 | 0 | 0 |
| NMR_T3 | 9 | 0 | 0 | 0 | 0 | 0 | 0 | 0 | 0 | 0 | 0 | 0 | 0 | 0 | 0 |

J. Kaplan-Meier survival analysis of the BCT biomarkers at different cutoff values for application as prognostic biomarkers in the POPLAR cohort with high-risk.

| BCTbiomarker | BCTbiomarker.cutoff | HR.mean | HR.lci | HR.uci | logP | Ate.OS.median | Ate.OS.lci | Ate.OS.uci | Dtx.OS.median | Dtx.OS.lci | Dtx.OS.uci | logP<=0.05 |
|---|---|---|---|---|---|---|---|---|---|---|---|---|
| NMR_T2 | 4 | 1.27 | 0.84 | 1.93 | 0.251 | NA | 14.5 | NA | 14.2 | 12.4 | 17.4 | 0 |
| NMR_T2 | 5 | 1.16 | 0.76 | 1.76 | 0.493 | 15.5 | 12.4 | NA | 14.3 | 12.4 | 17.4 | 0 |
| NMR_T2 | 6 | 0.83 | 0.53 | 1.31 | 0.429 | 12.4 | 9.8 | NA | 14.4 | 12.9 | NA | 0 |
| NMR_T2 | 7 | 0.80 | 0.49 | 1.31 | 0.376 | 12.4 | 9.8 | NA | 14.4 | 12.9 | NA | 0 |
| NMR_T2 | 8 | 0.64 | 0.37 | 1.10 | 0.106 | 11.0 | 8.5 | NA | 14.4 | 12.9 | NA | 0 |
| NMR_T2 | 9 | 0.61 | 0.33 | 1.12 | 0.113 | 10.0 | 7.3 | NA | 16.0 | 12.9 | NA | 0 |
| NLR_T3 | 2 | 1.06 | 0.62 | 1.82 | 0.834 | NA | 15.1 | NA | 16.8 | 14.3 | NA | 0 |
| NLR_T3 | 3 | 0.78 | 0.42 | 1.42 | 0.406 | 15.5 | 11.4 | NA | 16.8 | 14.3 | NA | 0 |
| NLR_T3 | 4 | 0.70 | 0.35 | 1.42 | 0.324 | 14.0 | 10.9 | NA | 16.8 | 14.3 | NA | 0 |
| NLR_T3 | 5 | 0.51 | 0.23 | 1.12 | 0.093 | 11.2 | 9.6 | NA | 16.8 | 14.3 | NA | 0 |
| NLR_T3 | 6 | 0.40 | 0.17 | 0.97 | 0.047 | 9.6 | 7.8 | NA | NA | 13.8 | NA | 1 |
| PLR_T3 | 120 | 1.03 | 0.60 | 1.77 | 0.917 | NA | 14.5 | NA | 14.7 | 14.2 | NA | 0 |

| | | | | | | | | | | | | |
|---|---|---|---|---|---|---|---|---|---|---|---|---|
| PLR_T3 | 130 | 0.93 | 0.53 | 1.65 | 0.813 | NA | 14.5 | NA | 16.8 | 14.3 | NA | 0 |
| PLR_T3 | 140 | 0.89 | 0.50 | 1.60 | 0.702 | NA | 14.5 | NA | 16.8 | 14.4 | NA | 0 |
| PLR_T3 | 150 | 0.83 | 0.47 | 1.50 | 0.541 | 15.5 | 12.6 | NA | 16.8 | 14.4 | NA | 0 |
| PLR_T3 | 160 | 0.89 | 0.50 | 1.61 | 0.704 | 15.5 | 13.0 | NA | 16.8 | 14.3 | NA | 0 |
| PLR_T3 | 170 | 0.82 | 0.45 | 1.52 | 0.535 | 15.5 | 12.6 | NA | 16.8 | 14.4 | NA | 0 |
| PLR_T3 | 180 | 0.73 | 0.40 | 1.35 | 0.314 | 15.1 | 11.4 | NA | 16.8 | 14.4 | NA | 0 |
| PLR_T3 | 190 | 0.70 | 0.37 | 1.33 | 0.269 | 15.1 | 11.4 | NA | 16.8 | 14.4 | NA | 0 |
| PLR_T3 | 200 | 0.66 | 0.33 | 1.33 | 0.239 | 15.1 | 11.4 | NA | NA | 14.4 | NA | 0 |
| LMR_T3 | 1 | 1.31 | 0.74 | 2.30 | 0.361 | NA | 16.4 | NA | 16.8 | 14.2 | NA | 0 |
| LMR_T3 | 2 | 1.22 | 0.58 | 2.56 | 0.599 | NA | 16.4 | NA | NA | 14.2 | NA | 0 |
| LMR_T3 | 3 | 1.08 | 0.41 | 2.83 | 0.880 | NA | 16.4 | NA | NA | 13.6 | NA | 0 |
| LMR_T3 | 4 | 3.83 | 0.74 | 19.81 | 0.083 | NA | 16.4 | NA | 14.4 | 10.2 | NA | 0 |
| NMR_T3 | 4 | 1.18 | 0.69 | 2.02 | 0.547 | NA | 15.5 | NA | 16.8 | 14.3 | NA | 0 |
| NMR_T3 | 5 | 1.05 | 0.60 | 1.82 | 0.870 | NA | 15.1 | NA | 16.8 | 14.3 | NA | 0 |
| NMR_T3 | 6 | 0.99 | 0.54 | 1.80 | 0.962 | NA | 12.6 | NA | 16.8 | 14.2 | NA | 0 |
| NMR_T3 | 7 | 0.88 | 0.45 | 1.72 | 0.704 | NA | 11.4 | NA | 16.8 | 13.8 | NA | 0 |
| NMR_T3 | 8 | 0.67 | 0.33 | 1.35 | 0.261 | 12.6 | 10.0 | NA | 16.8 | 13.8 | NA | 0 |
| NMR_T3 | 9 | 0.70 | 0.32 | 1.56 | 0.391 | 13.0 | 9.8 | NA | 14.4 | 13.6 | NA | 0 |

K. Kaplan-Meier survival analysis of the BCT biomarkers at different cutoff values for application as prognostic biomarkers in the POPLAR cohort with low-risk.

| BCTbio marker | BCT | HR.m ean | H R.lci | HR .uci | log P | Ate . | Ate .OS. lci | Ate .OS. uci | Dtx. | Dtx .OS. lci | Dtx .OS. uci | logP< =0.05 |
|---|---|---|---|---|---|---|---|---|---|---|---|---|

| | biomarker.cutoff | | | | | OS.median | | | OS.median | | | |
|---|---|---|---|---|---|---|---|---|---|---|---|---|
| NMR_T2 | 4 | 4.42 | 0.81 | 24.07 | 0.067 | 16.4 | 13.2 | NA | 9.7 | 6.9 | NA | 0 |
| NMR_T2 | 5 | 7.06 | 1.68 | 29.71 | 0.006 | NA | 16.4 | NA | 10.3 | 6.9 | NA | 1 |
| NMR_T2 | 6 | 5.48 | 2.34 | 12.85 | 0.000 | NA | 16.4 | NA | 9.2 | 8.6 | NA | 1 |
| NMR_T2 | 7 | 3.18 | 1.61 | 6.30 | 0.001 | NA | 15.5 | NA | 10.0 | 9.1 | 17.4 | 1 |
| NMR_T2 | 8 | 3.04 | 1.68 | 5.52 | 0.000 | NA | 16.4 | NA | 10.3 | 9.1 | 14.7 | 1 |
| NMR_T2 | 9 | 2.38 | 1.41 | 4.02 | 0.001 | NA | 15.5 | NA | 10.8 | 9.1 | 14.7 | 1 |
| NLR_T3 | 2 | 5.43 | 0.73 | 40.30 | 0.114 | NA | 16.4 | NA | 11.2 | 8.8 | NA | 0 |
| NLR_T3 | 3 | 2.43 | 0.88 | 6.72 | 0.094 | NA | NA | NA | 17.4 | 13.6 | NA | 0 |
| NLR_T3 | 4 | 1.69 | 0.78 | 3.65 | 0.189 | NA | NA | NA | 17.4 | 12.4 | NA | 0 |
| NLR_T3 | 5 | 1.72 | 0.85 | 3.45 | 0.137 | NA | NA | NA | 17.4 | 13.6 | NA | 0 |
| NLR_T3 | 6 | 1.51 | 0.79 | 2.89 | 0.215 | NA | NA | NA | 16.8 | 14.2 | NA | 0 |
| PLR_T3 | 120 | 2.69 | 0.52 | 13.94 | 0.246 | NA | 16.4 | NA | 17.4 | 12.4 | NA | 0 |
| PLR_T3 | 130 | 3.59 | 1.00 | 12.99 | 0.049 | NA | 16.4 | NA | 14.2 | 11.2 | NA | 1 |
| PLR_T3 | 140 | 3.22 | 1.07 | 9.75 | 0.041 | NA | 16.4 | NA | 13.3 | 11.2 | NA | 1 |
| PLR_T3 | 150 | 3.80 | 1.26 | 11.43 | 0.019 | NA | 16.4 | NA | 13.3 | 11.2 | NA | 1 |
| PLR_T3 | 160 | 2.54 | 0.88 | 7.34 | 0.089 | NA | 16.4 | NA | 14.2 | 12.2 | NA | 0 |
| PLR_T3 | 170 | 2.72 | 1.04 | 7.09 | 0.043 | NA | 16.4 | NA | 14.2 | 12.0 | NA | 1 |
| PLR_T3 | 180 | 2.99 | 1.15 | 7.79 | 0.027 | NA | NA | NA | 14.2 | 12.0 | NA | 1 |
| PLR_T3 | 190 | 2.90 | 1.23 | 6.85 | 0.017 | NA | NA | NA | 13.6 | 12.0 | NA | 1 |
| PLR_T3 | 200 | 2.61 | 1.18 | 5.78 | 0.017 | NA | 16.4 | NA | 14.2 | 12.0 | NA | 1 |
| LMR_T3 | 1 | 0.68 | 0.18 | 2.55 | 0.561 | 10.9 | 9.0 | NA | 14.3 | 9.7 | NA | 0 |

| | | | | | | | | | | | | |
|---|---|---|---|---|---|---|---|---|---|---|---|---|
| LMR_T3 | 2 | 1.22 | 0.58 | 2.56 | 0.599 | NA | 12.6 | NA | 16.8 | 13.8 | NA | 0 |
| LMR_T3 | 3 | 1.29 | 0.70 | 2.41 | 0.418 | NA | 15.5 | NA | 16.8 | 14.2 | NA | 0 |
| LMR_T3 | 4 | 1.06 | 0.60 | 1.88 | 0.835 | NA | 15.1 | NA | 16.8 | 14.3 | NA | 0 |
| NMR_T3 | 4 | 1830458197.20 | 0.00 | Inf | 0.083 | 16.4 | 16.4 | NA | NA | 11.2 | NA | 0 |
| NMR_T3 | 5 | 2440721077.35 | 0.00 | Inf | 0.008 | NA | 16.4 | NA | 11.2 | 10.3 | NA | 1 |
| NMR_T3 | 6 | 2.27 | 0.78 | 6.56 | 0.136 | NA | 16.4 | NA | 17.4 | 12.0 | NA | 0 |
| NMR_T3 | 7 | 1.85 | 0.80 | 4.27 | 0.155 | NA | 16.4 | NA | 17.4 | 14.2 | NA | 0 |
| NMR_T3 | 8 | 1.77 | 0.80 | 3.91 | 0.164 | NA | NA | NA | 17.4 | 14.2 | NA | 0 |
| NMR_T3 | 9 | 1.34 | 0.64 | 2.79 | 0.447 | NA | 16.4 | NA | 17.4 | 14.2 | NA | 0 |

L. Kaplan-Meier survival analysis of the BCT biomarkers at different cutoff values for application as prognostic biomarkers in the POPLAR cohort with high-risk.

| BCTbiomarker | BCTbiomarker.cutoff | HR.mean | HR.lci | HR.uci | logP | Ate.PFS.median | Ate.PFS.lci | Ate.PFS.uci | Dtx.PFS.median | Dtx.PFS.lci | Dtx.PFS.uci | logP<=0.05 |
|---|---|---|---|---|---|---|---|---|---|---|---|---|
| NMR_T2 | 4 | 1.02 | 0.70 | 1.47 | 0.936 | 7.3 | 4.9 | 10.2 | 9.2 | 8.6 | 12.2 | 0 |
| NMR_T2 | 5 | 1.03 | 0.70 | 1.50 | 0.887 | 6.8 | 4.2 | 10.2 | 9.1 | 8.6 | 12.4 | 0 |
| NMR_T2 | 6 | 0.74 | 0.49 | 1.13 | 0.160 | 4.4 | 4.1 | 7.8 | 9.4 | 8.6 | 12.5 | 0 |
| NMR_T2 | 7 | 0.77 | 0.49 | 1.20 | 0.244 | 4.4 | 4.1 | 8.0 | 9.2 | 8.3 | 12.5 | 0 |
| NMR_T2 | 8 | 0.64 | 0.38 | 1.06 | 0.076 | 4.2 | 2.9 | 7.3 | 10.2 | 7.0 | 12.9 | 0 |
| NMR_T2 | 9 | 0.68 | 0.39 | 1.19 | 0.176 | 4.5 | 3.9 | 8.4 | 10.2 | 7.0 | 13.6 | 0 |
| NLR_T3 | 2 | 1.04 | 0.64 | 1.69 | 0.866 | 8.5 | 6.9 | 11.4 | 12.1 | 9.7 | 14.2 | 0 |
| NLR_T3 | 3 | 0.89 | 0.51 | 1.56 | 0.684 | 8.4 | 4.4 | 11.1 | 12.0 | 9.2 | 14.4 | 0 |

| | | | | | | | | | | | | |
|---|---|---|---|---|---|---|---|---|---|---|---|---|
| NLR_T3 | 4 | 0.91 | 0.47 | 1.76 | 0.789 | 7.8 | 4.2 | 11.5 | 9.7 | 8.6 | 14.4 | 0 |
| NLR_T3 | 5 | 0.87 | 0.41 | 1.81 | 0.703 | 7.8 | 3.9 | 11.5 | 9.4 | 8.4 | NA | 0 |
| NLR_T3 | 6 | 0.80 | 0.34 | 1.86 | 0.598 | 8.1 | 3.9 | NA | 9.4 | 7.3 | NA | 0 |
| PLR_T3 | 120 | 1.10 | 0.68 | 1.80 | 0.695 | 8.5 | 6.8 | 11.4 | 10.7 | 9.2 | 13.8 | 0 |
| PLR_T3 | 130 | 1.10 | 0.66 | 1.84 | 0.723 | 8.5 | 6.8 | 11.4 | 10.2 | 9.1 | 14.3 | 0 |
| PLR_T3 | 140 | 1.05 | 0.62 | 1.79 | 0.848 | 8.4 | 5.8 | 11.4 | 10.0 | 9.1 | 14.3 | 0 |
| PLR_T3 | 150 | 0.97 | 0.57 | 1.65 | 0.907 | 8.3 | 5.5 | 11.0 | 10.0 | 9.1 | 14.3 | 0 |
| PLR_T3 | 160 | 1.00 | 0.58 | 1.71 | 0.996 | 8.3 | 5.5 | 11.1 | 10.0 | 9.1 | 14.3 | 0 |
| PLR_T3 | 170 | 0.97 | 0.55 | 1.70 | 0.913 | 8.3 | 5.5 | 11.4 | 10.0 | 8.8 | 14.4 | 0 |
| PLR_T3 | 180 | 0.90 | 0.51 | 1.59 | 0.726 | 7.8 | 4.2 | 11.0 | 10.0 | 8.8 | 14.4 | 0 |
| PLR_T3 | 190 | 0.94 | 0.52 | 1.71 | 0.847 | 8.3 | 4.2 | 11.0 | 10.0 | 8.8 | 14.4 | 0 |
| PLR_T3 | 200 | 1.00 | 0.52 | 1.92 | 0.995 | 8.4 | 5.5 | 11.4 | 9.2 | 8.6 | NA | 0 |
| LMR_T3 | 1 | 1.05 | 0.64 | 1.73 | 0.840 | 8.5 | 6.9 | 11.1 | 12.1 | 10.2 | 14.2 | 0 |
| LMR_T3 | 2 | 1.16 | 0.60 | 2.24 | 0.669 | 8.5 | 7.0 | 14.3 | 11.7 | 8.8 | NA | 0 |
| LMR_T3 | 3 | 1.15 | 0.49 | 2.73 | 0.751 | 9.0 | 4.3 | NA | 10.2 | 7.3 | NA | 0 |
| LMR_T3 | 4 | 1.23 | 0.42 | 3.61 | 0.712 | 9.5 | 4.3 | NA | 9.5 | 7.3 | NA | 0 |
| NMR_T3 | 4 | 1.01 | 0.63 | 1.63 | 0.967 | 8.5 | 6.9 | 11.0 | 12.1 | 9.2 | 14.2 | 0 |
| NMR_T3 | 5 | 0.92 | 0.56 | 1.50 | 0.730 | 8.4 | 6.8 | 10.9 | 12.3 | 9.7 | 14.3 | 0 |
| NMR_T3 | 6 | 0.81 | 0.47 | 1.40 | 0.449 | 7.8 | 4.9 | 10.2 | 12.2 | 9.2 | 14.4 | 0 |
| NMR_T3 | 7 | 0.78 | 0.41 | 1.47 | 0.432 | 7.3 | 4.2 | 11.1 | 12.2 | 8.8 | 14.4 | 0 |
| NMR_T3 | 8 | 0.65 | 0.33 | 1.26 | 0.191 | 5.8 | 4.1 | 11.1 | 12.2 | 8.8 | 14.4 | 0 |
| NMR_T3 | 9 | 0.61 | 0.30 | 1.26 | 0.181 | 5.8 | 4.1 | 11.1 | 12.2 | 8.8 | 14.4 | 0 |

M. Kaplan-Meier survival analysis of the BCT biomarkers at different cutoff values for application as prognostic biomarkers in the POPLAR cohort with low-risk.

| BCTbiomarker | BCTbiomarker.cutoff | HR.mean | HR.lci | HR.uci | logP | Ate.PFS.median | Ate.PFS.lci | Ate.PFS.uci | Dtx.PFS.median | Dtx.PFS.lci | Dtx.PFS.uci | logP<=0.05 |
|---|---|---|---|---|---|---|---|---|---|---|---|---|
| NMR_T2 | 4 | 1.08 | 0.29 | 4.09 | 0.909 | 7.5 | 4.3 | NA | 9.2 | 6.9 | NA | 0 |
| NMR_T2 | 5 | 0.93 | 0.33 | 2.61 | 0.888 | 8.3 | 7.0 | NA | 9.7 | 6.9 | NA | 0 |
| NMR_T2 | 6 | 2.08 | 1.04 | 4.18 | 0.045 | 11.5 | 9.0 | NA | 9.2 | 8.4 | 13.6 | 1 |
| NMR_T2 | 7 | 1.53 | 0.85 | 2.74 | 0.161 | 11.0 | 8.2 | NA | 9.2 | 8.6 | 13.6 | 0 |
| NMR_T2 | 8 | 1.58 | 0.95 | 2.63 | 0.081 | 10.9 | 8.3 | 14.4 | 9.2 | 8.4 | 11.6 | 0 |
| NMR_T2 | 9 | 1.27 | 0.80 | 2.03 | 0.312 | 9.0 | 6.9 | 11.5 | 9.2 | 8.4 | 11.6 | 0 |
| NLR_T3 | 2 | 1.33 | 0.28 | 6.32 | 0.725 | 9.6 | 4.9 | NA | 10.0 | 8.8 | NA | 0 |
| NLR_T3 | 3 | 1.21 | 0.52 | 2.84 | 0.666 | 10.4 | 8.2 | NA | 11.2 | 8.8 | NA | 0 |
| NLR_T3 | 4 | 1.11 | 0.57 | 2.19 | 0.761 | 9.4 | 7.3 | 14.3 | 12.0 | 10.3 | NA | 0 |
| NLR_T3 | 5 | 1.08 | 0.59 | 1.98 | 0.808 | 9.4 | 7.3 | 14.3 | 12.1 | 10.3 | 14.7 | 0 |
| NLR_T3 | 6 | 1.04 | 0.59 | 1.83 | 0.894 | 9.4 | 7.0 | 12.4 | 12.1 | 10.2 | 14.7 | 0 |
| PLR_T3 | 120 | 0.60 | 0.13 | 2.70 | 0.478 | 9.0 | 6.8 | NA | 12.4 | 10.3 | NA | 0 |
| PLR_T3 | 130 | 0.81 | 0.29 | 2.28 | 0.691 | 9.4 | 6.8 | NA | 11.8 | 10.3 | NA | 0 |
| PLR_T3 | 140 | 0.94 | 0.37 | 2.40 | 0.899 | 9.6 | 7.0 | NA | 12.2 | 10.3 | NA | 0 |
| PLR_T3 | 150 | 1.21 | 0.50 | 2.93 | 0.673 | 10.3 | 8.2 | NA | 12.2 | 10.3 | NA | 0 |
| PLR_T3 | 160 | 1.13 | 0.47 | 2.69 | 0.791 | 9.9 | 8.2 | NA | 12.2 | 10.3 | NA | 0 |
| PLR_T3 | 170 | 1.18 | 0.54 | 2.59 | 0.675 | 10.2 | 8.2 | NA | 12.0 | 10.3 | NA | 0 |
| PLR_T3 | 180 | 1.25 | 0.57 | 2.70 | 0.583 | 10.3 | 8.2 | NA | 12.0 | 10.3 | NA | 0 |
| PLR_T3 | 190 | 1.20 | 0.59 | 2.44 | 0.611 | 10.2 | 7.0 | NA | 12.0 | 10.3 | NA | 0 |

| | | | | | | | | | | | | |
|---|---|---|---|---|---|---|---|---|---|---|---|---|
| PLR_T3 | 200 | 1.14 | 0.60 | 2.17 | 0.689 | 9.6 | 7.0 | NA | 11.6 | 10.2 | 14.7 | 0 |
| LMR_T3 | 1 | 1.03 | 0.29 | 3.71 | 0.963 | 9.0 | 8.4 | NA | 9.4 | 8.4 | NA | 0 |
| LMR_T3 | 2 | 0.94 | 0.49 | 1.81 | 0.861 | 8.5 | 4.4 | 11.4 | 11.1 | 9.2 | 16.8 | 0 |
| LMR_T3 | 3 | 1.02 | 0.59 | 1.77 | 0.938 | 8.5 | 6.9 | 11.5 | 12.2 | 9.7 | 14.4 | 0 |
| LMR_T3 | 4 | 0.99 | 0.59 | 1.66 | 0.969 | 8.5 | 6.9 | 11.1 | 12.1 | 9.7 | 14.3 | 0 |
| NMR_T3 | 4 | 1.97 | 0.28 | 14.09 | 0.504 | NA | 4.3 | NA | 10.8 | 10.3 | NA | 0 |
| NMR_T3 | 5 | 3.50 | 0.76 | 16.11 | 0.107 | 17.8 | 11.0 | NA | 9.5 | 8.4 | NA | 0 |
| NMR_T3 | 6 | 1.58 | 0.64 | 3.89 | 0.338 | 11.5 | 9.6 | NA | 11.2 | 8.8 | NA | 0 |
| NMR_T3 | 7 | 1.25 | 0.62 | 2.53 | 0.537 | 10.4 | 8.3 | NA | 11.2 | 9.7 | NA | 0 |
| NMR_T3 | 8 | 1.22 | 0.62 | 2.41 | 0.579 | 10.2 | 8.2 | NA | 11.2 | 9.7 | NA | 0 |
| NMR_T3 | 9 | 1.14 | 0.58 | 2.21 | 0.707 | 10.2 | 7.8 | 12.4 | 11.2 | 9.7 | NA | 0 |

N. Kaplan-Meier OS survival analysis of the BCT biomarkers at different cutoff values for application as predictive biomarkers in the POPLAR cohort.

| BCTbiomarker | BCT biomarker. cutoff | cutoff | HR. mean | HR.lci | HR.uci | P | cutoff | HR. mean | HR.lci | HR.uci | P | Predictive ? |
|---|---|---|---|---|---|---|---|---|---|---|---|---|
| NMR_T2 | 4 | >cut-off | 1.27 | 0.84 | 1.93 | 0.250 | <cut-off | 4.42 | 0.81 | 24.07 | 0.085 | 0 |
| NMR_T2 | 5 | >cut-off | 1.16 | 0.76 | 1.76 | 0.492 | <cut-off | 7.06 | 1.68 | 29.71 | 0.008 | 0 |
| NMR_T2 | 6 | >cut-off | 0.83 | 0.53 | 1.31 | 0.430 | <cut-off | 5.48 | 2.34 | 12.85 | 0.000 | 0 |
| NMR_T2 | 7 | >cut-off | 0.80 | 0.49 | 1.31 | 0.377 | <cut-off | 3.18 | 1.61 | 6.30 | 0.001 | 0 |
| NMR_T2 | 8 | >cut-off | 0.64 | 0.37 | 1.10 | 0.108 | <cut-off | 3.04 | 1.68 | 5.52 | 0.000 | 0 |
| NMR_T2 | 9 | >cut-off | 0.61 | 0.33 | 1.12 | 0.112 | <cut-off | 2.38 | 1.41 | 4.02 | 0.001 | 0 |
| NLR_T3 | 2 | >cut-off | 1.06 | 0.62 | 1.82 | 0.834 | <cut-off | 5.43 | 0.73 | 40.30 | 0.098 | 0 |

| | | | | | | | | | | | | |
|---|---|---|---|---|---|---|---|---|---|---|---|---|
| NLR_T3 | 3 | >cut-off | 0.78 | 0.42 | 1.42 | 0.407 | <cut-off | 2.43 | 0.88 | 6.72 | 0.086 | 0 |
| NLR_T3 | 4 | >cut-off | 0.70 | 0.35 | 1.42 | 0.324 | <cut-off | 1.69 | 0.78 | 3.65 | 0.180 | 0 |
| NLR_T3 | 5 | >cut-off | 0.51 | 0.23 | 1.12 | 0.093 | <cut-off | 1.72 | 0.85 | 3.45 | 0.130 | 0 |
| NLR_T3 | 6 | >cut-off | 0.40 | 0.17 | 0.97 | 0.043 | <cut-off | 1.51 | 0.79 | 2.89 | 0.209 | 1 |
| PLR_T3 | 120 | >cut-off | 1.03 | 0.60 | 1.77 | 0.917 | <cut-off | 2.69 | 0.52 | 13.94 | 0.237 | 0 |
| PLR_T3 | 130 | >cut-off | 0.93 | 0.53 | 1.65 | 0.813 | <cut-off | 3.59 | 1.00 | 12.99 | 0.051 | 0 |
| PLR_T3 | 140 | >cut-off | 0.89 | 0.50 | 1.60 | 0.702 | <cut-off | 3.22 | 1.07 | 9.75 | 0.038 | 0 |
| PLR_T3 | 150 | >cut-off | 0.83 | 0.47 | 1.50 | 0.543 | <cut-off | 3.80 | 1.26 | 11.43 | 0.017 | 0 |
| PLR_T3 | 160 | >cut-off | 0.89 | 0.50 | 1.61 | 0.705 | <cut-off | 2.54 | 0.88 | 7.34 | 0.084 | 0 |
| PLR_T3 | 170 | >cut-off | 0.82 | 0.45 | 1.52 | 0.536 | <cut-off | 2.72 | 1.04 | 7.09 | 0.041 | 0 |
| PLR_T3 | 180 | >cut-off | 0.73 | 0.40 | 1.35 | 0.317 | <cut-off | 2.99 | 1.15 | 7.79 | 0.025 | 0 |
| PLR_T3 | 190 | >cut-off | 0.70 | 0.37 | 1.33 | 0.273 | <cut-off | 2.90 | 1.23 | 6.85 | 0.015 | 0 |
| PLR_T3 | 200 | >cut-off | 0.66 | 0.33 | 1.33 | 0.245 | <cut-off | 2.61 | 1.18 | 5.78 | 0.017 | 0 |
| LMR_T3 | 1 | >cut-off | 1.31 | 0.74 | 2.30 | 0.359 | <cut-off | 0.68 | 0.18 | 2.55 | 0.562 | 0 |
| LMR_T3 | 2 | >cut-off | 1.22 | 0.58 | 2.56 | 0.596 | <cut-off | 1.22 | 0.58 | 2.56 | 0.599 | 0 |
| LMR_T3 | 3 | >cut-off | 1.08 | 0.41 | 2.83 | 0.880 | <cut-off | 1.29 | 0.70 | 2.41 | 0.416 | 0 |
| LMR_T3 | 4 | >cut-off | 3.83 | 0.74 | 19.81 | 0.110 | <cut-off | 1.06 | 0.60 | 1.88 | 0.835 | 0 |
| NMR_T3 | 4 | >cut-off | 1.18 | 0.69 | 2.02 | 0.546 | <cut-off | 1830458197.20 | 0.00 | Inf | 0.999 | 0 |
| NMR_T3 | 5 | >cut-off | 1.05 | 0.60 | 1.82 | 0.870 | <cut-off | 2440721077.35 | 0.00 | Inf | 0.999 | 0 |
| NMR_T3 | 6 | >cut-off | 0.99 | 0.54 | 1.80 | 0.962 | <cut-off | 2.27 | 0.78 | 6.56 | 0.131 | 0 |
| NMR_T3 | 7 | >cut-off | 0.88 | 0.45 | 1.72 | 0.705 | <cut-off | 1.85 | 0.80 | 4.27 | 0.152 | 0 |
| NMR_T3 | 8 | >cut-off | 0.67 | 0.33 | 1.35 | 0.262 | <cut-off | 1.77 | 0.80 | 3.91 | 0.156 | 0 |
| NMR_T3 | 9 | >cut-off | 0.70 | 0.32 | 1.56 | 0.387 | <cut-off | 1.34 | 0.64 | 2.79 | 0.440 | 0 |

O. Kaplan-Meier PFS survival analysis of the BCT biomarkers at different cutoff values for application as predictive biomarkers in the POPLAR cohort.

| BCTbiomarker | BCT biomarker. cutoff | cutoff | HR.mean | HR.lci | HR.uci | P | cutoff | HR. mean | HR. lci | HR. uci | P | Predictive? |
|---|---|---|---|---|---|---|---|---|---|---|---|---|
| NMR_T2 | 4 | >cut-off | 1.02 | 0.70 | 1.47 | 0.936 | <cut-off | 1.08 | 0.29 | 4.09 | 0.910 | 0 |
| NMR_T2 | 5 | >cut-off | 1.03 | 0.70 | 1.50 | 0.887 | <cut-off | 0.93 | 0.33 | 2.61 | 0.888 | 0 |
| NMR_T2 | 6 | >cut-off | 0.74 | 0.49 | 1.13 | 0.165 | <cut-off | 2.08 | 1.04 | 4.18 | 0.039 | 0 |
| NMR_T2 | 7 | >cut-off | 0.77 | 0.49 | 1.20 | 0.249 | <cut-off | 1.53 | 0.85 | 2.74 | 0.154 | 0 |
| NMR_T2 | 8 | >cut-off | 0.64 | 0.38 | 1.06 | 0.082 | <cut-off | 1.58 | 0.95 | 2.63 | 0.076 | 0 |
| NMR_T2 | 9 | >cut-off | 0.68 | 0.39 | 1.19 | 0.180 | <cut-off | 1.27 | 0.80 | 2.03 | 0.306 | 0 |
| NLR_T3 | 2 | >cut-off | 1.04 | 0.64 | 1.69 | 0.866 | <cut-off | 1.33 | 0.28 | 6.32 | 0.717 | 0 |
| NLR_T3 | 3 | >cut-off | 0.89 | 0.51 | 1.56 | 0.685 | <cut-off | 1.21 | 0.52 | 2.84 | 0.660 | 0 |
| NLR_T3 | 4 | >cut-off | 0.91 | 0.47 | 1.76 | 0.790 | <cut-off | 1.11 | 0.57 | 2.19 | 0.758 | 0 |
| NLR_T3 | 5 | >cut-off | 0.87 | 0.41 | 1.81 | 0.705 | <cut-off | 1.08 | 0.59 | 1.98 | 0.807 | 0 |
| NLR_T3 | 6 | >cut-off | 0.80 | 0.34 | 1.86 | 0.599 | <cut-off | 1.04 | 0.59 | 1.83 | 0.893 | 0 |
| PLR_T3 | 120 | >cut-off | 1.10 | 0.68 | 1.80 | 0.694 | <cut-off | 0.60 | 0.13 | 2.70 | 0.503 | 0 |
| PLR_T3 | 130 | >cut-off | 1.10 | 0.66 | 1.84 | 0.721 | <cut-off | 0.81 | 0.29 | 2.28 | 0.695 | 0 |
| PLR_T3 | 140 | >cut-off | 1.05 | 0.62 | 1.79 | 0.848 | <cut-off | 0.94 | 0.37 | 2.40 | 0.899 | 0 |
| PLR_T3 | 150 | >cut-off | 0.97 | 0.57 | 1.65 | 0.907 | <cut-off | 1.21 | 0.50 | 2.93 | 0.668 | 0 |
| PLR_T3 | 160 | >cut-off | 1.00 | 0.58 | 1.71 | 0.996 | <cut-off | 1.13 | 0.47 | 2.69 | 0.790 | 0 |
| PLR_T3 | 170 | >cut-off | 0.97 | 0.55 | 1.70 | 0.913 | <cut-off | 1.18 | 0.54 | 2.59 | 0.672 | 0 |
| PLR_T3 | 180 | >cut-off | 0.90 | 0.51 | 1.59 | 0.727 | <cut-off | 1.25 | 0.57 | 2.70 | 0.577 | 0 |

| | | | | | | | | | | | | |
|---|---|---|---|---|---|---|---|---|---|---|---|---|
| PLR_T3 | 190 | >cut-off | 0.94 | 0.52 | 1.71 | 0.847 | <cut-off | 1.20 | 0.59 | 2.44 | 0.606 | 0 |
| PLR_T3 | 200 | >cut-off | 1.00 | 0.52 | 1.92 | 0.995 | <cut-off | 1.14 | 0.60 | 2.17 | 0.687 | 0 |
| LMR_T3 | 1 | >cut-off | 1.05 | 0.64 | 1.73 | 0.839 | <cut-off | 1.03 | 0.29 | 3.71 | 0.963 | 0 |
| LMR_T3 | 2 | >cut-off | 1.16 | 0.60 | 2.24 | 0.665 | <cut-off | 0.94 | 0.49 | 1.81 | 0.861 | 0 |
| LMR_T3 | 3 | >cut-off | 1.15 | 0.49 | 2.73 | 0.748 | <cut-off | 1.02 | 0.59 | 1.77 | 0.938 | 0 |
| LMR_T3 | 4 | >cut-off | 1.23 | 0.42 | 3.61 | 0.710 | <cut-off | 0.99 | 0.59 | 1.66 | 0.969 | 0 |
| NMR_T3 | 4 | >cut-off | 1.01 | 0.63 | 1.63 | 0.967 | <cut-off | 1.97 | 0.28 | 14.09 | 0.500 | 0 |
| NMR_T3 | 5 | >cut-off | 0.92 | 0.56 | 1.50 | 0.732 | <cut-off | 3.50 | 0.76 | 16.11 | 0.107 | 0 |
| NMR_T3 | 6 | >cut-off | 0.81 | 0.47 | 1.40 | 0.455 | <cut-off | 1.58 | 0.64 | 3.89 | 0.323 | 0 |
| NMR_T3 | 7 | >cut-off | 0.78 | 0.41 | 1.47 | 0.439 | <cut-off | 1.25 | 0.62 | 2.53 | 0.530 | 0 |
| NMR_T3 | 8 | >cut-off | 0.65 | 0.33 | 1.26 | 0.199 | <cut-off | 1.22 | 0.62 | 2.41 | 0.572 | 0 |
| NMR_T3 | 9 | >cut-off | 0.61 | 0.30 | 1.26 | 0.184 | <cut-off | 1.14 | 0.58 | 2.21 | 0.703 | 0 |

ABBREVIATIONS: NLR, neutrophile-to-lymphocyte ratio; PLR, platelet-to-lymphocyte ratio; NMR, neutrophile-to-monocyte ratio; OS, overall survival; PFS, progression-free survival; HiRsk, high-risk; LoRsk, low-risk; HR, hazard ratio; lci, lower confidence interval; uci. higher confidence interval; logP, log10 p-value; P, p-value.

## Supp Table 7.

**Comprehensive Evaulation of the BCTscore candidates at different fractions for application as prognostic and predictive biomarkers in the internal cohorts.**

| BCTscore | BCTscore. fraction | OAK. prognostic_ predictive_ deltaMedian | POPLAR. prognostic_ predictive_ deltaMedian | AND. prognostic_ predictive_ deltaMedian | OAK. predictive | POPLAR. predictive | Predictive? |
|---|---|---|---|---|---|---|---|
| BCTscore#1 | 0.1 | 0 | 0 | 0 | 0 | 0 | 0 |
| BCTscore#1 | 0.2 | 0 | 0 | 0 | 0 | 0 | 0 |
| BCTscore#1 | 0.25 | 0 | 2 | 1 | 0 | 0 | 0 |
| BCTscore#1 | 0.3 | 0 | 2 | 1 | 0 | 0 | 0 |
| BCTscore#1 | 0.4 | 0 | 2 | 1 | 0 | 0 | 0 |
| BCTscore#1 | 0.5 | 0 | 2 | 1 | 0 | 0 | 0 |
| BCTscore#1 | 0.6 | 0 | 2 | 1 | 0 | 0 | 0 |
| BCTscore#1 | 0.7 | 1 | 0 | 1 | 0 | 0 | 0 |
| BCTscore#1 | 0.75 | 2 | 0 | 1 | PFS | 0 | 1 |
| BCTscore#1 | 0.8 | 2 | 0 | 1 | PFS | 0 | 1 |
| BCTscore#1 | 0.9 | 2 | 0 | 1 | PFS | 0 | 1 |
| BCTscore#2 | 0.1 | 0 | 0 | 0 | 0 | 0 | 0 |
| BCTscore#2 | 0.2 | 0 | 0 | 0 | 0 | 0 | 0 |
| BCTscore#2 | 0.25 | 0 | 0 | 0 | 0 | 0 | 0 |
| BCTscore#2 | 0.3 | 0 | 0 | 0 | 0 | 0 | 0 |
| BCTscore#2 | 0.4 | 0 | 0 | 0 | 0 | 0 | 0 |

| | | | | | | | |
|---|---|---|---|---|---|---|---|
| BCTscore#2 | 0.5 | 2 | 2 | 2 | 0 | 0 | 0 |
| BCTscore#2 | 0.6 | 2 | 2 | 2 | 0 | 0 | 0 |
| BCTscore#2 | 0.7 | 0 | 0 | 0 | 0 | 0 | 0 |
| BCTscore#2 | 0.75 | 2 | 0 | 1 | PFS | 0 | 1 |
| BCTscore#2 | 0.8 | 2 | 0 | 1 | PFS | 0 | 1 |
| BCTscore#2 | 0.9 | 2 | 0 | 1 | PFS | 0 | 1 |
| BCTscore#3 | 0.1 | 0 | 0 | 0 | 0 | 0 | 0 |
| BCTscore#3 | 0.2 | 0 | 0 | 0 | 0 | 0 | 0 |
| BCTscore#3 | 0.25 | 0 | 2 | 1 | 0 | 0 | 0 |
| BCTscore#3 | 0.3 | 0 | 2 | 1 | 0 | 0 | 0 |
| BCTscore#3 | 0.4 | 0 | 2 | 1 | 0 | 0 | 0 |
| BCTscore#3 | 0.5 | 2 | 2 | 2 | 0 | 0 | 0 |
| BCTscore#3 | 0.6 | 2 | 2 | 2 | 0 | 0 | 0 |
| BCTscore#3 | 0.7 | 0 | 0 | 0 | 0 | 0 | 0 |
| BCTscore#3 | 0.75 | 2 | 0 | 1 | PFS | 0 | 1 |
| BCTscore#3 | 0.8 | 2 | 0 | 1 | PFS | 0 | 1 |
| BCTscore#3 | 0.9 | 2 | 0 | 1 | PFS | 0 | 1 |
| BCTscore#4 | 0.1 | 0 | 0 | 0 | 0 | 0 | 0 |
| BCTscore#4 | 0.2 | 0 | 0 | 0 | 0 | 0 | 0 |
| BCTscore#4 | 0.25 | 0 | 2 | 1 | 0 | 0 | 0 |
| BCTscore#4 | 0.3 | 0 | 2 | 1 | 0 | 0 | 0 |
| BCTscore#4 | 0.4 | 0 | 2 | 1 | 0 | 0 | 0 |
| BCTscore#4 | 0.5 | 0 | 2 | 1 | 0 | 0 | 0 |
| BCTscore#4 | 0.6 | 0 | 2 | 1 | 0 | 0 | 0 |

| | | | | | | | |
|---|---|---|---|---|---|---|---|
| BCTscore#4 | 0.7 | 0 | 0 | 0 | 0 | 0 | 0 |
| BCTscore#4 | 0.75 | 0 | 1 | 1 | PFS | OS | 2 |
| BCTscore#4 | 0.8 | 0 | 1 | 1 | PFS | OS | 2 |
| BCTscore#4 | 0.9 | 0 | 1 | 1 | PFS | OS | 2 |
| BCTscore#5 | 0.1 | 0 | 0 | 0 | 0 | 0 | 0 |
| BCTscore#5 | 0.2 | 0 | 0 | 0 | 0 | 0 | 0 |
| BCTscore#5 | 0.25 | 0 | 0 | 0 | 0 | 0 | 0 |
| BCTscore#5 | 0.3 | 0 | 0 | 0 | 0 | 0 | 0 |
| BCTscore#5 | 0.4 | 0 | 0 | 0 | 0 | 0 | 0 |
| BCTscore#5 | 0.5 | 0 | 2 | 1 | 0 | 0 | 0 |
| BCTscore#5 | 0.6 | 0 | 2 | 1 | 0 | 0 | 0 |
| BCTscore#5 | 0.7 | 0 | 2 | 1 | 0 | 0 | 0 |
| BCTscore#5 | 0.75 | 0 | 2 | 1 | 0 | OS | 1 |
| BCTscore#5 | 0.8 | 2 | 2 | 2 | PFS | OS | 2 |
| BCTscore#5 | 0.9 | 2 | 2 | 2 | PFS | OS | 2 |
| BCTscore#6 | 0.1 | 0 | 0 | 0 | 0 | 0 | 0 |
| BCTscore#6 | 0.2 | 0 | 0 | 0 | 0 | 0 | 0 |
| BCTscore#6 | 0.25 | 0 | 0 | 0 | 0 | 0 | 0 |
| BCTscore#6 | 0.3 | 0 | 0 | 0 | 0 | 0 | 0 |
| BCTscore#6 | 0.4 | 0 | 0 | 0 | 0 | 0 | 0 |
| BCTscore#6 | 0.5 | 0 | 2 | 1 | 0 | 0 | 0 |
| BCTscore#6 | 0.6 | 0 | 2 | 1 | 0 | 0 | 0 |
| BCTscore#6 | 0.7 | 0 | 2 | 1 | 0 | 0 | 0 |
| BCTscore#6 | 0.75 | 0 | 2 | 1 | 0 | OS | 1 |

| | | | | | | | |
|---|---|---|---|---|---|---|---|
| BCTscore#6 | 0.8 | 3 | 2 | 2 | OS | OS | 2 |
| BCTscore#6 | 0.9 | 3 | 2 | 2 | OS | OS | 2 |
| BCTscore#7 | 0.1 | 0 | 0 | 0 | 0 | 0 | 0 |
| BCTscore#7 | 0.2 | 0 | 0 | 0 | 0 | 0 | 0 |
| BCTscore#7 | 0.25 | 0 | 0 | 0 | 0 | 0 | 0 |
| BCTscore#7 | 0.3 | 0 | 2 | 1 | 0 | 0 | 0 |
| BCTscore#7 | 0.4 | 0 | 2 | 1 | 0 | 0 | 0 |
| BCTscore#7 | 0.5 | 0 | 2 | 1 | 0 | 0 | 0 |
| BCTscore#7 | 0.6 | 0 | 3 | 1 | 0 | 0 | 0 |
| BCTscore#7 | 0.7 | 0 | 3 | 1 | 0 | 0 | 0 |
| BCTscore#7 | 0.75 | 0 | 0 | 0 | 0 | 0 | 0 |
| BCTscore#7 | 0.8 | 2 | 2 | 2 | PFS | OS | 2 |
| BCTscore#7 | 0.9 | 2 | 2 | 2 | PFS | OS | 2 |
| BCTscore#8 | 0.1 | 0 | 0 | 0 | 0 | 0 | 0 |
| BCTscore#8 | 0.2 | 0 | 0 | 0 | 0 | 0 | 0 |
| BCTscore#8 | 0.25 | 0 | 0 | 0 | 0 | 0 | 0 |
| BCTscore#8 | 0.3 | 0 | 2 | 1 | 0 | 0 | 0 |
| BCTscore#8 | 0.4 | 0 | 2 | 1 | 0 | 0 | 0 |
| BCTscore#8 | 0.5 | 0 | 2 | 1 | 0 | 0 | 0 |
| BCTscore#8 | 0.6 | 1 | 3 | 2 | 0 | 0 | 0 |
| BCTscore#8 | 0.7 | 1 | 3 | 2 | 0 | 0 | 0 |
| BCTscore#8 | 0.75 | 0 | 0 | 0 | 0 | 0 | 0 |
| BCTscore#8 | 0.8 | 0 | 2 | 1 | PFS | OS | 2 |
| BCTscore#8 | 0.9 | 0 | 2 | 1 | PFS | OS | 2 |

| | | | | | | | |
|---|---|---|---|---|---|---|---|
| BCTscore#9 | 0.1 | 0 | 0 | 0 | 0 | 0 | 0 |
| BCTscore#9 | 0.2 | 0 | 0 | 0 | 0 | 0 | 0 |
| BCTscore#9 | 0.25 | 0 | 0 | 0 | 0 | 0 | 0 |
| BCTscore#9 | 0.3 | 0 | 0 | 0 | 0 | 0 | 0 |
| BCTscore#9 | 0.4 | 0 | 0 | 0 | 0 | 0 | 0 |
| BCTscore#9 | 0.5 | 0 | 0 | 0 | 0 | 0 | 0 |
| BCTscore#9 | 0.6 | 0 | 3 | 1 | PFS | 0 | 1 |
| BCTscore#9 | 0.7 | 0 | 0 | 0 | 0 | 0 | 0 |
| BCTscore#9 | 0.75 | 0 | 0 | 0 | 0 | 0 | 0 |
| BCTscore#9 | 0.8 | 2 | 0 | 1 | PFS | 0 | 1 |
| BCTscore#9 | 0.9 | 2 | 0 | 1 | PFS | 0 | 1 |
| BCTscore#10 | 0.1 | 0 | 0 | 0 | 0 | 0 | 0 |
| BCTscore#10 | 0.2 | 0 | 0 | 0 | 0 | 0 | 0 |
| BCTscore#10 | 0.25 | 0 | 0 | 0 | 0 | 0 | 0 |
| BCTscore#10 | 0.3 | 0 | 0 | 0 | 0 | 0 | 0 |
| BCTscore#10 | 0.4 | 0 | 0 | 0 | 0 | 0 | 0 |
| BCTscore#10 | 0.5 | 2 | 0 | 1 | 0 | 0 | 0 |
| BCTscore#10 | 0.6 | 0 | 3 | 1 | PFS | 0 | 1 |
| BCTscore#10 | 0.7 | 0 | 0 | 0 | 0 | 0 | 0 |
| BCTscore#10 | 0.75 | 0 | 0 | 0 | 0 | 0 | 0 |
| BCTscore#10 | 0.8 | 2 | 0 | 1 | PFS | 0 | 1 |
| BCTscore#10 | 0.9 | 2 | 0 | 1 | PFS | 0 | 1 |
| BCTscore#11 | 0.1 | 0 | 0 | 0 | 0 | 0 | 0 |
| BCTscore#11 | 0.2 | 0 | 0 | 0 | 0 | 0 | 0 |

| | | | | | | | |
|---|---|---|---|---|---|---|---|
| BCTscore#11 | 0.25 | 0 | 0 | 0 | 0 | 0 | 0 |
| BCTscore#11 | 0.3 | 0 | 2 | 1 | 0 | 0 | 0 |
| BCTscore#11 | 0.4 | 0 | 2 | 1 | 0 | 0 | 0 |
| BCTscore#11 | 0.5 | 0 | 2 | 1 | 0 | 0 | 0 |
| BCTscore#11 | 0.6 | 2 | 3 | 2 | PFS | 0 | 1 |
| BCTscore#11 | 0.7 | 1.47967146 | 0 | 1 | 0 | 0 | 0 |
| BCTscore#11 | 0.75 | 2 | 0 | 1 | PFS | 0 | 1 |
| BCTscore#11 | 0.8 | 2 | 0 | 1 | PFS | 0 | 1 |
| BCTscore#11 | 0.9 | 2 | 0 | 1 | PFS | 0 | 1 |
| BCTscore#12 | 0.1 | 0 | 0 | 0 | 0 | 0 | 0 |
| BCTscore#12 | 0.2 | 0 | 0 | 0 | 0 | 0 | 0 |
| BCTscore#12 | 0.25 | 0 | 0 | 0 | 0 | 0 | 0 |
| BCTscore#12 | 0.3 | 0 | 0 | 0 | 0 | 0 | 0 |
| BCTscore#12 | 0.4 | 0 | 2 | 1 | 0 | 0 | 0 |
| BCTscore#12 | 0.5 | 0 | 0 | 0 | 0 | 0 | 0 |
| BCTscore#12 | 0.6 | 2 | 3 | 2 | PFS | 0 | 1 |
| BCTscore#12 | 0.7 | 1.47967146 | 0 | 1 | 0 | 0 | 0 |
| BCTscore#12 | 0.75 | 0 | 0 | 0 | PFS | 0 | 1 |
| BCTscore#12 | 0.8 | 2 | 0 | 1 | PFS | 0 | 1 |
| BCTscore#12 | 0.9 | 2 | 0 | 1 | PFS | 0 | 1 |
| BCTscore#13 | 0.1 | 0 | 0 | 0 | 0 | 0 | 0 |
| BCTscore#13 | 0.2 | 0 | 0 | 0 | 0 | 0 | 0 |
| BCTscore#13 | 0.25 | 0 | 0 | 0 | 0 | 0 | 0 |
| BCTscore#13 | 0.3 | 0 | 0 | 0 | 0 | 0 | 0 |

| | | | | | | | |
|---|---|---|---|---|---|---|---|
| BCTscore#13 | 0.4 | 0 | 0 | 0 | 0 | 0 | 0 |
| BCTscore#13 | 0.5 | 0 | 0 | 0 | 0 | 0 | 0 |
| BCTscore#13 | 0.6 | 2 | 3 | 2 | PFS | 0 | 1 |
| BCTscore#13 | 0.7 | 2 | 3 | 2 | PFS | 0 | 1 |
| BCTscore#13 | 0.75 | 0 | 2 | 1 | 0 | OS | 1 |
| BCTscore#13 | 0.8 | 0 | 2 | 1 | 0 | OS | 1 |
| BCTscore#13 | 0.9 | 4 | 0 | 1 | OS | 0 | 1 |
| BCTscore#14 | 0.1 | 0 | 0 | 0 | 0 | 0 | 0 |
| BCTscore#14 | 0.2 | 0 | 0 | 0 | 0 | 0 | 0 |
| BCTscore#14 | 0.25 | 0 | 0 | 0 | 0 | 0 | 0 |
| BCTscore#14 | 0.3 | 0 | 0 | 0 | 0 | 0 | 0 |
| BCTscore#14 | 0.4 | 0 | 0 | 0 | 0 | 0 | 0 |
| BCTscore#14 | 0.5 | 2 | 0 | 1 | 0 | 0 | 0 |
| BCTscore#14 | 0.6 | 2 | 3 | 2 | 0 | 0 | 0 |
| BCTscore#14 | 0.7 | 2 | 3 | 2 | PFS | 0 | 1 |
| BCTscore#14 | 0.75 | 0 | 2 | 1 | 0 | OS | 1 |
| BCTscore#14 | 0.8 | 0 | 2 | 1 | 0 | OS | 1 |
| BCTscore#14 | 0.9 | 4 | 0 | 1 | OS | 0 | 1 |
| BCTscore#15 | 0.1 | 0 | 0 | 0 | 0 | 0 | 0 |
| BCTscore#15 | 0.2 | 0 | 0 | 0 | 0 | 0 | 0 |
| BCTscore#15 | 0.25 | 0 | 0 | 0 | 0 | 0 | 0 |
| BCTscore#15 | 0.3 | 0 | 2 | 1 | 0 | 0 | 0 |
| BCTscore#15 | 0.4 | 0 | 2 | 1 | 0 | 0 | 0 |
| BCTscore#15 | 0.5 | 0 | 2 | 1 | 0 | 0 | 0 |

| | | | | | | | |
|---|---|---|---|---|---|---|---|
| BCTscore#15 | 0.6 | 0 | 3 | 1 | 0 | 0 | 0 |
| BCTscore#15 | 0.7 | 2 | 3 | 2 | PFS | 0 | 1 |
| BCTscore#15 | 0.75 | 0 | 2 | 1 | 0 | OS | 1 |
| BCTscore#15 | 0.8 | 0 | 2 | 1 | 0 | OS | 1 |
| BCTscore#15 | 0.9 | 4 | 0 | 1 | OS | 0 | 1 |
| BCTscore#16 | 0.1 | 0 | 0 | 0 | 0 | 0 | 0 |
| BCTscore#16 | 0.2 | 0 | 0 | 0 | 0 | 0 | 0 |
| BCTscore#16 | 0.25 | 0 | 0 | 0 | 0 | 0 | 0 |
| BCTscore#16 | 0.3 | 0 | 0 | 0 | 0 | 0 | 0 |
| BCTscore#16 | 0.4 | 0 | 2 | 1 | 0 | 0 | 0 |
| BCTscore#16 | 0.5 | 0 | 0 | 0 | 0 | 0 | 0 |
| BCTscore#16 | 0.6 | 0 | 0 | 0 | 0 | 0 | 0 |
| BCTscore#16 | 0.7 | 2 | 3 | 2 | PFS | 0 | 1 |
| BCTscore#16 | 0.75 | 0 | 0 | 0 | 0 | 0 | 0 |
| BCTscore#16 | 0.8 | 0 | 2 | 1 | 0 | OS | 1 |
| BCTscore#16 | 0.9 | 4 | 0 | 1 | OS | 0 | 1 |

## Supp Table 8.

**Comprehensive Estimation of relative response rate of clinical benefit and objective response in the OAK and POPLAR cohorts.**

A. Estimation of relative response rate of clinical benefit and objective response in the OAK cohort.

| BCTscore | Ate.HiRsk.CB | Dtx.HiRsk.CB | HiRsk.CB.rate | Ate.LoRsk.CB | Dtx.LoRsk.CB | LoRsk.CB.rate | Ate.HiRsk.ORR | Dtx.HiRsk.ORR | HiRsk.ORR.rate | Ate.LoRsk.ORR | Dtx.LoRsk.ORR | LoRsk.ORR.rate |
|---|---|---|---|---|---|---|---|---|---|---|---|---|
| BCTscore #1 | 38% | 30% | 1.24 | 51% | 60% | 0.86 | 36% | 34% | 1.04 | 51% | 51% | 1.00 |
| BCTscore #2 | 38% | 30% | 1.25 | 51% | 60% | 0.85 | 33% | 34% | 0.96 | 48% | 51% | 0.95 |
| BCTscore #3 | 37% | 30% | 1.21 | 51% | 59% | 0.86 | 26% | 34% | 0.77 | 46% | 50% | 0.93 |
| BCTscore #4 | 38% | 30% | 1.24 | 50% | 60% | 0.84 | 25% | 34% | 0.73 | 44% | 51% | 0.88 |
| BCTscore #5 | 38% | 23% | 1.63 | 51% | 50% | 1.01 | 36% | 24% | 1.46 | 49% | 43% | 1.14 |
| BCTscore #6 | 38% | 23% | 1.64 | 50% | 50% | 0.99 | 33% | 24% | 1.35 | 46% | 43% | 1.07 |
| BCTscore #7 | 37% | 23% | 1.57 | 51% | 50% | 1.02 | 26% | 24% | 1.08 | 44% | 43% | 1.04 |
| BCTscore #8 | 38% | 23% | 1.61 | 50% | 50% | 1.00 | 25% | 24% | 1.03 | 42% | 43% | 0.99 |
| BCTscore #9 | 34% | 30% | 1.11 | 50% | 60% | 0.85 | 21% | 34% | 0.61 | 36% | 51% | 0.71 |

| | | | | | | | | | | | | |
|---|---|---|---|---|---|---|---|---|---|---|---|---|
| BCTscore #10 | 33% | 30% | 1.13 | 50% | 56% | 0.90 | 20% | 33% | 0.60 | 34% | 48% | 0.71 |
| BCTscore #11 | 38% | 30% | 1.23 | 52% | 60% | 0.87 | 34% | 34% | 1.00 | 51% | 51% | 1.02 |
| BCTscore #12 | 39% | 30% | 1.28 | 50% | 60% | 0.84 | 33% | 34% | 0.96 | 49% | 51% | 0.96 |
| BCTscore #13 | 33% | 23% | 1.39 | 50% | 48% | 1.04 | 21% | 24% | 0.87 | 32% | 41% | 0.78 |
| BCTscore #14 | 32% | 23% | 1.37 | 50% | 47% | 1.06 | 20% | 24% | 0.81 | 31% | 41% | 0.75 |
| BCTscore #15 | 38% | 40% | 0.94 | 51% | 59% | 0.87 | 34% | 43% | 0.80 | 50% | 53% | 0.95 |
| BCTscore #16 | 39% | 23% | 1.66 | 50% | 50% | 0.99 | 33% | 24% | 1.35 | 47% | 43% | 1.10 |

B. Estimation of relative response rate of clinical benefit of atezolizumab-treated patients in the OAK cohort.

| BCTscore | HiRsk.CB | LoRsk.CB | HiRsk.nonCB | LoRsk.nonCB | cohort | HiRsk.CB | LoRsk.CB |
|---|---|---|---|---|---|---|---|
| BCTscore#1 | 59 | 90 | 97 | 85 | OAK.Ate | 38% | 51% |
| BCTscore#2 | 56 | 93 | 91 | 91 | OAK.Ate | 38% | 51% |
| BCTscore#3 | 51 | 98 | 87 | 95 | OAK.Ate | 37% | 51% |
| BCTscore#4 | 50 | 99 | 82 | 100 | OAK.Ate | 38% | 50% |
| BCTscore#5 | 58 | 91 | 94 | 88 | OAK.Ate | 38% | 51% |
| BCTscore#6 | 55 | 94 | 88 | 94 | OAK.Ate | 38% | 50% |
| BCTscore#7 | 49 | 100 | 84 | 98 | OAK.Ate | 37% | 51% |

| | | | | | | | |
|---|---|---|---|---|---|---|---|
| BCTscore#8 | 48 | 101 | 79 | 103 | OAK.Ate | 38% | 50% |
| BCTscore#9 | 36 | 113 | 71 | 111 | OAK.Ate | 34% | 50% |
| BCTscore#10 | 34 | 115 | 68 | 114 | OAK.Ate | 33% | 50% |
| BCTscore#11 | 59 | 90 | 98 | 84 | OAK.Ate | 38% | 52% |
| BCTscore#12 | 58 | 91 | 91 | 91 | OAK.Ate | 39% | 50% |
| BCTscore#13 | 32 | 117 | 66 | 116 | OAK.Ate | 33% | 50% |
| BCTscore#14 | 30 | 119 | 63 | 119 | OAK.Ate | 32% | 50% |
| BCTscore#15 | 58 | 91 | 96 | 86 | OAK.Ate | 38% | 51% |
| BCTscore#16 | 57 | 92 | 89 | 93 | OAK.Ate | 39% | 50% |

C. Estimation of relative response rate of clinical benefit of docetaxel-treated patients in the OAK cohort.

| **BCTscore** | **HiRsk.CB** | **LoRsk.CB** | **HiRsk.nonCB** | **LoRsk.nonCB** | **cohort** | **HiRsk.CB** | **LoRsk.CB** |
|---|---|---|---|---|---|---|---|
| BCTscore#1 | 35 | 78 | 80 | 53 | OAK.Dtx | 30% | 60% |
| BCTscore#2 | 35 | 78 | 80 | 53 | OAK.Dtx | 30% | 60% |
| BCTscore#3 | 35 | 77 | 80 | 54 | OAK.Dtx | 30% | 59% |
| BCTscore#4 | 35 | 78 | 80 | 53 | OAK.Dtx | 30% | 60% |
| BCTscore#5 | 27 | 66 | 88 | 65 | OAK.Dtx | 23% | 50% |
| BCTscore#6 | 27 | 66 | 88 | 65 | OAK.Dtx | 23% | 50% |
| BCTscore#7 | 27 | 65 | 88 | 66 | OAK.Dtx | 23% | 50% |
| BCTscore#8 | 27 | 65 | 88 | 66 | OAK.Dtx | 23% | 50% |
| BCTscore#9 | 35 | 78 | 80 | 53 | OAK.Dtx | 30% | 60% |
| BCTscore#10 | 34 | 73 | 81 | 58 | OAK.Dtx | 30% | 56% |
| BCTscore#11 | 35 | 78 | 80 | 53 | OAK.Dtx | 30% | 60% |
| BCTscore#12 | 35 | 78 | 80 | 53 | OAK.Dtx | 30% | 60% |

| | | | | | | | |
|---|---|---|---|---|---|---|---|
| BCTscore#13 | 27 | 63 | 88 | 68 | OAK.Dtx | 23% | 48% |
| BCTscore#14 | 27 | 62 | 88 | 69 | OAK.Dtx | 23% | 47% |
| BCTscore#15 | 46 | 77 | 69 | 54 | OAK.Dtx | 40% | 59% |
| BCTscore#16 | 27 | 66 | 88 | 65 | OAK.Dtx | 23% | 50% |

D. Estimation of relative response rate of objective response of atezolizumab-treated patients in the OAK cohort.

| **BCTscore** | **HiRsk.ORR** | **LoRsk.ORR** | **HiRsk.nonORR** | **LoRsk.nonORR** | **cohort** | **HiRsk.ORR** | **LoRsk.ORR** |
|---|---|---|---|---|---|---|---|
| BCTscore#1 | 27 | 129 | 49 | 126 | OAK.Ate | 36% | 51% |
| BCTscore#2 | 25 | 122 | 51 | 133 | OAK.Ate | 33% | 48% |
| BCTscore#3 | 20 | 118 | 56 | 137 | OAK.Ate | 26% | 46% |
| BCTscore#4 | 19 | 113 | 57 | 142 | OAK.Ate | 25% | 44% |
| BCTscore#5 | 27 | 125 | 49 | 130 | OAK.Ate | 36% | 49% |
| BCTscore#6 | 25 | 118 | 51 | 137 | OAK.Ate | 33% | 46% |
| BCTscore#7 | 20 | 113 | 56 | 142 | OAK.Ate | 26% | 44% |
| BCTscore#8 | 19 | 108 | 57 | 147 | OAK.Ate | 25% | 42% |
| BCTscore#9 | 16 | 91 | 60 | 164 | OAK.Ate | 21% | 36% |
| BCTscore#10 | 15 | 87 | 61 | 168 | OAK.Ate | 20% | 34% |
| BCTscore#11 | 26 | 131 | 50 | 124 | OAK.Ate | 34% | 51% |
| BCTscore#12 | 25 | 124 | 51 | 131 | OAK.Ate | 33% | 49% |
| BCTscore#13 | 16 | 82 | 60 | 173 | OAK.Ate | 21% | 32% |
| BCTscore#14 | 15 | 78 | 61 | 177 | OAK.Ate | 20% | 31% |
| BCTscore#15 | 26 | 128 | 50 | 127 | OAK.Ate | 34% | 50% |
| BCTscore#16 | 25 | 121 | 51 | 134 | OAK.Ate | 33% | 47% |

E. Estimation of relative response rate of objective response of docetaxel-treated patients in the OAK cohort.

| BCTscore | HiRsk.ORR | LoRsk.ORR | HiRsk.nonORR | LoRsk.nonORR | cohort | HiRsk.CB | LoRsk.CB |
|---|---|---|---|---|---|---|---|
| BCTscore#1 | 24 | 89 | 46 | 87 | OAK.Dtx | 34% | 51% |
| BCTscore#2 | 24 | 89 | 46 | 87 | OAK.Dtx | 34% | 51% |
| BCTscore#3 | 24 | 88 | 46 | 88 | OAK.Dtx | 34% | 50% |
| BCTscore#4 | 24 | 89 | 46 | 87 | OAK.Dtx | 34% | 51% |
| BCTscore#5 | 17 | 76 | 53 | 100 | OAK.Dtx | 24% | 43% |
| BCTscore#6 | 17 | 76 | 53 | 100 | OAK.Dtx | 24% | 43% |
| BCTscore#7 | 17 | 75 | 53 | 101 | OAK.Dtx | 24% | 43% |
| BCTscore#8 | 17 | 75 | 53 | 101 | OAK.Dtx | 24% | 43% |
| BCTscore#9 | 24 | 89 | 46 | 87 | OAK.Dtx | 34% | 51% |
| BCTscore#10 | 23 | 84 | 47 | 92 | OAK.Dtx | 33% | 48% |
| BCTscore#11 | 24 | 89 | 46 | 87 | OAK.Dtx | 34% | 51% |
| BCTscore#12 | 24 | 89 | 46 | 87 | OAK.Dtx | 34% | 51% |
| BCTscore#13 | 17 | 73 | 53 | 103 | OAK.Dtx | 24% | 41% |
| BCTscore#14 | 17 | 72 | 53 | 104 | OAK.Dtx | 24% | 41% |
| BCTscore#15 | 30 | 93 | 40 | 83 | OAK.Dtx | 43% | 53% |
| BCTscore#16 | 17 | 76 | 53 | 100 | OAK.Dtx | 24% | 43% |

F. Estimation of relative response rate of clinical benefit and objective response in the POPLAR cohort.

| BCTscore | Ate. HiRsk.CB | Dtx. HiRsk.CB | HiRsk. CB.rate | Ate. LoRsk.CB | Dtx. LoRsk.CB | LoRsk. CB.rate | Ate. HiRsk.ORR | Dtx. HiRsk.ORR | HiRsk. ORR.rate | Ate. LoRsk.ORR | Dtx. LoRsk.ORR | LoRsk. ORR.rate |
|---|---|---|---|---|---|---|---|---|---|---|---|---|

| | | | | | | | | | | | |
|---|---|---|---|---|---|---|---|---|---|---|---|
| BCTscore#1 | 31% | 43% | 0.72 | 59% | 58% | 1.02 | 30% | 32% | 0.95 | 50% | 60% | 0.83 |
| BCTscore#2 | 29% | 43% | 0.67 | 55% | 58% | 0.95 | 25% | 32% | 0.79 | 47% | 60% | 0.78 |
| BCTscore#3 | 29% | 43% | 0.67 | 50% | 58% | 0.87 | 25% | 32% | 0.79 | 44% | 60% | 0.73 |
| BCTscore#4 | 26% | 43% | 0.61 | 50% | 58% | 0.87 | 25% | 32% | 0.79 | 42% | 60% | 0.71 |
| BCTscore#5 | 29% | 32% | 0.89 | 55% | 54% | 1.01 | 25% | 16% | 1.58 | 47% | 57% | 0.82 |
| BCTscore#6 | 26% | 32% | 0.81 | 50% | 54% | 0.93 | 20% | 16% | 1.27 | 44% | 57% | 0.77 |
| BCTscore#7 | 26% | 32% | 0.81 | 45% | 54% | 0.84 | 20% | 16% | 1.27 | 41% | 57% | 0.72 |
| BCTscore#8 | 24% | 32% | 0.74 | 43% | 54% | 0.80 | 20% | 16% | 1.27 | 38% | 57% | 0.66 |
| BCTscore#9 | 26% | 43% | 0.61 | 48% | 58% | 0.83 | 20% | 32% | 0.63 | 42% | 60% | 0.71 |
| BCTscore#10 | 24% | 39% | 0.61 | 45% | 58% | 0.79 | 15% | 26% | 0.57 | 41% | 60% | 0.68 |
| BCTscore#11 | 24% | 43% | 0.56 | 45% | 58% | 0.79 | 15% | 32% | 0.48 | 41% | 60% | 0.68 |
| BCTscore#12 | 21% | 43% | 0.50 | 45% | 58% | 0.79 | 15% | 32% | 0.48 | 39% | 60% | 0.66 |
| BCTscore#13 | 21% | 29% | 0.75 | 41% | 54% | 0.76 | 15% | 11% | 1.43 | 36% | 57% | 0.64 |
| BCTscore#14 | 19% | 29% | 0.67 | 39% | 54% | 0.72 | 10% | 11% | 0.95 | 35% | 57% | 0.61 |
| BCTscore#15 | 19% | 29% | 0.67 | 39% | 54% | 0.72 | 10% | 11% | 0.95 | 35% | 57% | 0.61 |
| BCTscore#16 | 43% | 32% | 1.33 | 57% | 54% | 1.06 | 50% | 16% | 3.17 | 50% | 57% | 0.88 |

G. Estimation of relative response rate of clinical benefit of atezolizumab-treated patients in the POPLAR cohort.

| BCTscore | HiRsk.CB | LoRsk.CB | HiRsk.nonCB | LoRsk.nonCB | cohort | HiRsk.CB | LoRsk.CB |
|---|---|---|---|---|---|---|---|
| BCTscore#1 | 13 | 26 | 29 | 18 | POPLAR.Ate | 31% | 59% |
| BCTscore#2 | 12 | 24 | 30 | 20 | POPLAR.Ate | 29% | 55% |
| BCTscore#3 | 12 | 22 | 30 | 22 | POPLAR.Ate | 29% | 50% |
| BCTscore#4 | 11 | 22 | 31 | 22 | POPLAR.Ate | 26% | 50% |
| BCTscore#5 | 12 | 24 | 30 | 20 | POPLAR.Ate | 29% | 55% |

| | | | | | | | |
|---|---|---|---|---|---|---|---|
| BCTscore#6 | 11 | 22 | 31 | 22 | POPLAR.Ate | 26% | 50% |
| BCTscore#7 | 11 | 20 | 31 | 24 | POPLAR.Ate | 26% | 45% |
| BCTscore#8 | 10 | 19 | 32 | 25 | POPLAR.Ate | 24% | 43% |
| BCTscore#9 | 11 | 21 | 31 | 23 | POPLAR.Ate | 26% | 48% |
| BCTscore#10 | 10 | 20 | 32 | 24 | POPLAR.Ate | 24% | 45% |
| BCTscore#11 | 10 | 20 | 32 | 24 | POPLAR.Ate | 24% | 45% |
| BCTscore#12 | 9 | 20 | 33 | 24 | POPLAR.Ate | 21% | 45% |
| BCTscore#13 | 9 | 18 | 33 | 26 | POPLAR.Ate | 21% | 41% |
| BCTscore#14 | 8 | 17 | 34 | 27 | POPLAR.Ate | 19% | 39% |
| BCTscore#15 | 8 | 17 | 34 | 27 | POPLAR.Ate | 19% | 39% |
| BCTscore#16 | 18 | 25 | 24 | 19 | POPLAR.Ate | 43% | 57% |

H. Estimation of relative response rate of clinical benefit of docetaxel-treated patients in the POPLAR cohort.

| BCTscore | HiRsk.CB | LoRsk.CB | HiRsk.nonCB | LoRsk.nonCB | cohort | HiRsk.CB | LoRsk.CB |
|---|---|---|---|---|---|---|---|
| BCTscore#1 | 12 | 15 | 16 | 11 | POPLAR.Dtx | 43% | 58% |
| BCTscore#2 | 12 | 15 | 16 | 11 | POPLAR.Dtx | 43% | 58% |
| BCTscore#3 | 12 | 15 | 16 | 11 | POPLAR.Dtx | 43% | 58% |
| BCTscore#4 | 12 | 15 | 16 | 11 | POPLAR.Dtx | 43% | 58% |
| BCTscore#5 | 9 | 14 | 19 | 12 | POPLAR.Dtx | 32% | 54% |
| BCTscore#6 | 9 | 14 | 19 | 12 | POPLAR.Dtx | 32% | 54% |
| BCTscore#7 | 9 | 14 | 19 | 12 | POPLAR.Dtx | 32% | 54% |
| BCTscore#8 | 9 | 14 | 19 | 12 | POPLAR.Dtx | 32% | 54% |
| BCTscore#9 | 12 | 15 | 16 | 11 | POPLAR.Dtx | 43% | 58% |
| BCTscore#10 | 11 | 15 | 17 | 11 | POPLAR.Dtx | 39% | 58% |

| | | | | | | | |
|---|---|---|---|---|---|---|---|
| BCTscore#11 | 12 | 15 | 16 | 11 | POPLAR.Dtx | 43% | 58% |
| BCTscore#12 | 12 | 15 | 16 | 11 | POPLAR.Dtx | 43% | 58% |
| BCTscore#13 | 8 | 14 | 20 | 12 | POPLAR.Dtx | 29% | 54% |
| BCTscore#14 | 8 | 14 | 20 | 12 | POPLAR.Dtx | 29% | 54% |
| BCTscore#15 | 8 | 14 | 20 | 12 | POPLAR.Dtx | 29% | 54% |
| BCTscore#16 | 9 | 14 | 19 | 12 | POPLAR.Dtx | 32% | 54% |

I. Estimation of relative response rate of objective response of atezolizumab-treated patients in the POPLAR cohort.

| BCTscore | HiRsk.ORR | LoRsk.ORR | HiRsk.nonORR | LoRsk.nonORR | cohort | HiRsk.ORR | LoRsk.ORR |
|---|---|---|---|---|---|---|---|
| BCTscore#1 | 6 | 33 | 14 | 33 | POPLAR.Ate | 30% | 50% |
| BCTscore#2 | 5 | 31 | 15 | 35 | POPLAR.Ate | 25% | 47% |
| BCTscore#3 | 5 | 29 | 15 | 37 | POPLAR.Ate | 25% | 44% |
| BCTscore#4 | 5 | 28 | 15 | 38 | POPLAR.Ate | 25% | 42% |
| BCTscore#5 | 5 | 31 | 15 | 35 | POPLAR.Ate | 25% | 47% |
| BCTscore#6 | 4 | 29 | 16 | 37 | POPLAR.Ate | 20% | 44% |
| BCTscore#7 | 4 | 27 | 16 | 39 | POPLAR.Ate | 20% | 41% |
| BCTscore#8 | 4 | 25 | 16 | 41 | POPLAR.Ate | 20% | 38% |
| BCTscore#9 | 4 | 28 | 16 | 38 | POPLAR.Ate | 20% | 42% |
| BCTscore#10 | 3 | 27 | 17 | 39 | POPLAR.Ate | 15% | 41% |
| BCTscore#11 | 3 | 27 | 17 | 39 | POPLAR.Ate | 15% | 41% |
| BCTscore#12 | 3 | 26 | 17 | 40 | POPLAR.Ate | 15% | 39% |
| BCTscore#13 | 3 | 24 | 17 | 42 | POPLAR.Ate | 15% | 36% |
| BCTscore#14 | 2 | 23 | 18 | 43 | POPLAR.Ate | 10% | 35% |
| BCTscore#15 | 2 | 23 | 18 | 43 | POPLAR.Ate | 10% | 35% |

| | | | | | | | |
|---|---|---|---|---|---|---|---|
| BCTscore#16 | 10 | 33 | 10 | 33 | POPLAR.Ate | 50% | 50% |

J. Estimation of relative response rate of objective response of docetaxel-treated patients in the POPLAR cohort.

| BCTscore | HiRsk.ORR | LoRsk.ORR | HiRsk.nonORR | LoRsk.nonORR | cohort | HiRsk.ORR | LoRsk.ORR |
|---|---|---|---|---|---|---|---|
| BCTscore#1 | 6 | 21 | 13 | 14 | POPLAR.Dtx | 32% | 60% |
| BCTscore#2 | 6 | 21 | 13 | 14 | POPLAR.Dtx | 32% | 60% |
| BCTscore#3 | 6 | 21 | 13 | 14 | POPLAR.Dtx | 32% | 60% |
| BCTscore#4 | 6 | 21 | 13 | 14 | POPLAR.Dtx | 32% | 60% |
| BCTscore#5 | 3 | 20 | 16 | 15 | POPLAR.Dtx | 16% | 57% |
| BCTscore#6 | 3 | 20 | 16 | 15 | POPLAR.Dtx | 16% | 57% |
| BCTscore#7 | 3 | 20 | 16 | 15 | POPLAR.Dtx | 16% | 57% |
| BCTscore#8 | 3 | 20 | 16 | 15 | POPLAR.Dtx | 16% | 57% |
| BCTscore#9 | 6 | 21 | 13 | 14 | POPLAR.Dtx | 32% | 60% |
| BCTscore#10 | 5 | 21 | 14 | 14 | POPLAR.Dtx | 26% | 60% |
| BCTscore#11 | 6 | 21 | 13 | 14 | POPLAR.Dtx | 32% | 60% |
| BCTscore#12 | 6 | 21 | 13 | 14 | POPLAR.Dtx | 32% | 60% |
| BCTscore#13 | 2 | 20 | 17 | 15 | POPLAR.Dtx | 11% | 57% |
| BCTscore#14 | 2 | 20 | 17 | 15 | POPLAR.Dtx | 11% | 57% |
| BCTscore#15 | 2 | 20 | 17 | 15 | POPLAR.Dtx | 11% | 57% |
| BCTscore#16 | 3 | 20 | 16 | 15 | POPLAR.Dtx | 16% | 57% |

ABBREVIATIONS: Ate, atezolizumab; Dtx, docetaxel; CB, clinical benefit; ORR, objective response rate; HiRsk, high-risk; LoRsk, low-risk.
